# Effects of Different Allotments of Avocados on the Nutritional Status of Families: A Cluster Randomized Controlled Trial

**DOI:** 10.3390/nu13114021

**Published:** 2021-11-11

**Authors:** Lorena S. Pacheco, Ryan D. Bradley, Julie O. Denenberg, Cheryl A. M. Anderson, Matthew A. Allison

**Affiliations:** 1 Department of Nutrition, Harvard T.H. Chan School of Public Health, 665 Huntington Avenue, Boston, MA 02115, USA; 2 The Herbert Wertheim School of Public Health and Human Longevity Science, University of California San Diego, 9500 Gilman Drive, La Jolla, CA 92093, USA; rybradley@health.ucsd.edu (R.D.B.); jdenenberg@health.ucsd.edu (J.O.D.); c1anderson@health.ucsd.edu (C.A.M.A.); 3 School of Public Health, San Diego State University, Hardy Tower Room 119, 5500 Campanile Drive, San Diego, CA 92182, USA; 4 Department of Family Medicine in the School of Medicine, University of California San Diego, 9500 Gilman Drive, La Jolla, CA 92093, USA; mallison@health.ucsd.edu

**Keywords:** avocado, *Persea americana*, promotora, plant-food, nutrition education, family intervention

## Abstract

Avocados are a nutrient-dense plant-food, but limited trial-derived evidence exists about the effects of avocado intake on family nutritional status. We investigated the impact of two levels of avocado allotment, plus a standard nutrition education intervention on the nutritional status of Hispanic/Latino families. Seventy-two families consisting of at least three members of ≥5 years of age and residing in the same home, free of severe chronic disease, not on specific diets, and self-identified of Hispanic heritage, were randomized to one of two levels of avocado allotment (low = 3/week/family or high = 14/week/family) for 6 months plus 12 bi-weekly nutrition education sessions. The primary outcomes included change in a family’s total energy and macro- and micronutrient intakes. Primary analysis was intention-to-treat with unpaired, two-sided *t*-tests to assess mean changes between groups at 6 months. At 6 months, the high avocado allotment group had a significant reduction in energy intake, carbohydrate, animal and vegetable protein, saturated and polyunsaturated fat, calcium, magnesium, sodium, potassium, iron, and vitamin D intakes (all *p* < 0.05). A high allotment of avocados significantly reduced self-reported energy intake by 29% kcal/family/day, compared to a 3% kcal/family/day reduction in families who received a low allotment. Culturally-appropriate plant-food interventions may alter the nutritional status of at-risk families.

## 1. Introduction

Adopting a healthy dietary pattern results in the consumption of nutrient-dense foods, while reducing the risk of chronic disease [1,2,3,4,5,6]. However, current dietary patterns of Americans are less than optimal. That is, the United States (US) population as a whole does not meet dietary guideline-recommended amounts of vegetables, fruits, and whole grains, and over-consumes refined grains, added sugars, and high-fat and sodium foods [7,8]. Compared to non-Hispanic Whites, Hispanics/Latinos have higher age-adjusted prevalence of obesity (44.8%) [9] while having lower intake of vitamin A and E, folate, magnesium, and potassium, as well as high intake levels of saturated fat and sodium [10]. Of particular relevance, the dietary quality of Hispanics/Latinos and other immigrants worsens as they become acculturated in the US and adopt a Western dietary pattern, which is higher in refined carbohydrates and animal-based fats [11]. This is particularly important since the population of Hispanics/Latinos, which consists of native- and foreign-born individuals immigrating from Latin America, the Caribbean, and Spain, are the second largest ethnic demographic in the US, comprising 18.1% of the population (5.8 million) [12].

Based on their nutrient profile, avocados could be a favorable component of a plant-based eating pattern, with half of a medium sized fruit providing up to 20% of the recommended daily fiber, 10% potassium, 5% magnesium, 15% folate, and 7.5 g of monounsaturated fatty acids (MUFA) [13]. However, there are gaps in our knowledge on the effects of avocado intake on nutritional status. In particular, addressing avocado integration into the dietary pattern of families of Hispanic/Latino heritage could help narrow the diet-related disparities for essential nutrients by highlighting the promotion of a traditionally consumed plant food. Although evidence supports a favorable effect of avocados on the cardiovascular risk profile in adults, with [14,15,16] and without [17,18] metabolic disease, it is important to establish the effects in ethnic populations, such as Hispanics/Latinos, who have different dietary patterns, and are, on average, at increased risk for metabolic diseases that predispose them to cardiovascular disease [19,20].

Avocados are a calorically-dense and contain saturated fat. As such, meticulous attention must be given to the delivery of nutrition education emphasizing how to appropriately incorporate avocados as part of a healthy dietary pattern, i.e., so that avocados do not excessively add to total caloric and/or saturated fat intake, or negatively influence snacking behavior (e.g., chips and guacamole), which could also increase sodium intake. Therefore, we conducted a clinical trial aimed to integrate avocados into the diet of families and measure the impact of this intervention on energy, macro- and micronutrient intakes. Our hypothesis was that the high avocado allotment would lead to an improved family-level nutritional status and improved cardiometabolic risk factors.

## 2. Materials and Methods

### 2.1. Study Design and Population

The present cluster, randomized controlled trial was conducted in San Diego County, California. The randomization unit was the family, which was assigned to one of two intervention groups: nutrition education with low avocado allotment (i.e., 3/family/week) or nutrition education with high avocado allotment (i.e., 14/family/week). They were followed for 6 months, with clinical visits at baseline, 3 and 6 months.

Inclusion criteria were: families with 3–8 family members ages > 5 years residing in the same home, willing to participate in the intervention, and self-identified of Hispanic/Latino heritage. Exclusion criteria were: families with members who had clinically severe chronic diseases requiring specific diets, avocado or latex allergy, current high consumers of avocados (i.e., >1 avocado/adult/day and >½ avocado/child/day), unwillingness to eat avocados, presence of pregnant females or females planning to become pregnant and/or intending to move within the next 6 months. Families with members < 5 years of age were included but young children were not counted towards the number of family members expected to participate in the intervention. Each family member was consented/assented into the study individually.

The study protocol and all study materials were approved by the Institutional Review Boards of the University of California San Diego and San Diego State University. This clinical trial was registered under clinicaltrials.gov study identifier NCT02903433.

### 2.2. Setting, Recruitment, Consent, and Randomization

**Figure 1:**
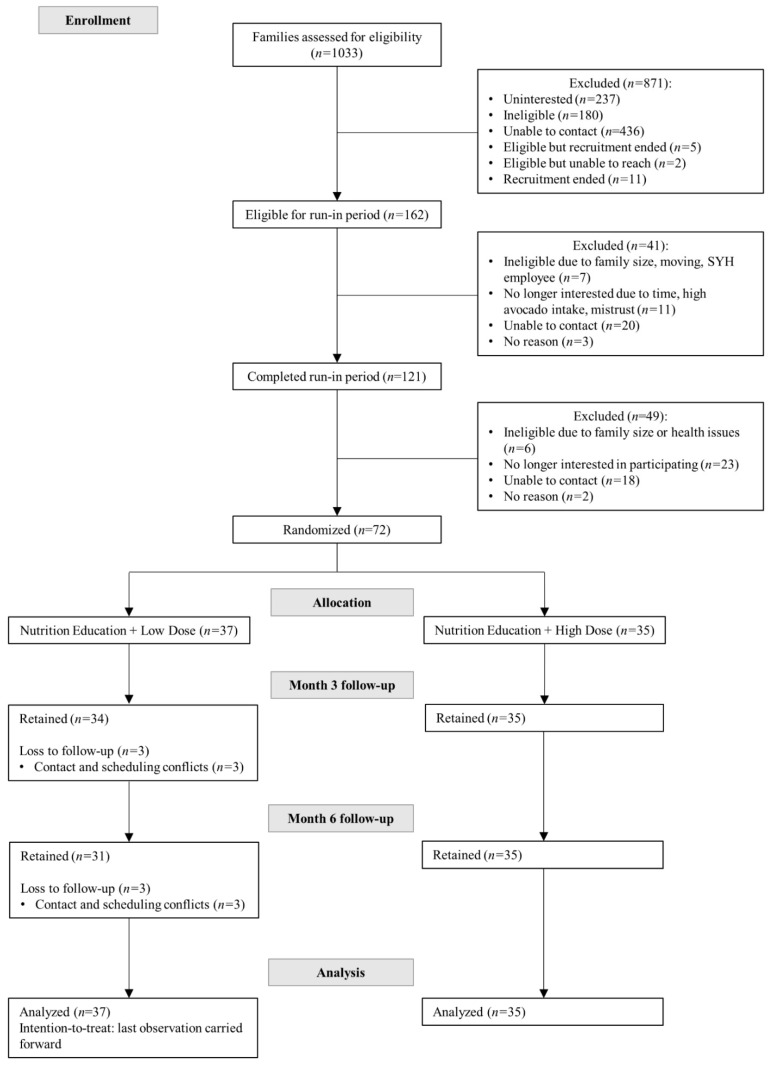
Consolidated standards of reporting trials (CONSORT) flow diagram of the Effects of Avocado Intake on the Nutritional Status of Families Trial.

Recruitment occurred between April 2017 and June 2018 (Figure 1). Electronic medical records at San Ysidro Health (SYH) services, a comprehensive health care system to over 90,000 registered patients in South and Central San Diego County, were queried to search for potential participants. Additional recruitment strategies included telephone calls, flyers, and in-person contacts during clinic health fairs.

A 14-day run in period assessed potential families’ commitment and adherence to study procedures, including participation in a home visit, willingness to schedule avocado delivery and nutrition education sessions, attendance to required clinic and laboratory visits, including all measurements and procedures. During this time, interested families were further screened for eligibility by a trained bilingual/bicultural female promotora during an in-home visit. Promotora is the Spanish term for “community health worker”, a trusted lay community member who receives training to provide elementary health education in the community without being a professional health care worker and who serves as a liaison between the community and traditional health care services. Specifically, families that completed a home visit, completed the questionnaires, and had their blood drawn, demonstrated a commitment and willingness to participate in the study and were scheduled for a baseline clinic visit where the head of the household (i.e., the family member who primarily shops for household groceries and prepares family meals) was identified.

Randomization of the family occurred at the baseline clinic visit using a computer-generated, blocked, randomization sequence. This was achieved with statistical analysis software (SAS) programming, using the RANUNI function procedure within the DATA statement, generating a random set of numbers for a specified range of observations (e.g., 1 to 75). Allocation concealment was accomplished by the randomization sequence only being accessed at the moment of randomization, and only one randomization assignment was visible at a time ensuring no advance notice of each assignment. Study arm assignment was implemented by the study coordinator. All staff members and study researchers, including principal investigators, were blinded to the randomization outcome. Promotoras and participants were unmasked for the intervention assignment due to the need to assist in the distribution of avocados and intervention delivery. During clinic visits, data collection was completed by study personnel who were not involved in delivery of the intervention.

### 2.3. Intervention

Families in both intervention groups received nutrition education and avocados over a 6-month period. The nutrition education was identical for both groups. Rather, it was standardized using 12 bi-weekly culturally and language appropriate nutrition education materials derived directly from resources provided by the United States Department of Agriculture MyPlate/MiPlato (http://www.choosemyplate.gov/; accessed on 11 March 2016) and aligned with the Dietary Guidelines for Americans (DGA) [7]. These standardized curriculum sessions were delivered by RDN-trained promotoras in participants’ homes with the goal of providing the participating families with tools and tips to improve diet quality and meet nutritional goals, yet not individually counseled on energy restriction or elimination of any foods.

The preparational work of the nutrition education included training of the promotoras by a Registered Dietitian Nutritionist (RDN) on the trial’s specific aims and intervention protocol. Each promotora received a training manual, which included the intervention protocol and materials for the session as well as a nutrition kit, which contained visual aids. The promotora was required to learn and be evaluated by the RDN on all the nutrition education lessons, following the stipulated language and delivery technique, before coming in contact with the participating families. The session design consisted of having at least the head of household of the family (for our purposes, the family member that was responsible for grocery shopping and meal preparation) join the promotora for a 20–30 min nutrition lesson where the promotora would go over the content of the assigned ‘lesson of the day’ pamphlet with the participant. Dialogue and questions were allowed during the session as the objective was for the participant to understand the content discussed. At the end of the session, the participant kept the pamphlet. Families did not receive compensation after each session since they collected compensation halfway through the study and after completion of study activities.

A total of 12 standard nutrition education lessons were specifically chosen to highlight particular MyPlate/MiPlato sections as ‘how to’ sessions including build a great plate, add more vegetables, add more fruit, make half of your grains whole grains, build a healthy meal and snacks for parents and children, choose good sources of protein, liven up meals with fruits and vegetables, make better beverage choices, fun and nutritious child-friendly meals, make good choices at school, work and on holidays. Families were also given a recipe booklet specifically focused on how to incorporate avocados in their diet. The recipe book illustrated a variety of dishes (entrees, sides, desserts) for the families to prepare to avoid monotony, and to encourage inclusion of avocados in new ways.

The dose of avocados in the low allocation group, 3/week, was based on the average reported intake in a survey of selected individuals in the target population (*n* = 101) and was conducted prior to starting the trial. The rationale for providing 3/week was to standardize the control arm and reduce potential variability that may have occurred if no standard had been provided, but to not increase or decrease families’ usual intake. Alternatively, the “dose” for the “high” intake allocation group (i.e., 14 avocados/week) was designed to allow for a robust increase in daily intake (by allowing for up to 2 avocados/family/day). This substantial increase therefore allowed for a potential increase in family energy intake up to 2625 kcal/week, if the avocados added to the current energy intake of the family. Incorporating a control group without avocado supplementation was considered, but determined to be impractical due to the typical intake by the target population (see above).

Promotoras delivered the allotted avocado amount per study arm to each participating family on a weekly basis throughout the duration of the intervention (6 months). They also provided an avocado care guide so the fruit would gradually mature throughout the week until the next delivery. Participating families were encouraged to not purchase additional avocados.

Study retention strategies involved regular contacts (telephone, email, correspondence, in-person) by study staff and monetary incentives ($100 to each family) provided midway and at the end of the study.

### 2.4. Measurements

The clinic visits at baseline, 3 and 6 months consisted of a dietary assessment, a participant survey packet, measurements of blood pressure and anthropometrics, measurement of physical activity, plus coordination of a blood draw at a local Laboratory Corporation of America Holdings (LabCorp) site. The participant survey packet asked about family socio-demographic characteristics, dietary and lifestyle factors and behaviors, and avocado consumption behavior at the family level and was only requested to be completed by head of household. Physical activity was measured with the global physical activity questionnaire (GPAQ) [21]. Blood pressure was measured using an automated Omron device three times at least one minute apart after the participant had been sitting for at least 5 min. Anthropometric measurements included weight, height, and waist circumference; height and weight were measured by a calibrated balance beam scale and stadiometer, respectively. Waist and hip circumference were measured with a semi-flexible tape measure, 2 cm above the iliac crest for waist and at the level of the widest circumference over the greater trochanters [22]. Participants visited a local LabCorp laboratory for the blood draw, scheduled within 5 days before or after the clinic visit, for measurement of the following: total lipid profile, fasting glucose, insulin, glycosylated hemoglobin (hemoglobin A1c), red blood cell magnesium, C-reactive protein, and plasma free fatty acids.

Dietary intake was assessed using a validated [23], self-administered, web-based, VioScreen (VioCare, Inc., Princeton, NJ, USA) food frequency questionnaire (FFQ) [24]. This assessment tool has been validated in adults, and a recent pilot in the pediatric population highlighted necessary modifications to tailor the questionnaire in children and adolescents [25]. Viocare Inc worked with the investigative team and incorporated study population-specific consumption foods, as well as avocado (individual level) as intervention food. The web-based FFQ worked as a standard FFQ (paper-based) asking about the amount and frequency of a food item. However, it incorporated additional questions in an interviewer–interviewee format, as well as displaying visual aids of appropriate serving sizes to assist participants’ estimates. Research associates instructed all participants on FFQ usage and supported those who required or desired assistance with the FFQ.

The Avocado Daily Diary was developed for this study, completed by the head of household and returned to the promotora during the bi-weekly home visits or weekly avocado delivery encounters. This instrument was designed by our research team to specifically capture avocado daily consumption of the family (not individually) and determine intervention adherence. A copy of the Avocado Daily Diary has been provided in the Supplemental Material file (Table S1).

The head of household completed all assessments. Non-head of household adult family members completed all assessments except for the participant survey packet. Children and adolescents only completed the GPAQ, dietary assessment, and measurements of blood pressure and anthropometrics.

### 2.5. Outcomes

The primary outcomes were total energy intake and nutritional status assessed at the family level. Measures included total intake of energy in kilocalories (kcal), macronutrients (carbohydrate, protein, fat, and dietary fiber) and micronutrients (vitamins C, D, E, folate, calcium, magnesium, sodium, potassium, and iron) plus the 2015 Healthy Eating Index (HEI-2015), calculated using the simple HEI scoring algorithm method [26]. We also assessed food group consumption patterns by examining intake of specific food groups (fruits; vegetables; dairy and non-dairy; nuts, animal and vegetable protein sources including red and processed meats; whole and refined grains; sugar; and oils). These measures were derived from the VioScreen FFQ. The values for total intake of energy and each nutrient were summed together per family, as this was the unit of analysis.

Secondary outcomes were cardiometabolic risk indicators assessed in adult participants and included: calculated body mass index (BMI) in adults, waist-to-height ratio in children and adolescents, waist circumference, blood pressure, lipids (total cholesterol, high-density lipoproteins (HDL), low-density lipoproteins (LDL), very low-density lipoproteins (VLDL), and triglycerides), glucose, insulin, hemoglobin A1c, calculated homeostatic model assessment of insulin resistance (HOMA-IR), and c-reactive protein. Several nutritional biomarkers, including plasma free fatty acids and red blood cell (RBC) magnesium, were also measured. Secondary outcomes in adolescents and children included calculated BMI, waist circumference, and blood pressure.

### 2.6. Intervention Adherence

Intervention adherence was determined using a customized Avocado Daily Diary designed to collect data on daily intake by all family members. Family weekly avocado consumption was calculated based on the number of avocados delivered, consumed, and remaining unconsumed by each family. A continuous adherence value was calculated based on amount consumed by family divided by intervention group intake goal.

### 2.7. Statistical Analyses

The sample size and power were based on our primary outcome of total intake of energy in kcal/family/day. A sample size of 60 families provided 80% power to detect a 375-kcal difference (equivalent to 1.5 medium avocados/family/day) at an alpha of 0.05 and a standard deviation (SD) of up to 500 kcal/family/day. To allow for up to 15% attrition and a final evaluable sample of 60 families, we aimed to randomize 70 total families.

Descriptive statistics were used to characterize the study population by intervention group. Continuous variables were expressed as mean and SD, while categorical variables were expressed as frequencies and percentages. Normality was evaluated for all continuous variables. For all outcomes, the primary analyses were conducted with an intention-to-treat approach, without covariate adjustment or intervention adherence. The Chi square test was used for comparisons of proportions derived from categorical variables, and 2-sided *t*-tests were used to assess mean differences in continuous variables between intervention groups. Changes from baseline to month 3 and from baseline to month 6 for the primary secondary outcomes were determined, and mean differences between intervention groups were assessed with 2-sided *t*-tests. Mean difference and standard deviation (SD or 95% confidence intervals (CI) are presented, where appropriate. Missing outcome data were imputed using the last observation carried forward method. For analyses of macro- and micronutrient intakes, we further adjusted for baseline total energy intake in separate analysis of covariance (ANCOVA) models.

As a sensitivity analysis, we performed per protocol adherence analysis, which considered intervention adherence and was applied to both primary and secondary outcomes on participants who completed the study. Family intervention adherence was estimated using the Avocado Daily Diary and calculated based on the number of avocados delivered, consumed, and remaining unconsumed by each family. A continuous adherence value was calculated based on amount consumed by family divided by intervention group intake goal. Complete adherence was defined as complete consumption of avocado allotment per week per family. ANCOVA, with intervention adherence as a covariate, was then used to compare total energy and macro- and micronutrient intakes by intervention groups.

In addition to intervention adherence adjustment, we further adjusted for baseline total energy intake as in the primary analysis. Age-specific subgroup analyses were also performed.

Secondary analyses included energy-adjusted macro- and micronutrients before determining mean difference between baseline and month 3 and baseline and month 6. The energy-adjusted methodology used included nutrient densities expressed as a proportion of energy (i.e., % kcal from fat) for macronutrients carbohydrate, protein, and fat. For micronutrients, food groups, as well as macronutrients, nutrient density was determined as intake (in appropriate units)/1000 kcal.

All *p* values presented are from 2-tailed analyses; *p* values of less than 0.05 were considered statistically significant. Analyses were conducted with SAS version 9.4 (SAS Institute Inc, Cary, NC, USA).

## 3. Results

Between 11 April 2017 and 27 June 2018, a total of 72 families (*n* = 37 in the low avocado allotment group and *n* = 35 in the high avocado allotment group) were randomized into this study, resulting in the participation of 231 individuals (Figure 1). Sixty-six families (91.7%) completed the study, with a dropout rate of 16.2% in the low avocado allotment group and 0% in the high avocado allotment group (*p* = 0.03). The main reasons for dropout were time consuming trial activities, scheduling conflicts, and difficulty contacting families. There were no statistically significant differences in socio-demographic characteristics with those families who remained in the study (Supplemental Table S2).

The average family size was three (SD 0.5; range 3–5 members), and almost half of the enrolled families reported a family annual income < $30,000 (Table 1). All but one head of the household was female (99%). Twenty-five percent of household members were children (mean age 9.3 (SD 2.1); range 5–12 years), 14% were adolescents (mean age 15.8 (SD 1.2); range 13–17 years), and 30% were non-head of household adults. Eighty-three percent of heads of households were born in Mexico and, on average, had lived in the US for 17.3 (SD 12.6) years. The majority were married or cohabitating, homemakers, and their highest educational attainment was an associate’s degree. Study heads of households had a mean age of 45.5 (SD 9.9) and other adults had a mean age of 41.4 years.

Study heads of households had a mean age of 45.5 (SD 9.9) and other adults had a mean age of 41.4 (SD 19.1; range 18–88 years) (Table 1). Study adults had a BMI of 30.4 (SD 6.4) kg/m^2^, and blood pressure within normal range (systolic, 118.3 (SD 17.6) mmHg and diastolic, 71.9 (SD 10.3) mmHg) (Table 2). Adolescents and children had a mean waist-height ratio of 0.5 cm (SD 0.1).

At baseline, carbohydrates constituted about half of the families’ daily total kcal, followed by 33% from fat, and 16% from protein (Table 2). This macronutrient distribution was consistent among all subgroups (Supplemental Table S3) and until the end of the trial (Supplemental Table S4). More specifically, in the low vs. high avocado allotment groups, the mean 6-month macronutrient distributions were 49% vs. 49% for carbohydrates, 35% vs. 36% for fat, and 16% vs. 17% for protein, respectively.

A reduction in self-reported family total energy was observed in both intervention groups at 3 and 6 months, with a greater reduction among high avocado allotment families at 6 months. The mean (95% CI) 6-month change in the family’s total energy intake was −259.0 (95% CI −958.0, 440.0) kcal/day for the low avocado allotment group and −2143.1 (95% CI −3286.5, −999.8) kcal/day for the high avocado allotment group (Table 3). This between-group mean difference was significantly different (*p* = 0.01).

Multiple between-group mean family differences were observed at 6-months, including carbohydrate, protein, and fat intakes (*p* ≤ 0.04 for all) (Table 3). Mean intake of animal and vegetable proteins, as well as MUFA and polyunsaturated fatty acids (PUFA), and saturated fat intakes were reduced in the high avocado allotment families. The mean 6-month differences between intervention groups for these nutrients, with the exception of MUFA intake, were significantly different (*p* ≤ 0.01 for all). Significant reductions in several micronutrients were also observed in the high avocado allotment families, including vitamin D, calcium, magnesium, potassium, sodium, and iron (*p* < 0.05 for all). The mean between-group difference for overall family-level HEI-2015 score at 6 months was −7.2 (95% CI −16.6, 2.1), with higher scores favoring the high avocado allotment group, and while suggestive, the difference did not reach statistical significance (*p* = 0.13). Age-specific subgroup intention-to-treat analysis results are reported in Supplemental Tables S5–S8. The majority of these findings persisted when adjusting macro- and micronutrients for baseline total energy intake.

Regarding differences in particular food groups, there were significant between-group mean differences for dairy, refined grains, chicken and eggs, and red meat food groups at 6 months (*p* ≤ 0.02 for all) (Table 4). Intake of these food groups were significantly lower in the high avocado allotment families. At 6 months in the high avocado families, heads of households significantly increased their intake of fruit (*p* = 0.02) and significantly reduced their intake of dairy (*p* = 0.04) and non-head of household trial adults significantly reduced their consumption of refined grains, chicken and eggs, fish, red meat, and oils (*p* ≤ 0.02 for all). Adolescents and children in the high avocado allotment families significantly reduced their whole grain intake (*p* = 0.05) and refined grains (*p* = 0.03), respectively (Supplemental Tables S9–S12). These results persisted after accounting for baseline total energy intake.

Secondary analysis findings are shown in Supplemental Tables S4 and S13. The mean energy-adjusted 6-month difference following ITT analysis in the proportion of fat between intervention groups was significantly different (*p* = 0.05) (Supplemental Table S4). Additionally, families randomized to the high avocado allotment group significantly increased their mean energy-adjusted intakes of dietary fiber, MUFA, potassium, vitamin E, and folate, in comparison to those families randomized to the low avocado allotment group following ITT. These findings remained after following per protocol adherence analysis (data not shown). Mean energy-adjusted 6-month differences in cup equivalents of fruit and vegetables between intervention groups was significantly different (*p* ≤ 0.01) (Supplemental Table S13).

**Figure 2:**
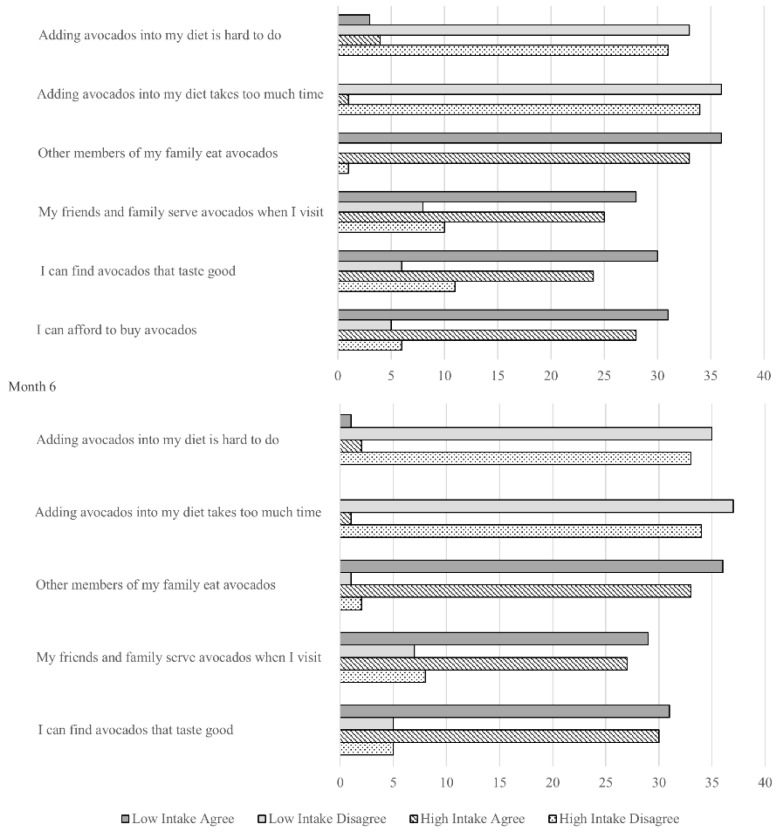
Avocado consumption behavior at baseline and 6 months in the Effects of Avocado Intake on the Nutritional Status of Families Trial. The question: “I can afford to buy avocados” was not applicable at 6 months since the study team supplied families with avocados as part of the intervention. All between-group differences *p*-values > 0.05.

Avocado consumption behavior at baseline and 6 months is presented in Figure 2. There were no significant differences between study groups at baseline and after 6 months (*p* > 0.05 for all). Suggesting acceptability of the intervention, participating families reported they could afford to buy avocados, readily add them to their diet, and include them as part of family gatherings.

At 6 months, BMI remained unchanged in both study groups, while waist circumference in both study groups increased modestly (Table 5). There were few significant changes in blood pressure except that systolic blood pressure increased slightly in adults in the high avocado allotment group. Specifically, the mean 6-month between-group difference was 5.9 (95% CI 1.9, 9.8) mmHg (*p* = 0.004), which was mostly due to the decrease seen in the low avocado allotment group (mean −5.5 (95% CI −8.5, −2.6) mmHg).

Lipids and glycemia biomarkers were not significantly different in the high avocado allotment group at 6 months (Table 6). Free fatty acids and RBC magnesium marginally increased in the high allotment adult group after 6 months, with a significant mean between-group difference in RBC magnesium of 0.36 (95% CI 0.09, 0.62) mg/dL (*p* = 0.010) for those tested (*n* = 33).

Greater than 80% adherence was met by 95% and 83% of low and high avocado allotment families at month 6, respectively. A total of 61 families (85%) had ≥80% continuous adherence to the intervention protocol throughout the duration of the study, corresponding to 92% and 77% for low and high avocado allotment families, respectively. Per protocol analyses suggested larger differential changes in family total energy and macronutrient intakes at 6 months, with these differences being statistically significant (*p* < 0.05 for all; Table 7). With the exception of sodium, mean differences in micronutrient intake at 6 months (calcium, potassium, magnesium, iron, sodium, and vitamin D) remained statistically significant after following per protocol adherence analyses. Mean differences in family food group intakes at 6 months persisted following per protocol adherence analyses (Table 8). The per protocol adherence analyses of secondary outcomes provided similar results to the intention-to-treat analyses, with no additional findings (data not shown). Age-specific per protocol adherence analyses nutrient and food group results were also conducted. The findings were similar to the intention-to-treat analyses (data not shown). Finally, there were no material differences in these results after adjustment for baseline total energy intake. There were no harms or unintended effects reported by study participants.

## 4. Discussion

To our knowledge, this is the first randomized controlled trial to test the effect of a single plant-food intervention on family energy intake, without dietary eliminations or restrictions or counseling on energy intake. We found that a high allotment of avocados (14/family/week) significantly reduced self-reported energy intake by 29% kcal/family/day, compared to a 3% kcal/family/day reduction in families who received a low (3 avocados/family/week) allotment. The findings have potentially important implications for Hispanics/Latinos who have a high burden of obesity, placing them at higher risk for metabolic and cardiovascular diseases [20,27]. Notably, although the lack of change in BMI or waist circumference in either group reduces our confidence in the self-reported values for daily energy, and while the higher allotment of avocados may have modified the intake of specific micronutrient and foods, there is no evidence the resulting changes adversely affected energy intake or body weight.

Interestingly, although we did not observe significant increases in either MUFA or dietary fiber intakes in analyses unadjusted for changes in energy intake, energy-adjusted secondary analyses demonstrated significant changes in MUFA and fiber (plus potassium and folate), as would be hypothesized based on the nutrient profile of avocados. It has been previously demonstrated that fat and dietary fiber induce a ceiling effect on satiety [28,29]. Fats and some dietary fibers appear to impact total energy intake by affecting gastrointestinal functions, including slowing gastric emptying by adding bulk and viscosity, regulating glucose and insulin reactions, prolonging nutrient absorption, and modifying appetite–satiety gastrointestinal peptide hormones [29,30,31]. Specifically, one medium size Haas avocado (~136 g without skin and seed) contains 21 g of fat (13 g oleic acid—MUFA) and 9.2 g dietary fiber, and has both a medium energy density of 1.7 kcal/g and a viscose water, dietary fiber, and fruit oil matrix that appears to enhance satiety [14,28]. In this regard, and among overweight and moderately obese adults, the addition of half an avocado to a lunch meal increased satiety for over 5 h, and inhibited desire to continue eating, compared to a meal without avocado [28]. Similarly, the work of Zhu et al., among overweight and obese adults, found the replacement of carbohydrate in a high-carbohydrate meal with fat- and fiber-rich whole avocado without increasing energy suppressed hunger and improved satiety, while increasing satisfaction (satiety) feelings for over 6 h, compared to a control meal (low-fat, high-carbohydrate) [32]. Interestingly, Zhu et al. reported that satiety induced by whole avocado replacement was mostly due to impact on responses to gastrointestinal peptide YY hormone [32].

Given the reported reductions in total energy intake at the family and individual level, the reported decreases in macro- and micronutrients were expected. However, high avocado intake families consumed significantly less protein of animal origin (31.9% reduction, equivalent to a decrease of ~8 ounces, per family) primarily from chicken and eggs, and red and processed meats, which are usually higher in fat and saturated fat (31.6% reduction per family). These two nutrients tend to be high in Western dietary patterns and current guidelines advise reduced intake of these sources of macronutrients [7]. Although reduced, the macronutrient distribution was consistent at the three time points, at both family and individual levels, and between groups (49% carbohydrate, 35% fat, 17% protein), and similar to those observed in Mexican-heritage adults in the Hispanic Community Health Study/Study of Latinos, the largest community-based cohort of Hispanics/Latino in the US (50% carbohydrate, 32% fat, 18% protein) [33].

Correspondingly, and in general, the families who had a high avocado allocation reported decreased calcium, magnesium, potassium, iron, sodium, and vitamin D consumption. Because avocados contain potassium and magnesium [14], we did not anticipate this change. This effect could be associated with overall reduced caloric intake, leading to general reductions in nutrients. Nevertheless, the findings are contrary to results observed in a cross-sectional analysis in the National Health And Nutrition Examination Survey (NHANES), where, compared to non-consumers, avocado consumers (average intake half avocado/day) had significantly higher intakes of MUFA, dietary fiber, magnesium, and potassium (*p* < 0.0001), and no significant differences in total energy intake and sodium [34].

At baseline, participating families were, on average, consuming almost two times more sodium than recommended by the DGA [7]. We observed a significant between-group difference of −2664.3 mg sodium/family/day (*p* = 0.05) at 6 months, with a greater reduction in high avocado intake families. High avocado intake families reduced their sodium intake by 24.7%, equivalent to ~0.6 teaspoons as a family.

We acknowledge the apparent incongruity regarding the absence of change in both BMI and waist circumference in the context of significant reductions in self-reported total energy intake over 6 months, a finding that is seemingly implausible. These results are in contrast to existing cross-sectional and longitudinal evidence on frequent consumption of avocado being associated with significantly lower BMI, body weight, and waist circumference compared to non-consumption [34,35]. Several explanations for the discrepancy must be considered, including possible measurement errors by study personnel, underreporting of dietary intake, failure to consider specific ethnic foods or beverages unaccounted for by the FFQ, and/or the inexactitude of the dietary intake tool. Notably, if the discrepancy was due to underreporting of energy generally, or of ethnicity-specific foods, our results suggest underreporting differed by intervention group, which is improbable. Regardless, our laboratory and anthropometric results suggest the provision of a higher allotment of avocados did not result in adverse changes in cardiometabolic risk factors. Several 24-h food recalls were considered in addition to the FFQs, and we acknowledge these may have provided better estimates of energy intake. However, the use of 24-h food recalls was not feasible since the majority of the study families did not have internet connection to be able to complete them at home and we did not have the necessary staff to go to their homes and collect that data. Additionally, families had complicated daily schedules that did not allow them to come into the clinic to complete these food recalls.

Although between-group differences in lipids did not reach statistical significance, there was a greater reduction in total cholesterol, VLDL lipoprotein cholesterol, and triglycerides in the high avocado allocation group, whereas there were greater mean reductions in LDL cholesterol in the low avocado allocation group. These findings contrast a recent meta-analysis by Peou et al., that examined the effect of diets enriched with avocado on plasma lipoproteins and found that avocado-enriched diets where dietary fats are substituted (vs. added) to free diets, significantly decreased total cholesterol, LDL, and triglyceride levels [36]. A possible explanation for these findings may be the design of the intervention; i.e., we supplemented existing diets and emphasized healthy dietary guidelines, without enforcing replacement of particular nutrients.

Our study had several strengths, including being a relatively long-term, large, cluster randomized controlled trial of families. The cultural appropriateness of the intervention design and engagement of experienced bicultural and bilingual educators, promotoras, in the home, are additional strengths. On the other hand, there are limitations. First, the use of self-reported data to evaluate dietary and nutrient intake changes could be a source of information bias. Second, we acknowledge incorporating an additional dietary data collection method would have strengthened the results, yet this was not feasible in our participants, as discussed above. Third, and related, social desirability bias, recall bias, and other potential reporting biases may have affected the findings. However, it seems reasonable to presume that these biases, if they existed, would be non-differential between groups because both intervention groups completed identical recall assessments and received identical advice on a healthful diet and adherence to MyPlate and DGA. Furthermore, while underreporting bias could be contributing to the inconsistency between the energy intake and anthropometric findings, some energy reduction still occurred since the study groups demonstrated separation by avocado intake level at both 3 and 6 months, with a greater effect between 3 and 6 months. Although we cannot rule out possible non-random error or be confident the FFQ provided an accurate assessment, an allotment of a higher number of avocados did not result in adverse changes in the different cardiometabolic outcome measures and risk factors. Fourth, we recognize our findings are limited to families of Hispanic/Latino heritage. Fifth, attrition was only observed in the low avocado group (16.2% dropout rate), which may have impacted statistical power in the primary analysis. In analyses where we removed those lost to follow-up and where we adjusted for intervention adherence, the primary results persisted.

## 5. Conclusions

This trial demonstrated that a differential allotment of avocados may impact overall self-reported caloric consumption, as well as macro- and micronutrient nutrient intake, including saturated fat and sodium, and food groups, including dairy, refined grains, and red and processed meats. Our trial results may help provide a strategy to support existing public health efforts to reduce saturated fat and sodium, which are commonly consumed in excess. It also demonstrates a high degree of adherence to the incorporation of a single, nutrient-dense plant food. These observations should be interpreted with caution since there were no statistically significant between-group differences in BMI, waist circumference, or cardiometabolic biomarkers. However, when combined with nutrition education, a higher level of avocados may be incorporated into a healthful diet for families who are of Hispanic/Latino heritage, seemingly without adverse cardiometabolic effects. Testing of a culturally appropriate plant-food on energy intake, by bicultural and bilingual community health workers, should be extended to other populations.

## Figures and Tables

**Table 1:** Baseline demographic and dietary characteristics of randomized families participating in the Effects of Avocado Intake on the Nutritional Status of Families Trial.

Characteristic	Low Avocado Allotment (*n* = 37 Families)		High Avocado Allotment (*n* = 35 Families)	
Family				
Total number of participants	118		113	
	N	%	N	%
Female				
Head of household ^1^	36	97.3	35	100
Non-head of household adult ^2^	13	35.1	15	46.9
Adolescent ^3^	9	69.2	13	72.2
Child ^4^	12	40.0	17	60.7
Family income in US less than $30,000 dollars/year	15	40.5	19	54.3
	Mean	SD	Mean	SD
Average family size	3	0.5	3	0.5
Age, years				
Head of household	46.5	11.3	44.5	8.4
Non-head of household adult	44.4	19.5	38.0	18.3
Adolescent	16.0	1.0	15.6	1.4
Child	9.0	2.1	9.4	2.2
Head of household				
	Mean	SD	Mean	SD
Years lived in the United States	16.5	12.6	17.7	12.9
	N	%	N	%
Country of birth, Mexico	32	86.5	28	80.0
Heritage, Mexican	36	97.3	34	97.1
Marital status				
Married or cohabitation	27	73.0	25	71.5
Separated, divorced, or widowed	7	18.9	7	20.1
Single	3	8.1	3	8.6
Highest level of education achieved				
High school	9	24.3	10	28.6
Trade school or Associate’s degree	9	24.3	11	31.4
Bachelor’s degree or above	8	21.6	8	22.9
No diploma	4	10.8	3	8.6
Other	7	18.9	3	8.6
Country where highest level of education was completed				
United States or other	11	29.7	16	45.8
Mexico	26	70.3	19	54.3
Employment status				
Employed for wages	9	24.3	10	28.6
Self-employed	4	10.8	3	8.6
Homemaker	18	48.7	14	40.0
Other	6	16.2	8	22.9
Family dietary intake				
Nutrients				
Total energy intake, kcal	6574.4	2287.9	7321.5	4406.0
Carbohydrate, % total energy	51.8	3.9	51.2	5.6
Protein, % total energy	16.0	1.7	16.5	2.4
Fat, % total energy	33.8	3.4	33.3	4.6
Carbohydrate, g	851.2	302.3	947.2	599.8
Dietary fiber, g	82.0	28.9	89.8	49.0
Protein, g	260.8	90.3	295.5	170.1
Animal origin, g	158.6	65.1	176.6	112.6
Vegetable origin, g	102.2	33.4	118.9	76.7
Fat, g	247.9	92.0	268.7	162.9
Saturated fat, g	80.4	32.7	84.1	55.2
Monounsaturated fat, g	94.9	36.1	103.3	62.9
Polyunsaturated fat, g	52.5	19.2	59.4	34.9
Calcium, mg	3781.6	1202.0	4116.5	2577.1
Magnesium, mg	1137.5	367.9	1281.9	683.1
Sodium, mg	13,017.5	5502.9	13,793.6	8245.0
Potassium, mg	10,159.2	3346.3	11,374.3	6104.7
Iron, mg	51.1	16.6	61.7	39.3
Vitamin C, mg	446.9	158.7	514.4	318.3
Vitamin D, mcg	23.3	9.0	25.5	18.1
Vitamin E, IU	53.9	21.3	60.9	41.5
Folate, mcg	858.1	288.4	967.6	551.1
HEI 2015 score, average range 0–300 (~3 members per household)	208.5	31.4	216.1	44.8
Head of household	70.9	8.7	71.0	6.7
Non-Head of Household	64.1	9.1	64.0	9.5
Adolescents	59.1	9.8	66.6	12.1
Children	63.1	9.4	65.5	11.5
Food Groups				
Fruit, cup equivalents	5.13	2.44	5.24	3.07
Vegetables, cup equivalents	5.41	2.32	6.38	3.87
Greens, cup equivalents	3.84	1.74	4.50	2.71
Legumes, cup equivalents	0.81	0.61	0.83	0.62
Dairy, cup equivalents	6.51	2.93	6.73	4.98
Nuts, ounce equivalents	1.59	1.37	2.05	3.29
Whole grains, ounce equivalents	4.97	3.01	5.41	3.62
Refined grains, ounce equivalents	19.55	9.10	22.37	16.45
Processed meat, ounce equivalents	1.85	1.14	2.35	2.40
Chicken and eggs, ounce equivalents	5.48	2.86	6.09	3.98
Fish, ounce equivalents	2.57	2.10	2.77	2.61
Beef, ounce equivalents	3.04	2.65	3.61	3.50
Sugar, teaspoon equivalents	35.34	20.89	37.92	32.67
Oils, g	76.42	32.69	82.16	49.66
Soymilk, cup equivalents	0.15	0.47	0.29	0.92
Soy, ounce equivalents	0.87	1.68	1.51	3.77

^1^ Head of household, *n* = 37 low allotment group and *n* = 35 high allotment group. ^2^ Non-head of household adult, *n* = 37 low allotment group and *n* = 32 high allotment group. ^3^ Adolescent, *n* = 14 low allotment group and *n* = 18 high allotment group. ^4^ Child, *n* = 30 low allotment and *n* = 28 high allotment group.

**Table 2:** Baseline anthropometric and clinical characteristics of randomized families participating in the Effects of Avocado Intake on the Nutritional Status of Families Trial.

Characteristic	Low Avocado Allotment (*n* = 37 Families)		High Avocado Allotment (*n* = 35 Families)	
	Mean	SD	Mean	SD
Body mass index, kg/m^2^				
Head of household ^1^	30.6	6.1	30.5	6.2
Non-head of household adult ^2^	31.7	6.2	28.6	6.9
Waist-to-height ratio, cm				
Adolescent ^3^	0.5	0.1	0.5	0.1
Child ^4^	0.5	0.1	0.5	0.1
Waist circumference, cm				
Head of household				
Female	93.2	13.4	95.0	12.7
Non-Head of household adult				
Female	102.7	15.2	84.3	14.1
Male	103.8	11.1	99.1	14.3
Adolescent				
Female	78.3	14.7	82.5	10.3
Male	73.4	13.3	81.5	14.7
Child				
Female	65.6	10.5	68.5	15.9
Male	66.2	13.4	67.5	7.8
Systolic blood pressure, mmHg				
Head of household	116.9	15.8	111.5	15.7
Non-head of household adult	122.6	16.0	122.4	21.2
Adolescent	108.9	8.2	109.7	8.2
Child	98.3	7.9	100.4	7.7
Diastolic blood pressure, mmHg				
Head of household	73.4	12.3	69.4	8.8
Non-head of household adult	71.8	10.3	73.2	9.3
Adolescent	62.7	9.8	65.9	6.3
Child	58.5	8.0	61.3	9.6
MVPA, minutes/week				
Head of household	593.9	672.9	582.9	649.8
Non-head of household adult	1200.4	1444.1	724.8	980.9
Adolescent	656.8	660.2	424.6	397.2
Child	240.2	304.1	428.8	625.3
Free fatty acids, mg/dL				
Head of household	0.5	0.2	0.4	0.2
Non-head of household adult ^5^	0.5	0.2	0.4	0.2
RBC magnesium, mg/dL				
Head of household ^1^	6.2	1.1	5.5	0.6
Non-head of household adult ^6^	5.6	0.4	5.3	0.5
Lipids, mg/dL				
Total cholesterol				
Head of household ^1^	189.2	40.8	188.7	32.9
Non-head of household adult ^5^	185.5	39.5	180.3	33.0
HDL cholesterol				
Head of household ^1^	52.7	15.0	49.9	10.8
Non-head of household adult ^5^	44.8	11.9	51.8	15.6
LDL cholesterol				
Head of household ^1^	111.4	37.4	112.1	27.1
Non-head of household adult ^7^	112.3	38.4	105.9	33.2
VLDL cholesterol				
Head of household ^1^	23.8	10.6	26.	14.9
Non-head of household adult ^7^	27.1	11.5	22.7	9.2
Triglycerides				
Head of household ^1^	138.5	130.5	142.8	95.8
Non-head of household adult ^5^	163.8	109.0	113.1	46.2
Glucose, mg/dL ^5^				
Head of household ^1^	102.3	38.3	103.5	30.4
Non-head of household adult	122.0	53.3	92.3	10.0
Insulin, microIU/mL (uIU/mL)				
Head of household ^1^	14.2	8.9	17.2	16.9
Non-head of household adult	15.7	8.8	15.1	10.4
HOMA-IR				
Head of household^1^	3.7	0.4	4.6	0.8
Non-head of household adult	5.1	0.8	3.4	0.6
Hemoglobin A1c% ^5^				
Head of household ^1^	5.9	1.2	5.7	1.0
Non-head of household adult	6.5	1.7	5.4	0.4
C-reactive protein, mg/L				
Head of household ^1^	3.9	4.5	2.9	2.8
Non-head of household adult ^5^	4.2	9.0	3.9	4.0

HDL, high-density lipoprotein; HOMA-IR, homeostatic model assessment of insulin resistance; LDL, low-density lipoprotein; MVPA, moderate-vigorous physical activity; RBC, red blood cell. ^1^ Head of household, *n* = 37 low allotment group and *n* = 35 high allotment group. ^2^ Non-head of household adult, *n* = 37 low allotment group and *n* = 32 high allotment group. ^3^ Adolescent, *n* = 14 low allotment group and *n* = 18 high allotment group. ^4^ Child, *n* = 30 low allotment and *n* = 28 high allotment group. ^5^ Adults only, *n* = 23 low allotment and *n* = 18 high allotment group. ^6^ Adults only, *n* = 7 low allotment and *n* = 10 high allotment group. ^7^ Adults only, *n* = 21 low allotment and *n* = 18 high allotment group.

**Table 3:** Changes in family nutritional status per intention-to-treat analysis in the Effects of Avocado Intake on the Nutritional Status of Families Trial (*n* = 72).

	Within-Group Differences				Between-Group Difference		*p*-Value ^1^
	Low Avocado Allotment		High Avocado Allotment				
	(*n* = 37)		(*n* = 35)				
	Mean	(95% CI)	Mean	(95% CI)	Mean	(95% CI)	
Total energy intake, kcal							
Difference at 3 months	−372.9	−918.0, 172.3	−1650.1	−2706.2, −593.9	1277.2	102.7, 2451.7	0.04
Difference at 6 months	−259.0	−958.0, 440.0	−2143.1	−3286.5, −999.8	1884.1	562.8, 3205.4	0.01
Carbohydrate, g							
Difference at 3 months	−90.6	−166.9, −14.2	−244.2	−388.0, −100.4	153.6	−3.8, 311.1	0.06
Difference at 3 months ^2^	−113.7	−196.7, −30.7	−219.7	−305.0, −134.3	106.0	−13.4, 225.4	0.08
Difference at 6 months	−64.8	−159.4, 29.7	−285.1	−431.9, −138.4	220.3	50.7, 389.9	0.01
Difference at 6 months ^2^	−91.0	−176.9, −5.0	−257.5	−345.9, −169.1	166.6	42.9, 290.2	0.01
Dietary fiber, g							
Difference at 3 months	−8.5	−15.2, −1.7	−6.8	−17.9, 4.4	−1.7	−14.4, 11.0	0.79
Difference at 3 months ^2^	−10.1	−17.3, −2.9	−5.0	−12.5, 2.4	−5.1	−15.5, 5.3	0.33
Difference at 6 months	−5.1	−14.2, 3.9	−14.3	−27.0, −1.6	9.2	−6.0, 24.3	0.23
Difference at 6 months ^2^	−7.0	−15.8, 1.7	−12.3	−21.3, 3.3	5.2	−7.4, 17.9	0.41
Protein, g							
Difference at 3 months	−11.3	−34.4, 11.8	−59.4	102.2, −16.5	48.1	0.9, 95.2	0.05
Difference at 3 months ^2^	−17.3	−44.4, 9.8	−53.0	−80.9, −25.1	35.7	−3.4, 74.7	0.07
Difference at 6 months	−1.2	−32.6, 30.2	−91.1	−134.5, −47.7	89.9	37.8, 142.1	0.001
Difference at 6 months ^2^	−8.0	−37.7, 21.8	−83.9	−114.6, −53.3	−6.0	−33.1, 118.8	0.001
Animal origin, g							
Difference at 3 months	−1.9	−18.1, 14.3	−36.1	−65.8, −6.4	34.2	1.5, 67.0	0.04
Difference at 3 months ^2^	−5.5	−25.5, 14.6	−32.3	−53.0, −11.7	26.8	−2.0, −55.7	0.07
Difference at 6 months	5.0	−15.9, 26.0	−56.3	−85.7, −26.8	61.3	−26.1, 96.5	0.001
Difference at 6 months ^2^	1.2	−20.4, 22.9	−52.3	−74.6, −30.0	53.5	22.3, 84.7	0.001
Vegetable origin, g							
Difference at 3 months	−9.4	−19.0, 0.2	−23.3	−40.3, −6.2	13.9	−5.1, 32.8	0.15
Difference at 3 months ^2^	−11.9	−22.7, −1.0	−20.7	−31.9, −9.5	8.8	−6.8, 24.5	0.26
Difference at 6 months	−6.2	−18.8, 6.4	−34.8	−53.5, −16.2	28.6	6.8, 50.5	0.01
Difference at 6 months ^2^	−9.2	−21.3, 2.9	−31.7	−44.1, −19.2	22.5	5.1, 39.9	0.01
Fat, g							
Difference at 3 months	−9.6	−32.3, 13.0	−44.0	−84.4, −3.7	34.4	−10.4, 79.2	0.13
Difference at 3 months ^2^	−16.0	−40.4, 8.5	−37.4	−62.6, −12.2	21.5	−13.7, 56.7	0.23
Difference at 6 months	−7.7	−34.2, 18.8	−66.0	−113.7, −18.3	58.3	5.5, 111.1	0.03
Difference at 6 months ^2^	−15.3	−43.6, 13.0	−58.0	−87.1, −28.9	42.7	1.9, 83.4	0.04
MUFA, g							
Difference at 3 months	−3.8	−12.4, 4.8	−9.7	−24.1, 4.8	5.8	−10.4, 22.1	0.48
Difference at 3 months ^2^	−6.0	−15.1, 3.0	−7.3	−16.6, 2.0	1.3	−11.7, 14.3	0.84
Difference at 6 months	−3.4	−13.7, 6.9	−17.8	−37.1, 1.5	14.4	−6.7, 35.5	0.18
Difference at 6 months ^2^	−6.4	−17.9, 5.2	−14.7	−26.6, −2.8	8.3	−8.3, 25.0	0.32
PUFA, g							
Difference at 3 months	−1.2	−6.6, 4.2	−12.6	−21.7, −3.5	11.4	1.2, 21.7	0.03
Difference at 3 months ^2^	−2.4	−8.5, 3.6	−11.3	−17.5, 5.1	8.8	0.2, 17.5	0.05
Difference at 6 months	−1.5	−7.3, 4.4	−17.1	−27.2, −7.1	15.7	4.4, 26.9	0.01
Difference at 6 months ^2^	−3.0	−9.2, 3.2	−15.5	−21.9, 9.1	12.5	3.5, 21.4	0.01
Saturated fat, g							
Difference at 3 months	−4.0	−12.3, 4.4	−19.0	−33.8, −4.2	15.0	−1.5, 31.5	0.07
Difference at 3 months ^2^	−6.3	−15.3, 2.7	−16.5	−25.8, −7.3	10.3	−2.7, 23.2	0.12
Difference at 6 months	−2.8	−12.3, 6.8	−26.5	−42.4, −10.7	23.8	5.8, 41.7	0.01
Difference at 6 months ^2^	−5.3	−15.1, 4.5	−23.9	−33.9, −13.8	18.6	4.5, 32.6	0.01
Calcium, mg							
Difference at 3 months	−259.7	−676.2, 156.9	−871.5	−1432.0, −310.9	611.8	−69.0, 1292.6	0.08
Difference at 3 months ^2^	−318.0	−760.4, 124.5	−809.8	−1264.8, −354.9	491.9	−144.6, 1128.3	0.13
Difference at 6 months	−265.2	−703.3, 172.9	−1302.0	−1904.2, −699.8	1036.8	311.2, −1762.4	0.01
Difference at 6 months ^2^	−353.1	−781.3, 75.1	−1209.1	−1649.4, −768.7	856.0	240.0, −1472.0	0.01
Magnesium, mg							
Difference at 3 months	−94.2	−180.9, −7.6	−197.6	−341.9, 53.4	103.4	−59.8, 266.6	0.21
Difference at 3 months ^2^	−115.3	−208.7, −21.9	−175.3	−271.4, −79.3	60.0	−74.4, 194.4	0.38
Difference at 6 months	−59.4	−163.0, 44.2	−339.5	−505.5, −173.5	280.2	90.2, 470.1	0.004
Difference at 6 months ^2^	−86.6	−188.8, 15.7	−310.8	−415.9, −205.7	224.2	77.2, 371.3	0.003
Sodium, mg							
Difference at 3 months	−1582.8	−3194.7, 29.2	−2436.8	−4460.0, −413.7	854.1	−1672.7, 3380.8	0.50
Difference at 3 months ^2^	−1919.2	−3342.8, −495.6	−2081.2	−3545.1, −617.2	161.9	−1886.0, 2209.9	0.88
Difference at 6 months	−737.8	−2618.0, 1142.4	−3402.0	−5340.0, −1464.1	2664.3	11.7, 5316.8	0.05
Difference at 6 months ^2^	−1105.2	−2564.6, 354.1	−3013.6	−4514.3, −1512.9	1908.4	−191.1, 4007.8	0.07
Potassium, mg							
Difference at 3 months	−678.8	−1476.3, 118.6	−1000.6	−2299.6, 298.4	321.8	−1156.9, 1800.5	0.67
Difference at 3 months ^2^	−848.8	−1739.7, 42.1	−820.9	−1737.0, 95.2	−27.9	−1309.5, 1253.7	0.97
Difference at 6 months	−324.3	−1291.1, 642.6	−2189.6	−3554.1, −825.1	1865.4	237.0, −3493.7	0.03
Difference at 6 months ^2^	−534.2	−1467.6, 399.3	−1967.7	−2927.6, −1007.8	1433.5	90.7, −2776.4	0.04
Iron, mg							
Difference at 3 months	−5.5	−10.9, 0.1	−16.9	−26.1, −7.7	11.4	1.1, 21.7	0.03
Difference at 3 months ^2^	−6.9	−12.6, −1.2	−15.4	−21.3, −9.6	8.5	0.3, 16.7	0.04
Difference at 6 months	−4.6	−10.9, 1.6	−21.6	−31.9, −11.4	17.0	5.4, 28.6	0.01
Difference at 6 months ^2^	−6.2	−12.7, 0.4	−20.0	−26.7, −13.2	13.8	4.4, 23.2	0.01
Vitamin C, mg							
Difference at 3 months	−36.2	−92.9, 20.5	−7.3	−92.6, 78.1	−29.0	−128.5, 70.6	0.56
Difference at 3 months ^2^	−42.0	−109.7, 25.6	−1.1	−70.6, 68.5	−41.0	−138.3, 56.3	0.40
Difference at 6 months	3.1	−76.4, 82.7	−46.9	−142.4, 48.5	−50.0	−172.2, 72.1	0.42
Difference at 6 months ^2^	−4.1	−86.6, 78.3	−39.3	−124.0, 45.6	35.1	−83.5, 153.7	0.56
Vitamin D, mcg							
Difference at 3 months	−0.6	−3.6, 2.5	−4.2	−9.6, 1.1	3.7	−2.3, 9.7	0.23
Difference at 3 months ^2^	−1.0	−5.0, 3.0	−3.8	−7.9, 0.3	2.8	−2.9, 8.6	0.33
Difference at 6 months	−1.1	−3.8, 1.5	−8.7	−12.6, −4.7	7.5	2.9, 12.2	0.002
Difference at 6 months ^2^	−1.6	−4.6, 1.4	−8.2	−11.3, 5.1	6.7	2.4, 11.0	0.003
Vitamin E, IU							
Difference at 3 months	−7.0	−13.7, −0.3	−5.2	−15.2, 4.7	−1.8	−13.4, 9.9	0.76
Difference at 3 months ^2^	−7.8	−15.6, 0.1	−4.4	−12.5, 3.6	−3.3	−14.6, 7.9	0.56
Difference at 6 months	−5.2	−12.2, 1.8	−13.6	−26.4, −0.9	8.4	−5.6, 22.5	0.24
Difference at 6 months ^2^	−6.4	−15.5, 2.7	−12.3	−21.7, 2.9	5.9	−7.2, 19.1	0.37
Folate, mcg							
Difference at 3 months	−59.1	−136.0, 17.7	−21.4	−140.9, 98.0	−37.7	−175.6, 100.2	0.59
Difference at 3 months ^2^	−74.3	−158.7, 10.0	−5.4	−92.1, 81.4	−68.9	−190.3, 52.4	0.26
Difference at 6 months	−41.0	−131.2, 49.2	−106.6	−250.8, 37.6	65.6	−99.4, 230.7	0.43
Difference at 6 months ^2^	−61.7	−157.6, 34.3	−84.7	−183.4, 13.9	23.1	−115.0, 161.1	0.74
HEI-2015 family score							
Difference at 3 months	4.8	−0.3, 9.9	7.5	0.4, 14.6	−2.7	−11.2, 5.8	0.53
Difference at 3 months ^2^	5.6	0.1, 11.1	6.7	1.0, 12.4	−1.1	−9.1, 6.8	0.77
Difference at 6 months	0.0	−6.4, 6.4	8.0	0.9, 15.1	−8.0	−17.4, 1.4	0.09
Difference at 6 months ^2^	0.4	−6.1, 6.9	7.6	0.9, 14.3	−7.2	−16.6, 2.1	0.13

Mean difference variable is low minus (−) high avocado allotment group. HEI-2015, healthy eating index 2015; MUFA, monounsaturated fatty acids; PUFA, polyunsaturated fatty acids. ^1^ From unpaired *t*-test or ANCOVA model (adjusted for baseline total energy intake), where appropriate. ^2^ Adjusted for baseline total energy intake.

**Table 4:** Changes in family food group composition per intention-to-treat analysis in the Effects of Avocado Intake on the Nutritional Status of Families Trial (*n* = 72).

	Within-Group Differences				Between-Group Difference		*p*-Value ^1^
	Low Avocado Allotment		High Avocado Allotment				
	(*n* = 37)		(*n* = 35)				
	Mean	95% CI	Mean	95% CI	Mean	95% CI	
Fruit, cup equivalents							
Difference at 3 months	−0.3	−0.8, 0.3	0.7	−0.2, 1.6	−1.0	−2.0, 0.1	0.07
Difference at 3 months ^2^	−0.3	−1.0, 0.5	0.7	−0.0, 1.5	−1.0	−2.1, 0.0	0.06
Difference at 6 months	−0.3	−1.0, 0.4	0.3	−0.6, 1.2	−0.6	−1.7, 0.6	0.32
Difference at 6 months ^2^	−0.4	−1.1, 0.4	0.4	−0.4, 1.1	−0.7	−1.8, 0.4	0.20
Vegetables, cup equivalents							
Difference at 3 months	−0.2	−0.8, 0.5	0.2	−0.6, 1.0	−0.4	−1.4, 0.7	0.49
Difference at 3 months ^2^	−0.2	−0.9, 0.5	0.2	−0.5, 1.0	−0.5	−1.5, 0.5	0.37
Difference at 6 months	−0.1	−0.9, 0.7	−0.4	−1.5, 0.6	0.3	−0.9, 1.6	0.61
Difference at 6 months ^2^	−0.2	−1.0, 0.7	−0.4	−1.2, 0.5	0.2	−1.1, 1.4	0.77
Greens, cup equivalents							
Difference at 3 months	−0.1	−0.5, 0.4	0.2	−0.5, 0.9	−0.2	−1.1, 0.6	0.57
Difference at 3 months ^2^	−0.1	−0.7, 0.5	0.2	−0.4, 0.8	−0.3	−1.1, 0.5	0.47
Difference at 6 months	0.0	−0.6, 0.6	−0.3	−1.0, 0.5	0.3	−0.7, 1.2	0.55
Difference at 6 months ^2^	−0.0	−0.7, 0.6	−0.2	−0.9, 0.5	0.2	−0.7, 1.1	0.68
Legumes, cup equivalents							
Difference at 3 months	−0.2	−0.4, −0.1	0.2	−0.4, 0.0	0.0	−0.3, 0.2	0.92
Difference at 3 months ^2^	−0.2	−0.4, −0.0	−0.2	−0.4, 0.0	0.0	−0.3, 0.2	0.77
Difference at 6 months	−0.1	−0.3, 0.1	−0.3	−0.5, −0.1	0.2	−0.1, 0.5	0.20
Difference at 6 months ^2^	−0.1	−0.3, 0.1	−0.3	−0.5, −0.1	0.2	−0.1, 0.4	0.29
Dairy, cup equivalents							
Difference at 3 months	−0.3	−1.2, 0.6	−1.4	−2.7, −0.2	1.1	−0.4, 2.7	0.15
Difference at 3 months ^2^	−0.4	−1.4, 0.7	−1.3	−2.4, −0.3	1.0	−0.5, 2.5	0.21
Difference at 6 months	−0.3	−1.3, 0.7	−2.3	−3.5, −1.1	2.0	0.5, 3.5	0.01
Difference at 6 months ^2^	−0.4	−1.4, 0.6	−2.2	−3.2, −1.2	1.8	0.3, 3.2	0.02
Nuts, ounce equivalents							
Difference at 3 months	−0.3	−0.8, 0.1	−0.5	−1.3, 0.4	0.1	−0.8, 1.1	0.78
Difference at 3 months ^2^	−0.3	−0.9, 0.4	−0.5	−1.2, 0.2	0.2	−0.7, 1.1	0.64
Difference at 6 months	−0.3	−0.9, 0.3	−1.0	−2.1, 0.1	0.7	−0.5, 1.9	0.26
Difference at 6 months ^2^	−0.4	−1.2, 0.4	−0.9	−1.7, −0.1	0.5	−0.6, 1.7	0.37
Whole grains, ounce equivalents							
Difference at 3 months	−0.2	−1.1, 0.7	−1.5	−2.2, −0.7	1.3	0.1, 2.5	0.04
Difference at 3 months ^2^	−0.3	−1.1, 0.5	−1.4	−2.1, 0.6	1.0	−0.1, 2.1	0.06
Difference at 6 months	−0.6	−1.6, 0.5	−1.9	−3.1, −0.7	1.3	−0.2, 2.9	0.09
Difference at 6 months ^2^	−0.7	−1.7, 0.3	−1.7	−2.8, 0.7	1.0	−0.4, 2.5	0.15
Refined grains, ounce equivalents							
Difference at 3 months	−2.4	−4.9, 0.1	−8.5	−13.4, −3.7	6.1	0.7, 11.5	0.03
Difference at 3 months ^2^	−3.1	−6.0, −0.2	−7.7	−10.7, −4.7	4.6	0.4, 8.8	0.03
Difference at 6 months	−0.6	−4.4, 3.2	−9.2	−13.2, −5.2	8.5	3.1, 13.9	0.002
Difference at 6 months ^2^	−1.4	4.4, 1.6	−8.4	−11.5, −5.3	7.0	2.7, 11.3	0.002
Processed meats, ounce equivalents							
Difference at 3 months	−0.0	−0.4, 0.4	−0.7	−1.4, 0.0	0.6	−0.2, 1.4	0.11
Difference at 3 months ^2^	−0.1	−0.6, 0.4	−0.6	−1.1, −0.1	0.5	−0.2, 1.2	0.18
Difference at 6 months	0.1	−0.4, 0.6	−0.7	−1.6, 0.1	0.9	−0.1, 1.8	0.07
Difference at 6 months ^2^	0.1	−0.6, 0.7	−0.7	−1.3, −0.0	0.7	−0.2, 1.6	0.11
Chicken and eggs, ounce equivalents							
Difference at 3 months	−0.2	−0.9, 0.4	−1.4	−2.5, 0.3	1.2	−0.1, 2.5	0.06
Difference at 3 months ^2^	−0.3	−1.1, 0.5	−1.3	−2.2, −0.5	1.0	−0.2, 2.2	0.09
Difference at 6 months	0.8	−0.5, 2.1	−1.6	−2.9, −0.3	2.4	0.6, 4.2	0.01
Difference at 6 months ^2^	0.7	−0.6, 1.9	−1.5	−2.8, −0.2	2.2	0.4, 4.0	0.02
Fish, ounce equivalents							
Difference at 3 months	0.4	−0.5, 1.4	−0.2	−0.4, −2.6	0.6	−0.6, 1.9	0.32
Difference at 3 months ^2^	0.4	−0.5, 1.2	−0.1	−1.0, 0.8	0.5	−0.8, 1.7	0.47
Difference at 6 months	0.3	−0.3, 0.9	−0.5	−1.3, 0.2	0.8	−0.1, 1.8	0.09
Difference at 6 months ^2^	0.3	−0.4, 0.9	−0.5	−1.2, 0.2	0.7	−0.2, 1.7	0.13
Red meat, ounce equivalents							
Difference at 3 months	−0.0	−0.6, 0.6	−0.6	−1.9, 0.7	0.6	−0.8, 2.0	0.38
Difference at 3 months ^2^	−0.1	−1.0, 0.7	−0.5	−1.4, 0.4	0.4	−0.9, 1.6	0.58
Difference at 6 months	−0.1	−0.6, 0.5	−1.5	−2.6, −0.5	1.4	0.3, 2.6	0.02
Difference at 6 months ^2^	−0.2	−0.9, 0.5	−1.4	−2.1, −0.7	1.2	−0.2, 2.2	0.02
Sugar, teaspoon equivalents							
Difference at 3 months	−4.7	−9.7, 0.4	−14.5	−24.5, −4.6	9.9	−1.2, 20.9	0.08
Difference at 3 months ^2^	−6.0	−12.4, 0.4	−13.2	−19.7, −6.6	7.2	−2.0, 16.4	0.13
Difference at 6 months	−5.0	−9.4, −0.6	−12.0	−21.6, −2.4	7.0	−3.5, 17.5	0.19
Difference at 6 months ^2^	−6.4	−12.1, −0.6	−10.6	−16.5, −4.7	4.2	−4.1, 12.5	0.31
Oils, g							
Difference at 3 months	−0.8	−9.6, 8.1	−16.4	−30.0, −2.9	15.7	−0.3, 31.6	0.05
Difference at 3 months ^2^	−1.9	−12.4, 8.6	−15.2	−26.0, −4.5	13.3	−1.7, 28.4	0.09
Difference at 6 months	−7.8	−16.8, 1.3	−22.8	−38.8, −6.7	15.0	−3.2, 33.2	0.10
Difference at 6 months ^2^	−10.0	−20.5, 0.6	−20.5	−31.3, −9.7	10.5	−4.6, 25.6	0.17
Soymilk, cup equivalents							
Difference at 3 months	0.1	−0.1, 0.3	0.2	0.1, 0.4	−0.1	−0.4, 0.1	0.34
Difference at 3 months ^2^	0.1	−0.1, 0.3	0.2	0.0, 0.4	−0.1	−0.4, 0.2	0.39
Difference at 6 months	−0.1	−0.3, 0.1	−0.0	−0.3, 0.3	−0.1	−0.4, 0.3	0.77
Difference at 6 months ^2^	−0.1	−0.3, 0.2	−0.0	−0.4, 0.2	−0.1	−0.4, 0.3	0.76
Soy, ounce equivalents							
Difference at 3 months	0.3	−0.2, 0.7	0.4	−0.0, 0.8	−0.1	−0.7, 0.5	0.67
Difference at 3 months ^2^	0.3	−0.2, 0.7	0.4	−0.1, 0.8	−0.1	−0.7, 0.5	0.68
Difference at 6 months	−0.1	−0.6, 0.4	−0.3	−1.0, 0.4	0.2	−0.7, 1.0	0.67
Difference at 6 months ^2^	−0.1	−0.7, 0.5	−0.3	−0.9, 0.3	0.2	−0.7, 1.0	0.69

Mean difference variable is low minus (−) high avocado allotment group. ^1^ From unpaired *t*-test or ANCOVA model (adjusted for baseline total energy intake), where appropriate. ^2^ Adjusted for baseline total energy intake.

**Table 5:** Changes in family members’ body mass index, waist circumference, and blood pressure, per intention-to-treat analysis in the Effects of Avocado Intake on the Nutritional Status of Families Trial (*n* = 72 families).

	Within-Group Difference				Between-Group Difference		*p*-Value ^1^
	Low Avocado Allotment (*n* = 37 Families)		High Avocado Allotment (*n* = 35 Families)				
	Mean	95% CI	Mean	95% CI	Mean	95% CI	
Body mass index, kg/m^2^							
All adults ^2^							
Difference at 3 months	0.3	−0.2, 0.8	−0.1	−0.7, 0.4	−0.4	−1.2, 0.3	0.27
Difference at 6 months	−0.1	−0.4, 0.2	−0.01	−0.7, 0.7	0.1	−0.7, 0.9	0.77
Head of household ^3^							
Difference at 3 months	−0.1	−0.4, 0.3	−0.1	−0.6, 0.3	−0.1	−0.5, 0.6	0.79
Difference at 6 months	−0.4	−0.9, 0.1	0.1	−0.9, 1.1	−0.5	−1.6, 0.6	0.37
Non-head of household adult ^4^							
Difference at 3 months	0.7	−0.3, 1.6	−0.1	−1.2, 1.0	0.8	−0.7, 2.2	0.29
Difference at 6 months	0.1	−0.3, 0.5	−0.2	−1.2, 0.9	0.3	−0.9, 1.4	0.62
Waist-to-height ratio, cm							
Adolescent ^5^							
Difference at 3 months	0.01	−0.01, 0.02	0.01	−0.01, 0.03	0.003	−0.03, 0.02	0.83
Difference at 6 months	0.01	−0.01, 0.03	0	−0.02, 0.02	0.01	−0.02, 0.04	0.46
Child ^6^							
Difference at 3 months	0	−0.01, 0.01	0.01	−0.01, 0.03	−0.01	−0.03, 0.01	0.30
Difference at 6 months	0.01	−0.01, 0.02	0.01	−0.003, 0.03	−0.004	−0.02, 0.01	0.68
Waist circumference, cm							
All adults ^2^							
Female							
Difference at 3 months	0.8	−1.1, 2.7	0.7	−1.3, 2.8	−0.05	−2.8, 2.7	0.97
Difference at 6 months	0.6	−1.4, 2.5	1.2	−0.5, 2.9	0.7	−1.9, 3.2	0.60
Male							
Difference at 3 months	−0.3	−2.3, 1.6	2.0	−0.02, 4.1	2.4	−0.5, 5.2	0.10
Difference at 6 months	1.7	−0.6, 3.9	2.7	−0.6, 6.0	1.0	−2.7, 4.8	0.57
Head of household ^3^							
Difference at 3 months	1.3	−0.8, 3.5	1.1	−1.2, 3.4	0.2	−2.9, 3.3	0.89
Difference at 6 months	1.3	−0.7, 3.4	1.0	−1.1, 3.2	0.3	−2.6, 3.2	0.85
Non-head of household adult ^4^							
Female							
Difference at 3 months	−0.7	−5.2, 3.7	−0.1	−4.7, 4.4	−0.6	−6.7, 5.5	0.85
Difference at 6 months	−1.7	−6.8, 3.3	1.5	−1.7, 4.8	−3.3	−8.8, 2.3	0.24
Male							
Difference at 3 months	−0.3	−2.3, 1.6	2.0	−0.02, 4.1	2.3	−5.2, 0.5	0.10
Difference at 6 months	1.7	−0.6, 3.9	2.7	−0.6, 6.0	−1.0	−4.8, 2.7	0.57
Adolescent ^5^							
Female							
Difference at 3 months	0.9	−2.1, 4.0	0.5	−4.0, 5.1	−0.4	−6.1, 5.3	0.88
Difference at 6 months	1.7	−3.0, 6.5	−0.5	−4.7, 3.6	−2.3	−8.2, 3.7	0.44
Male							
Difference at 3 months	0.6	−2.9, 4.1	4.1	−2.5, 10.7	3.5	−2.7, 9.7	0.23
Difference at 6 months	2.8	−0.9, 6.5	2.3	−3.3, 7.9	−0.5	−6.0, 5.0	0.84
Child ^6^							
Girl							
Difference at 3 months	−0.5	−3.6, 2.5	2.8	−0.2, 5.8	3.3	−0.9, 7.5	0.12
Difference at 6 months	0.3	−3.4, 4.1	3.2	0.7, 5.6	2.8	−1.2, 6.9	0.16
Boy							
Difference at 3 months	1.6	−0.1, 3.3	1.4	−2.2, 4.9	−0.2	−3.5, 3.1	0.89
Difference at 6 months	3.3	1.3, 5.4	3.2	−0.6, 7.0	−0.2	−3.9, 3.6	0.93
Systolic blood pressure, mmHg							
All adults ^2^							
Difference at 3 months	−2.7	−5.7, 0.3	−1.1	−4.0, 1.7	1.5	−2.6, 5.7	0.46
Difference at 6 months	−5.5	−8.5, −2.6	0.3	−2.3, 3.0	5.9	1.9, 9.8	0.004
Head of household ^3^							
Difference at 3 months	−2.6	−7.0, 1.7	−2.1	−6.2, 1.9	−0.5	−6.4, 5.4	0.87
Difference at 6 months	−4.8	−9.1, −0.5	0.8	−3.2, 4.9	−5.6	11.5, 0.2	0.06
Non-head of household adult ^4^							
Difference at 3 months	−2.7	−7.0, 1.6	−0.03	−4.3, 4.2	−2.7	−8.6, 3.3	0.37
Difference at 6 months	−6.2	−10.4, 2.1	−0.2	−3.9, 3.4	−6.0	−11.5, −0.5	0.03
Adolescent ^5^							
Difference at 3 months	−1.7	−5.3, 1.9	−1.4	−5.1, 2.3	0.3	−4.8, 5.4	0.90
Difference at 6 months	−2.1	−5.7, 1.6	−0.9	−4.7, 2.8	1.1	−4.0, 6.3	0.66
Child ^6^							
Difference at 3 months	−1.3	−4.6, 2.1	0.4	−2.0, 2.9	1.7	−2.3, 5.7	0.40
Difference at 6 months	−0.1	−3.2, 3.0	2.6	−0.8, 6.1	2.7	−1.8, 7.3	0.23
Diastolic blood pressure, mmHg							
All adults ^2^							
Difference at 3 months	−0.9	−3.0, 1.3	−0.8	−2.7, 1.1	0.1	−2.8, 2.9	0.96
Difference at 6 months	−1.8	−3.8, 0.2	−0.2	−2.0, 1.6	1.6	−1.1, 4.3	0.24
Head of household ^3^							
Difference at 3 months	−2.4	−5.2, 0.4	−1.2	−3.8, 1.4	−1.2	−5.0, 2.6	0.54
Difference at 6 months	−3.5	−6.3, −0.6	0.7	−2.4, 3.8	−4.2	−8.4, −0.1	0.05
Non-head of household adult ^4^							
Difference at 3 months	0.7	−2.6, 4.0	−0.3	−3.2, 2.6	1.0	−3.4, 5.4	0.65
Difference at 6 months	−0.1	−2.8, 2.6	−1.2	−3.1, 0.7	1.1	−2.2, 4.3	0.51
Adolescent ^5^							
Difference at 3 months	0.7	−2.4, 3.9	−2.1	−5.5, 1.4	−2.8	−7.4, 1.8	0.23
Difference at 6 months	0.8	−3.1, 4.7	−0.8	−4.7, 3.1	−1.6	−7.0, 3.8	0.54
Child ^6^							
Difference at 3 months	−2.9	−6.1, 0.4	−2.1	−5.3, 1.1	0.8	−3.7, 5.3	0.73
Difference at 6 months	−2.9	−5.3, −0.6	−0.8	−5.0, 3.5	2.2	−2.6, 6.9	0.37

Mean difference variable is low minus (−) high avocado allotment group. ^1^ From unpaired *t*-test. ^2^ All adults, *n* = 74 in low allotment group and *n* = 67 in high allotment group, at baseline. ^3^ Heads of households, *n* = 37 in low allotment group and *n* = 35 in high allotment group, at baseline. ^4^ Non-head of household adults, *n* = 37 in low allotment group and *n* = 32 in high allotment group, at baseline. ^5^ Adolescent, *n* = 14 in low allotment group and *n* = 18 in high allotment group, at baseline. ^6^ Children, *n* = 30 in low allotment and *n* = 28 in high allotment group; with the exception of blood pressure where *n* = 28 in low allotment group and *n* = 27 in high allotment group, at baseline.

**Table 6:** Changes in free fatty acids, magnesium, lipids, glycemia markers, and c-reactive protein in adults, per intention-to-treat analysis in the Effects of Avocado Intake on the Nutritional Status of Families Trial (*n* = 113).

	Within-Group Difference				Between-Group Difference		*p*-Value ^1^
	Low Avocado Allotment (*n* = 60)		High Avocado Allotment (*n* = 53)				
	Mean	95% CI	Mean	95% CI	Mean	95% CI	
Free fatty acids, mg/dL							
All adults ^2^							
Difference at 3 months	−0.02	−0.05, 0.02	−0.002	−0.03, 0.03	0.01	−0.03, 0.06	0.56
Difference at 6 months	−0.02	−0.06, 0.02	0.01	−0.05, 0.07	0.03	−0.04, 0.10	0.42
Head of household ^3^							
Difference at 3 months	0.01	−0.04, 0.05	0	−0.03, 0.03	0.01	−0.04, 0.06	0.83
Difference at 6 months	−0.04	−0.09, 0.02	0.01	−0.07, 0.09	−0.04	−0.14, 0.05	0.37
Non-head of household adult ^4^							
Difference at 3 months	−0.05	0.10, −0.01	−0.01	−0.07, 0.06	−0.04	−0.13, 0.04	0.33
Difference at 6 months	0.004	−0.06, 0.07	0.01	0.08, 0.10	−0.01	−0.12, 0.10	0.90
Red blood cell magnesium ^5^, mg/dL							
All adults ^2^							
Difference at 3 months	−0.26	−0.41, −0.11	0.07	−0.13, 0.28	0.33	0.08, 0.59	0.01
Difference at 6 months	−0.27	−0.49, −0.06	0.08	−0.10, 0.26	0.36	0.09, 0.62	0.01
Head of household ^3^							
Difference at 3 months	−0.36	−0.59, −0.13	−0.03	−0.37, 0.32	−0.34	−0.71, 0.04	0.08
Difference at 6 months	−0.40	−0.78, −0.02	0.06	−0.27, 0.40	−0.46	−0.92, −0.0005	0.05
Non-head of household adult ^4^							
Difference at 3 months	−0.14	−0.37, 0.09	0.15	−0.15, 0.45	−0.29	−0.67, 0.09	0.12
Difference at 6 months	−0.13	−0.34, 0.08	0.10	−0.15, 0.35	−0.23	−0.55, 0.09	0.15
Lipids, mg/dL							
total cholesterol							
All adults ^2^							
Difference at 3 months	−1.6	−8.5, 5.2	−3.6	−11.3, 4.1	−2.0	−12.1, 8.2	0.70
Difference at 6 months	−7.5	−14.7, −0.3	−9.0	−16.1, −1.8	−1.5	−11.6, 8.6	0.77
Head of household ^3^							
Difference at 3 months	−1.2	−11.1, 8.8	−3.5	−14.3, 7.3	2.4	−12.0, 16.7	0.75
Difference at 6 months	−9.6	−19.3, 0.1	−6.3	−15.9, 3.4	−3.4	−16.8, 10.1	0.62
Non-head of household adult ^4^							
Difference at 3 months	−2.4	−11.4, 6.6	−3.7	−4.9, 20.6	1.3	−11.9, 14.5	0.84
Difference at 6 months	−4.0	−15.2, 7.2	−14.2	−24.6, −3.8	10.2	−4.9, 25.4	0.18
HDL cholesterol							
All adults ^2^							
Difference at 3 months	0.9	−1.0, 2.7	−1.5	−3.4, 0.5	−2.3	−5.0, 0.4	0.09
Difference at 6 months	−0.3	−2.0, 1.4	−2.3	−4.0, −0.5	−1.9	−4.4, 0.5	0.12
Head of household ^3^							
Difference at 3 months	−0.1	−2.2, 2.1	−1.2	−3.8, 1.4	1.1	−2.2, 4.4	0.50
Difference at 6 months	−1.2	−3.4, 1.1	−1.1	−2.7, 0.6	0.1	−2.9, 2.7	0.94
Non-head of household adult ^4^							
Difference at 3 months	2.3	−1.3, 5.9	−2.0	−4.9, 0.9	4.3	−0.4, 9.0	0.07
Difference at 6 months	1.0	−1.8, 3.8	−4.6	−8.7, −0.5	5.6	0.9, 10.3	0.02
LDL cholesterol ^6^							
All adults ^2^							
Difference at 3 months	−1.5	−7.4, 4.4	−2.9	−9.2, 3.4	−1.5	−10.0, 7.0	0.73
Difference at 6 months	−8.0	−14.5, −1.4	−6.3	−11.7, −1.0	1.7	−6.8, 10.1	0.70
Head of household ^3^							
Difference at 3 months	−1.8	−11.0, 7.5	−3.2	−11.4, 5.0	1.5	−10.8, 13.7	0.81
Difference at 6 months	−9.9	−18.9, −0.8	−5.2	−12.1, 1.6	−4.7	−15.9, 6.6	0.41
Non-head of household adult ^4^							
Difference at 3 months	−1.0	−4.6, 2.6	−2.4	−13.1, 8.2	1.4	−9.6, 12.5	0.79
Difference at 6 months	−4.8	−14.3, 4.8	−8.4	−17.9, 1.0	3.7	−9.4, 16.7	0.57
Very low LDL cholesterol ^6^							
All adults ^2^							
Difference at 3 months	0.2	−1.9, 2.3	0.7	−2.7, 4.1	0.5	−3.4, 4.5	0.80
Difference at 6 months	1.0	−1.1, 3.1	−0.1	−3.7, 3.5	−1.1	−5.2, 3.0	0.60
Head of household ^3^							
Difference at 3 months	0.7	−1.5, 2.9	0.7	−4.0, 5.4	−0.01	−5.2, 5.2	0.99
Difference at 6 months	1.2	−1.8, 4.1	0.5	−4.7, 5.7	0.7	−5.2, 6.6	0.81
Non-head of household adult ^4^							
Difference at 3 months	−0.7	−5.3, 4.0	0.7	−3.8, 5.3	−1.4	−7.7, 4.9	0.66
Difference at 6 months	0.7	−2.2, 3.7	−1.2	−5.1, 2.7	1.9	−2.8, 6.5	0.42
Triglycerides							
All adults ^2^							
Difference at 3 months	3.4	−16.7, 23.4	3.1	−13.6, 19.8	−0.2	−26.5, 26.0	0.99
Difference at 6 months	1.2	−11.1, 13.6	−3.0	−25.1, 19.2	−4.2	−29.4, 21.0	0.74
Head of household ^3^							
Difference at 3 months	9.7	−7.1, 26.5	2.5	−20.7, 25.6	7.2	−20.6, 35.1	0.61
Difference at 6 months	5.5	−8.8, 19.9	−1.5	−34.3, 31.2	7.1	−28.3, 42.4	0.69
Non-head of household adult ^4^							
Difference at 3 months	−6.8	−53.9, 40.3	4.4	−18.7, 27.5	−11.2	−62.6, 40.2	0.66
Difference at 6 months	−5.7	−29.6, 18.2	−5.7	−25.6, 14.1	0.03	−31.3, 31.3	0.99
Glucose, mg/dL							
All adults ^2^							
Difference at 3 months	−4.0	−12.3, 4.4	−3.9	−7.2, −0.5	0.1	−8.8, 9.0	0.98
Difference at 6 months	−3.6	−9.9, 2.8	−3.7	−7.8, 0.4	−0.1	−7.6, 7.4	0.97
Head of household ^3^							
Difference at 3 months	0.5	−10.9, 11.9	−6.8	−11.1, −2.5	7.3	−4.8, 19.5	0.23
Difference at 6 months	−3.3	−11.7, 5.1	−5.5	−11.4, 0.5	2.2	−8.0, 12.3	0.67
Non-head of household adult ^4^							
Difference at 3 months	−11.2	−23.4, 0.8	1.8	−2.5, 6.2	−13.1	−25.7, 0.4	0.04
Difference at 6 months	−4.0	−14.5, 6.5	−0.2	−3.9, 3.5	−3.8	−14.8, 7.2	0.49
Insulin, microIU/mL (uIU/mL)							
All adults ^2^							
Difference at 3 months	1.6	−1.3, 4.4	1.4	−4.1, 6.8	−0.2	−5.9, 6.3	0.95
Difference at 6 months	1.0	−0.7, 2.6	1.5	−3.4, 6.3	0.5	−4.6, 5.5	0.85
Head of household ^3^							
Difference at 3 months	2.2	−2.2, 6.6	0.2	−7.4, 7.8	2.0	−6.7, 10.7	0.64
Difference at 6 months	1.0	−1.0, 2.9	−0.6	−4.6, 3.5	1.5	−2.9, 6.0	0.49
Non-head of household adult ^4^							
Difference at 3 months	0.5	−2.0, 2.9	3.7	−3.3, 10.6	−3.2	−10.5, 4.1	0.37
Difference at 6 months	1.0	−2.1, 4.1	5.4	−7.1, 17.9	−4.4	−17.2, 8.4	0.48
HOMA-IR							
All adults ^2^							
Difference at 3 months	0.2	−0.6, 1.0	0.2	−1.2, 1.6	0.05	−1.6, 1.6	0.95
Difference at 6 months	0.1	−0.4, 0.5	0.4	−1.0, 1.7	0.3	−1.1, 1.7	0.64
Head of household ^3^							
Difference at 3 months	0.6	−0.6, 1.8	−0.2	−2.1, 1.8	0.7	−1.5, 3.0	0.52
Difference at 6 months	0.2	−0.3, 0.6	−0.2	−1.4, 0.9	0.4	−0.8, 1.6	0.49
Non-head of household adult ^4^							
Difference at 3 months	−0.5	−1.4, 0.4	0.9	−0.7, 2.6	−1.4	−3.3, 0.4	0.12
Difference at 6 months	−0.1	−1.1, 0.8	1.6	−1.9, 5.1	−1.7	−5.3, 1.9	0.33
Hemoglobin A1c, %							
All adults ^2^							
Difference at 3 months	0.003	−0.12, 0.13	−0.01	−0.08, 0.05	−0.02	−0.16, 0.12	0.81
Difference at 6 months	−0.03	−0.17, 0.10	−0.06	−0.14, 0.01	−0.03	−0.18, 0.12	0.67
Head of household ^3^							
Difference at 3 months	0.07	−0.11, 0.25	−0.02	−0.09, 0.05	0.09	−0.10, 0.29	0.34
Difference at 6 months	0	−0.18, 0.18	−0.09	−0.19, 0.01	0.09	−0.12, 0.30	0.40
Non-head of household adult ^4^							
Difference at 3 months	−0.11	−0.26, 0.04	0	−0.15, 0.15	−0.11	−0.32, 0.10	0.30
Difference at 6 months	−0.08	−0.29, 0.12	−0.02	−0.10, 0.07	−0.07	−0.28, 0.15	0.54
C-reactive protein, mg/L							
All adults ^2^							
Difference at 3 months	−0.5	−1.6, 0.7	−0.2	−0.6, 0.3	0.3	−0.9, 1.6	0.58
Difference at 6 months	−0.5	−1.5, 0.6	−0.4	−0.9, 0.1	0.1	−1.1, 1.2	0.88
Head of household ^3^							
Difference at 3 months	−0.4	−1.1, 0.3	0.1	−0.5, 0.6	−0.5	−1.4, 0.5	0.32
Difference at 6 months	−0.2	−0.9, 0.5	−0.2	−0.6, 0.3	−0.02	−0.9, 0.8	0.95
Non-head of household adult ^4^							
Difference at 3 months	−0.7	−3.5, 2.2	−0.6	−1.5, 0.3	−0.1	−3.0, 2.9	0.97
Difference at 6 months	−0.9	−3.5, 1.7	−0.8	−1.9, 0.2	−0.1	−2.9, 2.7	0.94

Mean difference variable is low minus (−) high avocado allotment group. HDL, high-density lipoprotein; HOMA-IR, homeostatic model assessment of insulin resistance; LDL, low-density lipoprotein. ^1^ From unpaired *t*-test. ^2^ Low allotment group *n* = 60, high allotment group *n* = 53, at baseline. ^3^ Low allotment group *n* = 37, high allotment group *n* = 35, at baseline. ^4^ Low allotment group *n* = 23, high allotment group *n* = 18, at baseline. ^5^ Low allotment group *n* = 15 (*n* = 8 for head of household and *n* = 7 for non-head of household adult), high allotment group *n* = 18 (*n* = 8 for head of household and *n* = 10 for non-head of household adult), at baseline. ^6^ Low allotment group *n* = 57 (*n* = 36 head of household and *n* = 21 non-head of household adult), high allotment group *n* = 52 (*n* = 34 for head of household and *n* = 18 for non-head of household adult), at baseline.

**Table 7:** Changes in family nutritional status per protocol adherence analysis in the Effects of Avocado Intake on the Nutritional Status of Families Trial.

	Within-Group Difference				Between-Group Difference		*p*-Value ^2^
	Low Avocado Allotment ^1^		High Avocado Allotment ^1^				
	Mean	95% CI	Mean	95% CI	Mean	95% CI	
Total energy intake, kcal							
Difference at 3 months	−267.1	−1132.8, 598.6	−1784.8	−2637.6, −932.1	−1517.7	−2759.3, −276.2	0.02
Difference at 6 months	34.0	−980.0, 1048.1	−2209.8	−3163.0, −1256.4	−2243.8	−3647.6, −839.9	0.002
Carbohydrate, g							
Difference at 3 months	−83.1	−202.3, 36.2	−259.2	−376.7, −141.8	−176.2	−347.2, −5.2	0.04
Difference at 3 months ^3^	−128.2	−217.7, −38.7	−215.4	−303.5, −127.2	−87.1	−216.6, 42.3	0.18
Difference at 6 months	−35.3	−168.2, 97.6	−293.8	−418.8, −168.8	−258.5	−442.6, −74.4	0.01
Difference at 6 months ^3^	−78.5	−173.0, 16.0	−255.6	−344.3, −166.8	−177.1	−308.6, −45.6	0.01
Dietary fiber, g							
Difference at 3 months	−8.7	−18.3, 1.0	−7.3	−16.8, 2.2	1.4	−12.5, 15.2	0.84
Difference at 3 months ^3^	−12.0	−19.8, −4.2	−4.1	−11.8, 3.6	7.9	−3.4, 19.2	0.17
Difference at 6 months	−2.8	−14.8, 9.1	−14.6	−25.9, −3.4	−11.8	−28.3, 4.8	0.16
Difference at 6 months ^3^	−6.0	−15.9, 3.8	−11.8	−21.0, −2.6	−5.8	−19.5, 7.9	0.40
Protein, g							
Difference at 3 months	−8.4	−44.3, 27.5	−63.1	−98.4, −27.8	−54.7	−106.1, −3.3	0.04
Difference at 3 months ^3^	−20.1	−49.8, 9.7	−51.8	−81.1, −22.5	−31.7	−74.7, 11.3	0.15
Difference at 6 months	14.2	−25.8, 54.3	−94.5	−132.2, −56.9	−108.7	−164.2, −53.3	<0.001
Difference at 6 months ^3^	3.2	−29.2, 35.6	−84.8	−115.3, −54.3	−88.0	−133.2, −42.9	<0.001
Animal origin, g							
Difference at 3 months	1.0	−23.9, 25.8	−39.0	−63.5, −14.5	−40.0	−75.6, −4.3	0.03
Difference at 3 months ^3^	−5.8	−27.9, 16.3	−32.4	−54.2, −10.6	−26.6	−58.5, 5.4	0.10
Difference at 6 months	16.3	−10.6, 43.3	−58.8	−84.2, −33.5	−75.2	−112.5, −37.9	<0.001
Difference at 6 months ^3^	10.3	−13.5, 34.1	−53.5	−75.9, −31.1	−63.8	−97.0, −30.7	<0.001
Vegetable origin, g							
Difference at 3 months	−9.4	−23.9, 5.1	−24.1	−38.4, −9.8	−14.7	−35.5, 6.1	0.16
Difference at 3 months ^3^	−14.2	−26.1, −2.4	−19.4	−31.0, −7.7	−5.1	−22.3, 12.0	0.55
Difference at 6 months	−2.1	−19.3, 15.1	−35.7	−51.8, −19.5	−33.6	−57.3, −9.8	0.01
Difference at 6 months ^3^	−7.1	−20.6, 6.4	−31.3	−43.9, −18.6	−24.2	−42.9, 5.4	0.01
Fat, g							
Difference at 3 months	−4.1	−37.6, 29.5	−50.3	−83.3, −17.2	−46.2	−94.3, 1.9	0.06
Difference at 3 months ^3^	−15.9	−42.4, 10.6	−38.8	−64.8, −12.7	−22.8	−61.1, 15.5	0.24
Difference at 6 months	3.8	−37.7, 45.3	−68.1	−107.2, −29.1	−72.0	−129.4, −14.5	0.02
Difference at 6 months ^3^	−8.8	−40.1, 22.6	−57.0	−86.4, −27.5	−48.2	−91.8, −4.5	0.03
MUFA, g							
Difference at 3 months	−1.7	−13.8, 10.4	−12.0	−24.0, −0.1	−10.3	−27.7, 7.0	0.24
Difference at 3 months ^3^	−5.9	−15.6, 3.9	−8.0	−17.6, 1.6	−2.1	−16.2, 12.0	0.76
Difference at 6 months	−0.1	−16.9, 16.8	−18.3	−34.1, −2.5	−18.3	−41.6, 5.0	0.12
Difference at 6 months ^3^	−5.0	−18.1, 8.1	−14.0	−26.2, −1.7	−9.0	−27.2, 9.2	0.33
PUFA, g							
Difference at 3 months	0.2	−7.5, 7.9	−14.0	−21.6, −6.5	−14.2	−25.3, −3.2	0.01
Difference at 3 months ^3^	−2.2	−8.7, 4.4	−11.8	−18.2, −5.3	−9.6	−19.1, −0.1	0.05
Difference at 6 months	0.2	−8.8, 9.2	−17.5	−26.0, −9.1	−17.7	−30.1, −5.2	0.01
Difference at 6 months ^3^	−2.4	−9.5, 4.7	−15.2	−21.9, −8.6	−12.8	−22.7, −3.0	0.01
Saturated fat, g							
Difference at 3 months	−2.2	−14.6, 10.2	−21.1	−33.3, −8.9	−18.9	−36.7, −1.2	0.04
Difference at 3 months ^3^	−6.6	−16.3, 3.2	−16.8	−26.4, −7.2	−10.3	−24.4, 3.9	0.15
Difference at 6 months	2.7	−11.0, 16.4	−27.7	−40.5, −14.7	−30.3	−49.2, −11.3	0.002
Difference at 6 months ^3^	−1.5	−11.8, 8.9	−23.9	−33.6, −14.2	−22.4	−36.8, −8.0	0.003
Calcium, mg							
Difference at 3 months	−218.9	−735.5, 297.6	−933.3	−1442.1, −424.5	−714.3	−1455.2, 26.5	0.06
Difference at 3 months ^3^	−329.9	−815.5, 155.7	−825.5	−1303.7, 347.3	−495.6	−1197.7, 206.5	0.16
Difference at 6 months	−39.8	−577.5, 498.0	−1379.8	−1885.4, −874.3	−1340.0	−2084.5, −595.6	<0.001
Difference at 6 months ^3^	−178.7	−627.9, 270.4	−1256.8	−1678.7, −834.8	−1078.0	−1703.3, −452.8	0.001
Magnesium, mg							
Difference at 3 months	−89.0	−212.9, 35.0	−210.8	−332.9, −88.7	−121.8	−299.6, 56.0	0.18
Difference at 3 months ^3^	−130.2	−231.8, −28.7	−170.7	−270.7, −70.7	−40.5	−187.3, 106.3	0.58
Difference at 6 months	−20.3	−168.3, 127.6	−348.7	−487.8, −209.6	−328.4	−533.2, −123.6	0.002
Difference at 6 months ^3^	−65.0	−177.3, 47.3	−309.2	−414.7, −203.7	−244.1	−400.5, −87.8	0.003
Sodium, mg							
Difference at 3 months	−1692.8	−3626.8, 241.3	−2465.6	−4370.6, −560.6	−772.9	−3546.5, 2000.8	0.58
Difference at 3 months ^3^	−2373.2	−3906.0, −840.4	−1804.6	−3314.0, −295.2	568.6	−1647.7, 2784.9	0.61
Difference at 6 months	−364.5	−2454.3, 1725.4	−3414.9	−5379.6, −1450.2	−3050.5	−5943.8, −157.2	0.04
Difference at 6 months ^3^	−978.9	−2597.2, 639.4	−2870.7	−4391.0, −1350.4	−1891.7	−4144.6, 361.1	0.10
Potassium, mg							
Difference at 3 months	−625.7	−1751.1, 499.8	−1110.4	−2219.0, −1.9	−484.8	−2098.7, 1129.2	0.55
Difference at 3 months ^3^	−957.1	−1931.7, 17.6	−788.5	−1748.2, 171.3	168.6	−1240.7, 1577.9	0.81
Difference at 6 months	59.1	−1201.0, 1319.2	−2259.0	−3443.6, −1074.4	−2318.1	−4062.6, −573.6	0.01
Difference at 6 months^c^	−284.1	−1308.4, 740.2	−1955.0	−2917.3, −992.7	−1670.9	−3096.8, −245.0	0.02
Iron, mg							
Difference at 3 months	−5.4	−13.3, 2.5	−17.5	−25.2, −9.7	−12.0	−23.3, −0.8	0.04
Difference at 3 months ^3^	−8.2	−14.4, −2.1	−14.7	−20.8, −8.7	−6.5	−15.4, 2.4	0.15
Difference at 6 months	−2.3	−11.4, 6.7	−22.6	−31.1, −14.1	−20.2	−32.7, −7.7	0.002
Difference at 6 months ^3^	−4.9	−12.1, 2.4	−20.3	−27.1, −13.5	−15.4	−25.5, −5.4	0.003
Vitamin C, mg							
Difference at 3 months	−41.8	−118.1, 34.5	−4.9	−80.0, 70.2	36.9	−72.5, 146.3	0.50
Difference at 3 months ^3^	−54.0	−128.4, 20.4	7.0	−66.3, 80.2	61.0	−46.6, 168.5	0.26
Difference at 6 months	11.7	−85.9, 109.3	−43.7	−135.5, 48.0	−55.4	−190.5, 79.7	0.42
Difference at 6 months ^3^	−1.0	−95.9, 93.9	−32.5	−121.6, 56.7	−31.4	−163.6, 100.7	0.64
Vitamin D, mcg							
Difference at 3 months	−0.2	−4.8, 4.4	−4.6	−9.1, −0.1	−4.4	−11.0, 2.2	0.18
Difference at 3 months ^3^	−1.0	−5.4, 3.4	−3.9	−8.2, 0.5	−2.9	−9.3, 3.5	0.37
Difference at 6 months	−0.1	−3.8, 3.5	−9.0	−12.4, −5.6	−8.9	−13.9, −3.9	0.001
Difference at 6 months ^3^	−0.8	−4.2, 2.6	−8.4	−11.6, −5.3	−7.6	−12.2, −2.9	0.002
Vitamin E, IU							
Difference at 3 months	−7.5	−16.4, 1.4	−5.4	−14.2, 3.4	2.1	−10.7, 14.8	0.75
Difference at 3 months ^3^	−9.0	−17.6, −0.4	−3.9	−12.4, 4.6	5.1	−7.3, 17.5	0.42
Difference at 6 months	−5.0	−16.4, 6.3	−13.9	−24.6, 3.2	−8.9	−24.6, 6.9	0.27
Difference at 6 months ^3^	−7.1	−17.7, 3.5	−12.1	−22.1, −2.1	−5.0	−19.8, 9.8	0.50
Folate, mcg							
Difference at 3 months	−53.8	−158.8, 51.2	−31.7	−135.1, 71.7	22.1	−128.4, 172.7	0.77
Difference at 3 months ^3^	−83.3	−175.8, 9.1	−3.0	−94.0, 88.0	80.4	−53.3, 214.0	0.23
Difference at 6 months	−9.6	−138.5, 119.4	−107.8	−229.0, 13.4	−98.2	−276.7, 80.3	0.28
Difference at 6 months ^3^	−44.1	−149.9, 61.7	−77.3	−176.7, 22.2	−33.2	−180.5, 114.1	0.65
HEI−2015 family score							
Difference at 3 months	5.4	1.1, 11.8	7.4	1.0, 13.8	−2.0	−11.9, 7.2	0.66
Difference at 3 months ^3^	6.8	0.8, 12.8	6.0	6.0, 11.9	0.8	−7.8, 9.4	0.85
Difference at 6 months	−0.6	−7.8, 6.7	7.6	0.8, 14.4	−8.2	−18.1, 1.8	0.11
Difference at 6 months ^3^	0.02	−7.2, 7.0	7.1	0.3, 13.9	−7.1	−17.0, 2.9	0.16

Mean difference variable is low minus (−) high avocado allotment group. HEI-2015, healthy eating index 2015; MUFA, monounsaturated fatty acids; PUFA, polyunsaturated fatty acids. ^1^ Sample size by intervention group allocation (low allotment/high allotment): 34/35 at month 3; 31/35 at month 6. ^2^ From ANCOVA model, adjustment for intervention adherence. ^3^ Adjusted for baseline total energy intake.

**Table 8:** Changes in family food group composition, per protocol adherence analysis, in the Effects of Avocado Intake on the Nutritional Status of Families Trial.

	Within-Group Difference				Between-Group Difference		*p*-Value ^2^
	Low Avocado Allotment ^1^		High Avocado Allotment ^1^				
	Mean	95% CI	Mean	95% CI	Mean	95% CI	
Fruit, cup equivalents							
Difference at 3 months	−0.3	−1.0, 0.5	0.7	−0.1, 1.5	−1.0	−2.1, 0.2	0.09
Difference at 3 months ^3^	−0.3	−1.1, 0.5	0.7	−0.1, 1.5	−1.0	−2.2, 0.1	0.08
Difference at 6 months	−0.2	−1.1, 0.7	0.3	−0.6, 1.1	−0.5	−1.7, 0.7	0.42
Difference at 6 months ^3^	−0.3	−1.2, 0.5	0.4	−0.4, 1.2	−0.7	−1.9, 0.5	0.23
Vegetables, cup equivalents							
Difference at 3 months	−0.2	−0.9, 0.6	0.2	−0.6, 0.9	−0.3	−1.4, 0.8	0.55
Difference at 3 months ^3^	−0.2	−1.0, 0.5	0.3	−0.5, 1.0	−0.5	−1.6, 0.6	0.36
Difference at 6 months	0.0	−1.0, 1.0	−0.4	−1.4, 0.5	0.4	−1.0, 1.8	0.57
Difference at 6 months ^3^	−0.1	−1.1, 0.9	−0.3	−1.3, 0.6	0.2	−1.2, 1.6	0.79
Greens, cup equivalents							
Difference at 3 months	−0.1	−0.7, 0.6	0.2	−0.5, 0.8	−0.2	−1.1, 0.7	0.63
Difference at 3 months ^3^	−0.1	−0.7, 0.5	0.2	−0.4, 0.8	−0.3	−1.2, 0.6	0.48
Difference at 6 months	0.1	−0.7, 0.8	−0.3	−1.0, 0.5	0.4	−0.7, 1.4	0.50
Difference at 6 months ^3^	0.0	−0.7, 0.8	−0.2	−0.9, 0.5	0.2	−0.8, 1.2	0.67
Legumes, cup equivalents							
Difference at 3 months	−0.2	−0.4, 0.0	−0.2	−0.4, 0.0	−0.1	−0.3, 0.2	0.66
Difference at 3 months ^3^	−0.3	−0.4, −0.1	−0.2	−0.3, 0.0	−0.1	−0.4, 0.2	0.42
Difference at 6 months	0.0	−0.2, 0.2	−0.3	−0.5, −0.1	0.3	0.0, 0.6	0.05
Difference at 6 months ^3^	0.0	−0.2, 0.2	−0.3	−0.5, −0.1	0.2	0.0, 0.5	0.08
Dairy, cup equivalents							
Difference at 3 months	−0.2	−1.4, 0.9	−1.5	−2.6, −0.4	1.3	−0.4, 2.9	0.12
Difference at 3 months ^3^	−0.4	−1.5, 0.8	−1.4	−2.5, −0.2	1.0	−0.6, 2.7	0.22
Difference at 6 months	0.1	−1.0, 1.3	−2.4	−3.4, −1.3	2.5	1.0, 4.1	0.002
Difference at 6 months ^3^	0.0	−1.1, 1.0	−2.2	−3.2, −1.2	2.2	0.7, 3.7	0.004
Nuts, ounce equivalents							
Difference at 3 months	−0.2	−0.9, 0.5	−0.6	−1.3, 0.1	0.3	−0.6, 1.3	0.47
Difference at 3 months ^3^	−0.3	−1.0, 0.4	−0.5	−1.2, 0.2	0.2	−0.7, 1.2	0.64
Difference at 6 months	−0.3	−1.2, 0.7	−1.0	−1.9, 2.1	0.8	−0.6, 2.1	0.25
Difference at 6 months ^3^	−0.4	−1.3, 0.5	−0.9	−1.8, −0.1	0.5	−0.8, 1.8	0.42
Whole grains, ounce equivalents							
Difference at 3 months	−0.1	−1.0, 0.8	−1.6	−2.4, −0.7	1.4	0.1, 2.7	0.03
Difference at 3 months ^3^	−0.3	−1.2, 0.5	−1.4	−2.2, −0.5	1.0	−0.2, 2.2	0.09
Difference at 6 months	−0.6	−1.8, 0.7	−1.9	−3.1, −0.8	1.4	−0.3, 3.0	0.11
Difference at 6 months ^3^	−0.8	−1.9, 0.4	−1.7	−2.8, −0.7	1.0	−0.6, 2.5	0.23
Refined grains, ounce equivalents							
Difference at 3 months	−2.4	−6.4, 1.6	−8.7	−12.6, −4.7	6.3	0.5, 12.0	0.03
Difference at 3 months ^3^	−3.8	−6.9, −0.6	−7.4	−10.5, −4.3	3.6	−0.9, 8.1	0.12
Difference at 6 months	0.4	−3.8, 4.6	−9.2	−13.2, −5.3	9.6	3.8, 15.4	0.002
Difference at 6 months ^3^	−0.8	−4.0, 2.5	−8.2	−11.3, −5.2	7.4	2.9, 11.9	0.002
Processed meats, ounce equivalents							
Difference at 3 months	0.0	−0.6, 0.6	−0.7	−1.3, −0.1	0.7	−0.2, 1.6	0.11
Difference at 3 months ^3^	−0.2	−0.7, 0.4	−0.6	−1.1, 0.0	0.4	−0.4, 1.2	0.27
Difference at 6 months	0.2	−0.5, 0.9	−0.8	−1.5, −0.1	1.0	0.0, 2.0	0.06
Difference at 6 months ^3^	0.1	−0.6, 0.8	−0.7	−1.4, 0.0	0.8	−0.2, 1.8	0.11
Chicken and eggs, ounce equivalents							
Difference at 3 months	−0.2	−1.1, 0.8	−1.5	−2.4, −0.5	1.3	−0.1, 2.6	0.06
Difference at 3 months ^3^	−0.3	−1.2, 0.6	−1.3	−2.2, −0.4	1.0	−0.3, 2.3	0.14
Difference at 6 months	1.1	−0.3, 2.5	−1.6	−2.9, −0.2	2.7	0.7, 4.7	0.01
Difference at 6 months ^3^	1.0	−0.5, 2.4	−1.5	−2.8, −0.1	2.5	0.5, 4.4	0.02
Fish, ounce equivalents							
Difference at 3 months	0.5	−0.5, 1.5	−0.2	−1.2, 0.8	0.7	−0.7, 2.1	0.32
Difference at 3 months ^3^	−0.3	−0.6, 1.3	−0.1	−1.0, 0.9	0.4	−1.0, 1.7	0.58
Difference at 6 months	0.3	−0.4, 1.1	−0.5	−1.3, 0.2	0.9	−0.2, 1.9	0.11
Difference at 6 months ^3^	0.2	−0.5, 1.0	−0.5	−1.2, 0.3	0.7	−0.4, 1.6	0.19
Red meat, ounce equivalents							
Difference at 3 months	0.1	−1.0 1.0	−0.7	−1.7, 0.3	0.7	−0.7, 2.2	0.32
Difference at 3 months ^3^	−0.2	−1.1, 0.8	−0.5	−1.4, 0.5	0.3	−1.1, 1.7	0.67
Difference at 6 months	0.2	−0.7, 1.0	−1.6	−2.4, −0.7	1.7	−0.5, 2.9	0.01
Difference at 6 months ^3^	0.0	−0.8, 0.8	−1.4	−2.1, −0.7	1.4	−0.3, 2.5	0.01
Sugar, teaspoon equivalents							
Difference at 3 months	−4.2	−12.3, 3.9	−15.4	−23.4, −7.5	11.2	−0.3, 22.8	0.06
Difference at 3 months ^3^	−6.6	−13.4, 0.3	−13.1	−19.9, −6.4	6.6	−3.3, 16.4)	0.19
Difference at 6 months	−4.9	−13.1, 3.2	−12.2	−19.8, −4.5	7.2	−4.0, 18.5	0.20
Difference at 6 months ^3^	−7.1	−13.6, −0.5	−10.3	−16.4, −4.1	3.2	−5.8, 12.2	0.48
Oils, g							
Difference at 3 months	1.6	−9.9. 13.1	−18.7	−30.1, 7.4	20.3	3.9, 36.7	0.02
Difference at 3 months ^3^	−0.3	−11.4, 10.9	−16.9	−27.9, −6.0	16.7	−0.6, 32.7	0.04
Difference at 6 months	−8.4	−22.7, 5.9	−23.1	−36.5, −9.7	14.7	−5.0, 34.4	0.14
Difference at 6 months ^3^	−11.8	−23.8, 0.2	−20.1	−31.3, −8.8	8.3	−8.3, 24.8	0.32
Soymilk, cup equivalents							
Difference at 3 months	0.1	−0.1 to −0.3	0.2	0.0, 0.4	−0.1	−0.4, 0.2	0.59
Difference at 3 months ^3^	0.1	−0.1 to 0.3	0.3	0.0, 0.4	−0.1	−0.4, 0.3	0.73
Difference at 6 months	−0.1	−0.4 to 0.2	0.0	−0.3, 0.2	−0.1	−0.4, 0.3	0.80
Difference at 6 months ^3^	−0.1	−0.4 to 0.2	0.0	−0.3, 0.3	−0.1	−0.5, 0.4	0.78
Soy, ounce equivalents							
Difference at 3 months	0.3	−0.1 to 0.8	0.3	−0.2, 0.8	0.0	−0.7 to 0.6	0.98
Difference at 3 months ^3^	0.3	−0.1 to 0.8	0.3	−0.1, 0.8	0.0	−0.7 to 0.7	0.98
Difference at 6 months	−0.1	−0.8 to 0.6	−0.3	−0.9, 0.4	0.2	−0.8 to 1.1	0.71
Difference at 6 months ^3^	−0.1	−0.8 to 0.6	−0.3	−0.9, 0.4	0.2	−0.8 to 1.1	0.74

Mean difference variable is low minus (−) high avocado allotment group. ^1^ Sample size by intervention group allocation (low allotment/high allotment): 34/35 at month 3; 31/35 at month 6. ^2^ From ANCOVA model, adjustment for intervention adherence. ^3^ Adjusted for baseline total energy intake.

## Data Availability

Data described in the manuscript, code book, and analytic code will be made available upon request pending application and approval. Proposals should be directed to mallison@health.ucsd.edu.

## Supplementary Materials

The following are available online at https://www.mdpi.com/article/10.3390/nu13114021/s1, Supplemental Table S1. Avocado Daily Diary used as an intervention adherence tool in the Effects of Avocado Intake on the Nutritional Status of Families Trial by subgroup; Supplemental Table S2. Socio-demographic characteristics in families that completed and dropped out from the Effects of Avocado Intake on the Nutritional Status of Families Trial study; Supplemental Table S3. Baseline dietary intake of randomized families in the Effects of Avocado Intake on the Nutritional Status of Families Trial by subgroup; Supplemental Table S4. Changes in family energy-adjusted nutrients and food groups intake per intention-to-treat analysis in the Effects of Avocado Intake on the Nutritional Status of Families Trial; Supplemental Table S5. Changes in the nutritional status of heads of households, per intention-to-treat analysis, in the Effects of Avocado Intake on the Nutritional Status of Families Trial; Supplemental Table S6. Changes in the nutritional status of non-head of household adults, per intention-to-treat analysis, in the Effects of Avocado Intake on the Nutritional Status of Families Trial; Supplemental Table S7. Changes in the nutritional status of adolescents, per intention-to-treat analysis, in the Effects of Avocado Intake on the Nutritional Status of Families Trial; Supplemental Table S8. Changes in the nutritional status of children, per intention-to-treat analysis, in the Effects of Avocado Intake on the Nutritional Status of Families Trial; Supplemental Table S9. Changes in the food group composition of heads of households, per intention-to-treat analysis in the Effects of Avocado Intake on the Nutritional Status of Families Trial; Supplemental Table S10. Changes in food group composition of other adults, per intention-to-treat analysis in the Effects of Avocado Intake on the Nutritional Status of Families Trial; Supplemental Table S11. Changes in food group composition of adolescents, per intention-to-treat analysis in the Effects of Avocado Intake on the Nutritional Status of Families Trial; Supplemental Table S12. Changes in food group composition of children, per intention-to-treat analysis in the Effects of Avocado Intake on the Nutritional Status of Families Trial; Supplemental Table S13. Changes in family energy-adjusted food group composition per intention-to-treat analysis in the Effects of Avocado Intake on the Nutritional Status of Families Trial.
