# Supplementary material for: Effects of Different Allotments of Avocados on the Nutritional Status of Families: A Cluster Randomized Controlled Trial
Source: Nutrients. 2021 Nov 11;13(11):4021. doi: 10.3390/nu13114021 (PMC8623192; doi:10.3390/nu13114021)
Supplement: Supplementary file 1 [file nutrients-13-04021-s001.zip › nutrients-1399834-supplementary.pdf]

Supplemental Table S1. Avocado Daily Diary used as an intervention adherence tool in the Effects of Avocado Intake on the Nutritional Status of Families Trial by subgroup.

Week Beginning Date: ____

Please provide us with some information about your family's avocado intake this week, by completing the table below. We are primarily interested in whether or not the foods you and your family prepared today contained avocado, how much avocado was consumed, and how each food was prepared. Additionally, please tell us if you used any of the recipes on the Avocado Recipe Booklet, by writing the recipe and/or page number in area marked "Recipes". Also, please share any other notes and/or comments you would like to share.

<u>Instructions:</u> To be completed by the member of the family most responsible for preparing and/or serving food for the family.
Avocado Intake: Specify if you had avocado that day as a family by circling "Yes" or "No".
Amount of *<u>avocado</u>* that you ate or drank *<u>as a family</u>*: Specify whether it was <¼ (less than ¼), ¼, ½, ¾, 1, 2, or 3 whole avocados, or don't know. For 2 ½ avocado, circle "2" and "½".
Avocado preparation: Circle if it was "Cooked", "Raw", or "Both".

Time/Meal	Did your family consume avocado today?	How much avocado did your family consume today?	How was it prepared?
**Monday**<br>Date:	Yes No	< ¼ ¼ ½ ¾ 1 2 3 don't know<br>Notes:________________________________	Cooked Raw Both<br>Recipes: ______________
**Tuesday**<br>Date:	Yes No	< ¼ ¼ ½ ¾ 1 2 3 don't know<br>Notes: ________________________________	Cooked Raw Both<br>Recipes: ______________
**Wednesday**<br>Date:	Yes No	< ¼ ¼ ½ ¾ 1 2 3 don't know<br>Notes: ________________________________	Cooked Raw Both<br>Recipes: ______________
**Thursday**<br>Date:	Yes No	< ¼ ¼ ½ ¾ 1 2 3 don't know<br>Notes: ________________________________	Cooked Raw Both<br>Recipes: ______________
**Friday**<br>Date:	Yes No	< ¼ ¼ ½ ¾ 1 2 3 don't know<br>Notes: ________________________________	Cooked Raw Both<br>Recipes: ______________
**Saturday**<br>Date:	Yes No	< ¼ ¼ ½ ¾ 1 2 3 don't know<br>Notes: ________________________________	Cooked Raw Both<br>Recipes: ______________
**Sunday**<br>Date:	Yes No	< ¼ ¼ ½ ¾ 1 2 3 don't know<br>Notes: ________________________________	Cooked Raw Both<br>Recipes: ______________
**Weekly Summary**	How many days this week did you eat avocado?<br>____________________	How much avocados does your family have left this week?<br>________________________	
**Comments:** ____			

Supplemental Table S2. Socio-demographic characteristics in families that completed and dropped out from the Effects of Avocado Intake on the Nutritional Status of Families Trial study (n=72).

Characteristic	Families that completed the study		Families that dropped out		*p* value[1]
**Family**					
	**N**	**%**	**N**	**%**	
**Family income in US less than $30,000 dollars/year**	32	44.4	2	2.8	0.48
	**Mean**	**SD**	**Mean**	**SD**	
**Average family size**	3	0.5	3	0.5	0.58
**Head of household**					
	**Mean**	**SD**	**Mean**	**SD**	
**Years lived in the United States**	17.4	12.6	14.2	14.4	0.56
	**N**	**%**	**N**	**%**	
**Female**	65	90.3	6	8.3	0.76
**Country of birth, Mexico**	56	77.8	4	5.6	0.25
**Heritage, Mexican**	64	88.9	6	8.3	0.67
**Marital status**					0.76
Married or cohabitation	44	61.1	4	5.6	
Separated, divorced, or widowed	8	11.1	1	1.4	
Single	14	19.4	1	1.4	
**Highest level of education achieved**					0.85
High school	17	23.6	2	2.8	
Trade school or Associate's degree	19	26.4	1	1.4	
Bachelor's degree or above	15	20.8	1	1.4	
No diploma	7	9.7	0	0	
Other	8	11.1	2	2.8	
**Country where highest level of education was completed**					0.51
United States or other	24	33.3	3	4.2	
Mexico	42	58.3	3	4.2	
**Employment status**					0.93
Employed for wages	16	22.5	3	4.2	
Self-employed	6	8.5	1	1.4	
Homemaker	30	42.3	2	2.8	
Other	13	18.2	0	0	

[1] From unpaired t-test or chi-square where appropriate.

Supplemental Table S3. Baseline dietary intake of randomized families in the Effects of Avocado Intake on the Nutritional Status of Families Trial by subgroup.

Nutrient	Low Avocado Allotment (n=37 families)		High Avocado Allotment (n=35 families)	
**Head of household**	**N**		**N**	
	37		35	
	**Mean**	**SD**	**Mean**	**SD**
**Nutrients**				
Total energy intake, kcal	1,786.8	808.3	1,867.4	1174.9
Carbohydrate, % total energy	49.5	6.2	51.2	7.3
Protein, % total energy	17.4	3.3	18.2	4.4
Fat, % total energy	34.7	5.2	33.3	5.8
Carbohydrate, g	222.6	106.4	241.4	160.5
Dietary fiber, g	26.1	12.6	28.1	14.4
Protein, g	75.6	31.2	80.1	42.0
Animal origin, g	43.7	21.0	45.8	27.8
Vegetable origin, g	31.8	16.0	34.2	23.9
Fat, g	69.1	33.6	70.2	50.3
Saturated fat, g	19.8	10.7	20.7	16.1
Monounsaturated fat, g	28.0	14.0	26.9	19.3
Polyunsaturated fat, g	15.8	8.2	16.9	12.8
Calcium, mg	979.7	437.5	1181.0	953.5
Magnesium, mg	352.1	154.6	405.4	240.4
Sodium, mg	4198.2	3271.6	3595.4	1646.5
Potassium, mg	3054.9	1230.6	3515.9	1725.7
Iron, mg	14.8	7.2	16.8	13.6
Vitamin C, mg	146.7	61.3	181.7	114.6
Vitamin D, mcg	5.9	2.9	6.9	5.9
Vitamin E, IU	18.4	10.9	22.6	25.0
Folate, mcg	283.2	117.5	322.8	171.3
HEI 2015 score, range 0-100	70.9	8.7	71.0	6.7
**Food Groups**				
Fruit, cup equivalents	1.5	0.6	1.5	1.1
Vegetables, cup equivalents	2.2	1.4	2.6	1.5
Greens, cup equivalents	1.7	1.2	1.9	1.2
Legumes, cup equivalents	0.2	0.3	0.3	0.2
Dairy, cup equivalents	1.3	1.0	1.7	1.7
Nuts, ounce equivalents	0.7	0.8	1.2	2.7
Whole grains, ounce equivalents	1.5	1.1	1.6	1.8
Refined grains, ounce equivalents	5.3	4.1	4.6	3.3
Processed meat, ounce equivalents	0.5	0.5	0.4	0.5
Chicken and eggs, ounce equivalents	1.5	0.9	1.4	1.0
Fish, ounce equivalents	1.1	1.0	0.9	0.8
Beef, ounce equivalents	1.0	1.1	1.0	1.2
Sugar, teaspoon equivalents	7.0	4.7	9.8	12.0
Oils, g	25.9	17.4	25.3	22.5
Soymilk, cup equivalents	0.1	0.4	0.1	0.3
Soy, ounce equivalents	0.4	1.3	0.5	1.1
**Non-head of household adults**	**N**		**N**	
	37		32	
	**Mean**	**SD**	**Mean**	**SD**
**Nutrients**				

Total energy intake, kcal	2,682.5	2,085.6	1,971.7	972.7
Carbohydrate, % total energy	50.3	7.9	50.9	5.5
Protein, % total energy	15.6	2.9	17.0	2.7
Fat, % total energy	34.6	5.4	33.2	4.8
Carbohydrate, g	341.6	266.8	252.3	134.1
Dietary fiber, g	32.2	19.8	26.1	15.6
Protein, g	106.6	97.7	83.4	42.6
Animal origin, g	65.1	67.0	51.2	30.5
Vegetable origin, g	41.5	32.1	32.2	22.1
Fat, g	100.6	77.8	72.4	36.3
Saturated fat, g	31.5	26.2	23.3	11.7
Monounsaturated fat, g	39.7	33.2	27.6	14.5
Polyunsaturated fat, g	21.4	13.8	15.3	8.6
Calcium, mg	1,299.8	1,173.2	1,056.2	575.5
Magnesium, mg	441.7	292.9	349.9	167.8
Sodium, mg	5,057.4	4,119.1	3,754.0	2,001.7
Potassium, mg	3,962.6	2,719.1	3,184.9	1,690.2
Iron, mg	20.2	18.4	16.5	11.0
Vitamin C, mg	159.6	127.6	156.7	128.7
Vitamin D, mcg	7.9	9.1	6.5	5.1
Vitamin E, IU	18.9	13.6	17.1	12.7
Folate, mcg	327.1	190.6	274.0	154.9
HEI 2015 score, range 0-100	64.1	9.1	64.0	9.5
**Food Groups**				
Fruit, cup equivalents	1.5	1.1	1.5	1.5
Vegetables, cup equivalents	1.9	1.0	2.0	1.4
Greens, cup equivalents	1.3	0.7	1.3	1.0
Legumes, cup equivalents	0.3	0.3	0.4	0.4
Dairy, cup equivalents	2.0	1.4	2.1	2.7
Nuts, ounce equivalents	0.4	0.5	0.5	0.5
Whole grains, ounce equivalents	1.5	1.0	1.6	1.7
Refined grains, ounce equivalents	6.5	3.6	8.7	9.5
Processed meat, ounce equivalents	0.6	0.5	1.0	2.0
Chicken and eggs, ounce equivalents	1.8	1.1	2.4	2.2
Fish, ounce equivalents	0.8	0.9	1.2	1.8
Beef, ounce equivalents	1.1	1.0	1.7	2.7
Sugar, teaspoon equivalents	11.9	7.6	12.8	15.7
Oils, g	22.4	10.6	30.0	26.7
Soymilk, cup equivalents	0.0	0.0	0.0	0.0
Soy, ounce equivalents	0.2	0.5	0.3	1.1
**Adolescents**	**N**		**N**	
	14		18	
	**Mean**	**SD**	**Mean**	**SD**
**Nutrients**				
Total energy intake, kcal	2,402.8	1,317.5	2,097.3	1,440.7
Carbohydrate, % total energy	53.4	6.8	52.8	8.6
Protein, % total energy	14.6	3.1	15.2	3.1
Fat, % total energy	34.1	5.0	32.5	8.1
Carbohydrate, g	330.3	214.9	275.8	189.3
Dietary fiber, g	22.3	9.6	23.6	14.6
Protein, g	84.8	40.4	74.7	40.3
Animal origin, g	51.7	24.9	38.4	19.7

Vegetable origin, g	33.1	19.9	36.3	31.8
Fat, g	88.1	42.4	70.2	38.4
Saturated fat, g	31.0	16.7	22.4	13.3
Monounsaturated fat, g	31.3	15.1	26.8	14.3
Polyunsaturated fat, g	18.9	10.3	15.3	9.5
Calcium, mg	1,409.4	753.2	1,119.1	603.6
Magnesium, mg	367.4	199.2	344.7	207.9
Sodium, mg	3,772.0	2,004.8	3,728.8	2,700.3
Potassium, mg	3,226.5	1,712.5	2,811.4	1,459.8
Iron, mg	17.4	9.1	18.8	13.7
Vitamin C, mg	115.9	96.0	102.8	58.9
Vitamin D, mcg	8.6	6.0	4.7	3.1
Vitamin E, IU	18.0	18.3	14.4	17.0
Folate, mcg	239.4	128.2	240.9	136.7
HEI 2015 score, range 0-100	59.1	9.8	66.6	12.1
**Food Groups**				
Fruit, cup equivalents	1.4	0.8	1.5	1.2
Vegetables, cup equivalents	1.1	0.7	1.2	0.8
Greens, cup equivalents	0.7	0.5	0.8	0.5
Legumes, cup equivalents	0.2	0.3	0.2	.2
Dairy, cup equivalents	3.0	2.0	1.6	1.0 1
Nuts, ounce equivalents	0.4	0.6	0.2	0.2
Whole grains, ounce equivalents	1.2	0.8	1.9	1.3
Refined grains, ounce equivalents	7.7	5.5	7.0	7.8
Processed meat, ounce equivalents	0.6	0.5	0.7	0.7
Chicken and eggs, ounce equivalents	1.5	1.4	1.4	0.9
Fish, ounce equivalents	0.5	0.9	0.5	0.8
Beef, ounce equivalents	0.7	0.6	0.7	0.7
Sugar, teaspoon equivalents	18.1	22.7	10.5	8.0
Oils, g	27.7	17.2	19.6	11.4
Soymilk, cup equivalents	0.0	0.0	0.1	0.4
Soy, ounce equivalents	0.1	0.2	0.6	1.9

**Children**	**N**		**N**	
	30		28	
	**Mean**	**SD**	**Mean**	**SD**
**Nutrients**				
Total energy intake, kcal	2,136.5	1,209.9	2,507.1	2,164.4
Carbohydrate, % total energy	53.3	8.6	52.8	6.5
Protein, % total energy	15.5	2.4	16.0	2.3
Fat, % total energy	33.4	6.3	33.4	5.1
Carbohydrate, g	279.7	154.1	331.0	278.8
Dietary fiber, g	23.6	14.1	27.0	18.1
Protein, g	83.3	50.2	98.2	89.8
Animal origin, g	54.0	37.3	62.3	62.7
Vegetable origin, g	29.2	15.5	35.9	30.6
Fat, g	80.8	49.1	93.0	61.3
Saturated fat, g	28.1	18.7	31.1	28.3
Monounsaturated fat, g	30.4	18.8	34.4	31.0
Polyunsaturated fat, g	15.7	8.5	19.8	17.6
Calcium, mg	1,389.5	866.3	1,534.4	1,221.1
Magnesium, mg	334.1	196.7	386.6	275.5
Sodium, mg	4,216.4	2,759.1	4,627.9	4,304.3

Potassium, mg	3.070.6	1,793.9	3,624.2	2,370.7
Iron, mg	16.6	8.8	19.9	19.8
Vitamin C, mg	121.7	67.2	147.4	92.5
Vitamin D, mcg	8.7	6.1	10.0	9.5
Vitamin E, IU	14.4	7.2	15.7	11.3
Folate, mcg	232.8	129.4	296.3	229.1
HEI 2015 score, range 0-100	63.1	9.4	65.5	11.5
**Food Groups**				
Fruit, cup equivalents	2.0	1.4	1.9	1.2
Vegetables, cup equivalents	1.1	0.7	1.7	1.5
Greens, cup equivalents	0.7	0.5	1.2	1.1
Legumes, cup equivalents	0.2	0.2	0.2	0.2
Dairy, cup equivalents	2.6	1.9	2.9	2.5
Nuts, ounce equivalents	0.4	0.5	0.4	0.6
Whole grains, ounce equivalents	1.9	2.3	1.6	1.3
Refined grains, ounce equivalents	6.0	3.5	7.8	9.1
Processed meat, ounce equivalents	0.6	0.6	0.8	1.3
Chicken and eggs, ounce equivalents	2.0	1.9	2.2	2.8
Fish, ounce equivalents	0.6	0.8	0.7	0.9
Beef, ounce equivalents	0.9	0.7	0.9	1.2
Sugar, teaspoon equivalents	11.8	8.1	13.8	13.7
Oils, g	21.7	12.7	24.1	16.3
Soymilk, cup equivalents	0.0	0.1	0.1	0.7
Soy, ounce equivalents	0.2	0.5	0.5	1.6

HEI-2015, healthy eating index score 2015.

Supplemental Table S4. Changes in family energy-adjusted[1] nutrients and food groups intake per intention-to-treat analysis in the Effects of Avocado Intake on the Nutritional Status of Families Trial (n=72).

	Within-group differences				Between-group difference		*p*-value[2]
	Low Avocado Allotment (n=37)		High Avocado Allotment (n=35)				
	Mean	(95% CI)	Mean	(95% CI)	Mean	(95% CI)	
**Carbohydrate, % kcal**							
Difference at 3 months	-3.1	-5.3, -0.9	-2.2	-4.2, -0.2	-0.9	-3.8, 2.0	0.56
Difference at 3 months[3]	-3.1	-5.1, -1.0	-2.2	-4.3, -0.1	-0.9	-3.8, 2.1	0.56
Difference at 6 months	-2.2	-4.0, -0.4	-1.1	-3.0, 0.8	-1.1	-3.7, 1.4	0.39
Difference at 6 months[3]	-2.1	-3.9, -0.4	-1.2	-3.0, 0.7	-0.9	-3.6, 1.6	0.44
**Protein, % kcal**							
Difference at 3 months	0.2	-0.7, 1.0	0.2	-0.8, 1.1	0.0	-1.2, 1.2	0.99
Difference at 3 months[3]	0.2	-0.6, 1.1	0.1	-0.7, 1.0	0.1	-1.2, 1.3	0.90
Difference at 6 months	0.3	-0.5, 1.0	-0.6	-1.7, 0.6	0.9	-0.5, 2.3	0.19
Difference at 6 months[3]	0.4	-0.6, 1.3	-0.7	-1.6, 0.2	1.1	-0.3, 2.4	0.11
**Fat, % kcal**							
Difference at 3 months	0.9	-0.9, 2.6	2.9	1.1, 4.7	-2.0	-4.5, 0.4	0.11
Difference at 3 months[3]	0.9	-0.8, 2.6	2.9	1.3, 4.7	-2.0	-4.5, 0.5	0.11
Difference at 6 months	0.4	-1.2, 2.0	2.8	0.7, 4.7	-2.4	-4.9, 0.2	0.07
Difference at 6 months[3]	0.3	-1.4, 2.0	2.8	1.0, 4.6	-2.5	-5.0, 0.001	0.05
**Carbohydrate, g**							
Difference at 3 months	-5.7	-10.3, -1.1	-5.5	-10.3, -0.8	-0.1	-6.7, 6.4	0.97
Difference at 6 months	-5.5	-10.0, -1.1	-2.7	-7.3, 1.9	-2.8	-9.2, 3.6	0.39
**Dietary fiber, g**							
Difference at 3 months	-0.2	-0.9, 0.5	2.2	1.5, 2.9	-2.5	-3.5, -1.5	<0.0001
Difference at 6 months	-0.3	-1.0, 0.5	2.2	1.4, 3.0	-2.5	-3.6, -1.4	<0.0001
**Protein, g**							
Difference at 3 months	0.9	-1.0, 2.9	0.4	-1.5, 2.4	0.5	-2.3, 3.3	0.72
Difference at 6 months	0.7	-1.7, 3.0	-1.6	-4.0, 0.9	2.2	-1.3, 5.6	0.19
**Animal origin, g**							
Difference at 3 months	1.3	-0.8, 3.4	0.1	-2.0, 2.2	1.2	-1.8, 4.2	0.42
Difference at 6 months	1.5	-0.9, 3.9	-1.4	-3.8, 1.1	2.9	-0.5, 6.3	0.10
**Vegetable origin, g**							
Difference at 3 months	-0.4	-1.2, 0.5	0.4	-0.5, -1.2	-0.7	-1.9, 0.5	0.25
Difference at 6 months	-0.8	-1.8, 0.1	-0.2	-1.1, 0.8	-0.6	-2.0, 0.7	0.34
**Fat, g**							
Difference at 3 months	1.5	-0.2, 3.2	3.2	1.5, 4.9	-1.7	-4.1, 0.8	0.16
Difference at 6 months	0.4	-1.5, 2.4	3.0	1.0, 5.0	-2.6	-5.4, 0.2	0.07
**MUFA, g**							

Difference at 3 months	0.6	-0.3, 1.6	2.7	1.7, 3.6	-2.0	-3.4, 0.7	0.003
Difference at 6 months	0.1	-1.0, 1.2	2.9	1.7, 4.0	-2.8	-4.4, -1.2	0.0009
**PUFA, g**							
Difference at 3 months	0.4	-0.1, 0.9	-0.02	-0.5, 0.5	0.5	-0.3, 1.2	0.20
Difference at 6 months	0	-0.5, 0.5	-0.1	-0.6, 0.4	0.1	-0.6, 0.8	0.79
**Saturated fat, g**							
Difference at 3 months	0.3	-0.4, 1.0	0.1	-0.5, 0.8	0.2	-0.8, 1.1	0.72
Difference at 6 months	0.2	-0.5, 0.9	-0.2	-0.9, 0.6	0.4	-0.7, 1.4	0.49
**Calcium, mg**							
Difference at 3 months	-23.5	-65.0, 17.9	-14.8	-57.4, 27.8	-8.8	-68.2, 50.7	0.77
Difference at 6 months	-34.3	-85.6, 17.0	-22.4	-75.1, 30.3	-11.9	-85.4, 61.6	0.75
**Magnesium, mg**							
Difference at 3 months	-1.2	-7.6, 5.3	8.5	1.9, 15.2	-9.7	-19.0, -0.4	0.04
Difference at 6 months	-3.1	-10.0, 3.8	2.6	-4.5, 9.7	-5.7	-15.6, 4.2	0.26
**Sodium, mg**							
Difference at 3 months	-77.2	-220.4, 66.1	128.5	-18.8, 275.7	-205.6	-411.1, -0.2	0.05
Difference at 6 months	-42.7	-162.5, 77.0	119.5	-3.7, 242.6	-162.2	-334.0, 9.6	0.06
**Potassium, mg**							
Difference at 3 months	2.2	-75.0, 79.3	234.0	154.7, 313.3	-231.8	-342.5, -121.2	<0.0001
Difference at 6 months	-1.9	-79.9, 76.0	196.1	116.0, 276.2	-198.0	-309.8, -86.3	0.0007
**Iron, mg**							
Difference at 3 months	-0.5	-1.0, 0.1	-0.7	-1.3, -0.2	0.3	-0.5, 1.1	0.51
Difference at 6 months	-0.7	-1.3, -0.2	-0.9	-1.4, -0.3	0.1	-0.6, 0.9	0.71
**Vitamin C, mg**							
Difference at 3 months	-3.8	-13.0, 5.4	13.2	3.7, 22.6	-17.0	-30.2, -3.8	0.01
Difference at 6 months	1.3	-11.6, 14.1	16.6	3.3, 29.8	-15.3	-33.7, 3.2	0.10
**Vitamin D, mcg**							
Difference at 3 months	0.1	-0.4, 0.5	0.2	-0.2, 0.7	-0.2	-0.8, 0.5	0.63
Difference at 6 months	-0.1	-0.5, 0.4	-0.2	-0.7, 0.3	0.1	-0.5, 0.8	0.67
**Vitamin E, IU**							
Difference at 3 months	-0.8	-1.7, 0.2	1.0	0.01, 2.0	-1.8	-3.1, -0.4	0.01
Difference at 6 months	-1.0	-2.1, 0.02	0.8	-0.3, 1.9	-1.8	-3.3, -0.3	0.02
**Folate, mcg**							
Difference at 3 months	0.4	-8.4, 9.1	33.4	24.4, 42.4	-33.0	-45.5, -20.5	<0.0001
Difference at 6 months	-2.1	-11.9, 7.7	35.8	25.7, 45.9	-37.9	-51.9, -23.8	<0.0001

Mean difference variable is low minus (-) high avocado allotment group. HEI-2015, healthy eating index 2015; MUFA, monounsaturated fatty acids; PUFA, polyunsaturated fatty acids.

[1] Energy adjusted method: nutrient density as proportion (%) of macronutrient per total energy intake and/or nutrient density of macro- and micronutrient as intake of nutrient (in appropriate units)/1000 kcals.

[2] From ANOVA model.

Supplemental Table S5. Changes in the nutritional status of heads of households, per intention-to-treat analysis, in the Effects of Avocado Intake on the Nutritional Status of Families Trial (n=72).

	Within-group differences		Mean between-group difference (95% CI)	p-value[1]
	Low Avocado Allotment Mean (95% CI) (n=37)	High Avocado Allotment Mean (95% CI) (n=35)		
**Total energy intake, kcal**				
Difference at 3 months	-221.6 (-539.7 to 96.6)	-117.5 (-444.6 to 209.6)	-104.0 (-560.4 to 352.2)	0.65
Difference at 6 months	-1262.7 (-640.3 to 36.7)	-391.8 (-739.8 to -43.7)	89.9 (-395.6 to 575.4)	0.71
**Carbohydrate, % kcal**				
Difference at 3 months	-0.7 (-2.6 to 1.3)	-2.1 (-5.4 to 1.2)	1.5 (-2.2 to 5.2)	0.44
Difference at 3 months[2]	-0.6 (-3.2 to -2.0)	-2.1 (-4.8 to 0.5)	1.5 (-2.2 to 5.2)	0.42
Difference at 6 months	0.2 (-2.3 to 2.7)	-2.5 (-4.7 to -0.3)	2.7 (-0.6 to 6.0)	0.11
Difference at 6 months[2]	0.2 (-2.1 to 2.5)	-2.5 (-4.9 to -0.1)	2.7 (-0.6 to 6.0)	0.11
**Protein, % kcal**				
Difference at 3 months	0.8 (-0.2 to 1.9)	-1.2 (-2.7 to 0.3)	2.0 (0.3 to 3.8)	0.03
Difference at 3 months[2]	0.8 (-0.4 to 2.1)	-1.2 (-2.5 to 0.01)	2.1 (0.3 to 3.9)	0.02
Difference at 6 months	0.5 (-0.8 to 1.8)	-0.6 (-2.3 to 1.2)	1.1 (-1.1 to 3.2)	0.32
Difference at 6 months[2]	0.5 (-0.9 to 2.0)	-0.6 (-2.1 to 0.9)	1.1 (-0.9 to 3.2)	0.28
**Fat, % kcal**				
Difference at 3 months	-0.5 (-2.2 to 1.2)	3.6 (1.0 to 6.2)	-4.1 (-7.2 to -1.1)	0.01
Difference at 3 months[2]	-0.6 (-2.7 to 1.5)	3.6 (1.5 to 5.8)	-4.2 (-7.2 to -1.3)	0.01
Difference at 6 months	-0.7 (-2.7 to 1.3)	3.5 (1.7 to 5.3)	-4.2 (-6.9 to -1.6)	0.002
Difference at 6 months[2]	-0.8 (-2.6 to 1.1)	3.6 (1.7 to 5.4)	-4.3 (-7.0 to -1.7)	0.002
**Carbohydrate, g**				
Difference at 3 months	-32.7 (-73.3to 8.0)	-21.4 (-63.3 to 20.4)	-11.2 (-69.6 to 47.1)	0.70
Difference at 3 months[2]	-35.7 (-67.7 to -3.6)	-18.2 (-51.2to 14.7)	-17.4 (-63.4 to 28.6)	0.54
Difference at 6 months	-37.2 (-81.6 to 7.2)	-56.8 (-102.4 to -11.2	19.6 (-44.0 to 83.3)	0.54
Difference at 6 months[2]	-41.0 (-71.8 to -10.2)	-52.8 (-84.4 to -21.1)	11.8 (-32.5 to 55.9)	0.60
**Dietary fiber, g**				
Difference at 3 months	-3.3 (-7.3 to 0.7)	3.7 (-0.4 to 7.9)	-7.0 (-12.8 to 1.3)	0.02
Difference at 3 months[2]	-3.5 (-7.0 to 0.1)	4.0 (0.5 to 7.5)	-7.5 (-12.5 to -2.6)	0.003
Difference at 6 months	-3.5 (-7.9 to 0.9)	-0.8 (-5.3 to 3.7)	-2.7 (-9.0 to 3.6)	0.40
Difference at 6 months[2]	-3.8 (-7.3 to -0.3)	-0.4 (-4.1 to 3.2)	-3.3 (-8.4 to 1.7)	0.19
**Protein, g**				
Difference at 3 months	-5.7 (-17.6 to 6.2)	-8.8 (-21.1 to 3.4)	3.1 (-14.0 to 20.3)	0.72
Difference at 3 months[2]	-6.5 (-16.5 to 3.5)	-8.0 (-18.2 to 2.3)	1.5 (-12.8 to 15.8)	0.84
Difference at 6 months	-9.3 (-22.5 to 4.0)	-17.7 (-31.3 to -4.0)	8.4 (-10.6 to 27.5)	0.38
Difference at 6 months[2]	-10.2 (-20.9 to 0.4)	-16.7 (-27.6 to -5.7)	6.4 (-8.8 to 21.7)	0.40
**Animal origin, g**				
Difference at 3 months	-1.4 (-9.7 to 6.9)	-6.5 (-15.1 to 2.0)	5.1 (-6.8 to 17.0)	0.39
Difference at 3 months[2]	-1.8 (-9.4 to 5.7)	-6.1 (-13.9 to -1.7)	4.3 (-6.6 to 15.1)	0.44
Difference at 6 months	-3.4 (-12.1 to 5.3)	-11.1 (-20.0 to -2.2)	7.7 (-4.8 to 20.2)	0.22
Difference at 6 months[2]	-3.8 (-11.8 to 4.1)	-10.6 (-18.8 to -2.4)	6.8 (-4.6 to 18.2)	0.24
**Vegetable origin, g**				
Difference at 3 months	-4.3 (-9.7 to 1.2)	-2.3 (-7.9 to 3.3)	-2.0 (-9.8 to 5.9)	0.62
Difference at 3 months[2]	-4.7 (-9.1 to -0.2)	-1.9 (-6.5 to 2.7)	-2.8 (-9.2 to 3.6)	0.39
Difference at 6 months	-5.9 (-12.3 to 0.6)	-6.6 (-13.2 to 0.1)	0.7 (-8.5 to 10.0)	0.88
Difference at 6 months[2]	-6.4 (-11.1 to -1.7)	-6.0 (-10.9 to -1.2)	-0.4 (-7.2 to 6.4)	0.91
**Fat, g**				
Difference at 3 months	-8.8 (-23.6 to 5.9)	0.4 (-14.7 to 15.6)	-9.3 (-30.4 to 11.9)	0.39

Difference at 3 months[2]	-9.9 (-21.6 to 1.8)	1.6 (-10.5 to 13.6)	-11.5 (-28.3 to 5.3)	0.18
Difference at 6 months	-13.7 (-28.1 to 0.6)	-11.0 (-25.7 to 3.8)	-2.8 (-23.3 to 17.8)	0.79
Difference at 6 months[2]	-15.0 (-24.9 to -5.0)	-9.6 (-19.9 to 0.6)	-5.3 (-19.6 to 8.9)	0.46
**MUFA, g**				
Difference at 3 months	-4.4 (-9.9 to 1.2)	3.5 (-2.2 to 9.3)	-7.9 (-15.9 to 0.04)	0.05
Difference at 3 months[2]	-4.8 (-9.3 to -0.3)	4.0 (-0.7 to 8.6)	-8.7 (-15.2 to -2.3)	0.01
Difference at 6 months	-6.0 (-11.8 to 0.2)	-1.2 (-7.1 to 4.8)	4.8 (-13.1 to 3.4)	0.25
Difference at 6 months[2]	-6.5 (-10.6 to -2.3)	-0.7 (-4.9 to 3.6)	-5.8 (-11.8 to 0.1)	0.05
**PUFA, g**				
Difference at 3 months	-2.2 (-5.7 to 1.4)	-1.3 (-4.9 to 2.4)	-0.9 (-6.0 to 4.2)	0.73
Difference at 3 months[2]	-2.4 (-5.2 to 0.4)	-1.0 (-3.9 to 1.9)	-1.4 (-5.4 to 2.6)	0.48
Difference at 6 months	-4.0 (-7.5 to 0.5)	-3.9 (-7.5 to 0.4)	-0.1 (-5.0 to 4.9)	0.98
Difference at 6 months[2]	-4.3 (-6.7 to -1.8)	-3.6 (-6.1 to -1.1)	-0.7 (-4.2 to 2.9)	0.71
**Saturated fat, g**				
Difference at 3 months	-1.8 (-7.0 to 3.4)	-2.3 (-7.6 to 3.1)	0.5 (-7.0 to 7.9)	0.90
Difference at 3 months[2]	-2.2 (-6.4 to 2.1)	-1.9 (-6.3 to 2.5)	-0.3 (-6.4 to 5.9)	0.94
Difference at 6 months	-3.0 (-7.6 to 1.6)	-5.5 (-10.3 to 0.8)	2.5 (-4.1 to 9.1)	0.45
Difference at 6 months[2]	-3.4 (-6.7 to -0.1)	-5.1 (-8.5 to -1.7)	1.7 (-3.0 to 6.5)	0.48
**Calcium, mg**				
Difference at 3 months	-132.8 (-370.0 to 104.4)	-270.3 (-514.2 to 26.4)	137.5 (-202.7 to 477.7)	0.42
Difference at 3 months[2]	-147.2 (-352.7 to 58.2)	-255.0 (-466.3 to -43.8)	107.8 (-187.0 to 402.6)	0.47
Difference at 6 months	-132.1 (-382.5. to 118.2)	-407.6 (-665.0 to -150.2)	275.5 (-83.6 to 634.6)	0.13
Difference at 6 months[2]	-149.4 (-355.3 to 56.6)	-389.4 (-601.1 to -177.6)	240.0 (-55.5 to 535.5)	0.11
**Magnesium, mg**				
Difference at 3 months	-39.6 (-95.8 to 16.6)	-24.9 (-82.7 to 32.9)	-14.6 (-95.3 to 66.0)	0.72
Difference at 3 months[2]	-44.0 (-86.3 to -1.8)	-20.2 (-63.6 to 23.2)	-23.8 (84.4 to 36.7)	0.44
Difference at 6 months	-42.6 (-107.2 to 22.0)	-86.6 (-153.0 to 20.3)	44.0 (-48.6 to 136.6)	0.35
Difference at 6 months[2]	-48.2 (-93.2 to -3.2)	-80.8 (-127.0 to -34.5)	32.6 (-32.0 to 97.2)	0.32
**Sodium, mg**				
Difference at 3 months	-870.2 (-1744.2 to 3.7)	109.6 (-789.0 to 1088.1)	-979.8 (-2233.3 to 273.7)	0.12
Difference at 3 months[2]	-908.9 (-1726.8 to -91.0)	150.4 (-690.5 to 991.4)	-1059.3 (-2232.9 to 114.3)	0.08
Difference at 6 months	-840.3 (-1675.0 to -5.6)	-260.5 (-1118.7 to 597.7)	-579.8 (-1777.0 to 617.4)	0.34
Difference at 6 months[2]	-886.7 (-1630.9 to -142.4)	-211.5 (-976.7 to 553.8)	-675.2 (-1743.1 to 392.7)	0.21
**Potassium, mg**				
Difference at 3 months	-257.1 (-725.4 to 211.1)	239.9 (-241.6 to 721.4)	-497.0 (-1168.7 to 174.6)	0.14
Difference at 3 months[2]	--283.7 (-698.8 to 131.4)	268.0 (-158.9 to 694.8)	-551.7 (-1147.3 to 44.0)	0.07
Difference at 6 months	-217.5 (-736.9 to 302.0)	-291.7 (-825.8 to 242.4)	74.2 (-670.8 to -819.3)	0.84
Difference at 6 months[2]	-254.8 (-684.7 to 179.9)	-254.8 (-699.2 to 189.7)	2.4 (-617.9 to 622.7)	0.99
**Iron, mg**				
Difference at 3 months	-3.1 (-6.1 to 0.003)	-3.3 (-6.4 to -0.1)	0.2 (-4.2 to 4.6)	0.93
Difference at 3 months[2]	-3.3 (-5.8 to -0.7)	-3.0 (-5.7 to -0.4)	-0.2 (-3.9 to 3.5)	0.90
Difference at 6 months	-3.4 (-6.9 to 0.2)	5.4 (-9.1 to -1.8)	2.1 (-3.0 to 7.2)	0.42
Difference at 6 months[2]	-3.6 (-6.4 to -0.9)	-5.2 (-8.0 to -2.3)	1.5 (-2.4 to 5.5)	0.45
**Vitamin C, mg**				
Difference at 3 months	-6.3 (-39.7 to 27.1)	26.5 (-7.8 to 60.8)	-32.8 (-80.7 to 15.1)	0.18
Difference at 3 months[2]	-7.1 (-40.1 to 26.0)	27.2 (-6.8 to 61.3)	-34.3 (-81.8 to 13.2)	0.15
Difference at 6 months	2.9 (-43.3 to 49.1)	0.3 (-47.3 to 47.7)	2.7 (-63.5 to 68.9)	0.94
Difference at 6 months[2]	2.1 (-43.9 to 48.1)	1.0 (-46.3 to 48.4)	1.1 (-65.0 to 67.1)	0.98
**Vitamin D, mcg**				
Difference at 3 months	-0.5 (-2.2 to 1.1)	-0.9 (-2.6 to 0.8)	0.4 (-2.0 to 2.7)	0.77
Difference at 3 months[2]	-0.6 (-2.1 to 0.9)	-30.8(-2.3 to 0.8)	0.2 (-2.0 to 2.3)	0.88
Difference at 6 months	-0.6 (-2.4 to 1.2)	-2.6 (-4.4 to -0.7)	2.0 (-0.6 to 4.6)	0.12

Difference at 6 months[2]	-0.7 (-2.3 to 1.0)	-2.5 (-4.2 to 0.8)	1.8 (-0.5 to 4.2)	0.13
**Vitamin E, IU**				
Difference at 3 months	-3.7 (-8.9 to 1.4)	-1.7 (-7.0 to 3.5)	-2.0 (-9.4 to 5.4)	0.59
Difference at 3 months[2]	-4.1 (-8.4 to 0.3)	-1.4 (-5.8 to 3.1)	-2.7 (-8.9 to 3.5)	0.39
Difference at 6 months	-3.4 (-9.5 to 2.8)	-6.2 (-12.6 to 0.1)	2.9 (-6.0 to 11.7)	0.52
Difference at 6 months[2]	-3.8 (-8.8 to 1.3)	-5.8 (-10.9 to -0.6)	2.0 (-5.3 to 9.2)	0.59
**Folate, mcg**				
Difference at 3 months	-28.1 (-68.8 to 12.6)	45.0 (3.2 to 86.9)	-73.1 (-131.5 to -14.7)	0.02
Difference at 3 months[2]	-30.3 (-66.7 to 6.0)	47.4 (10.0 to 84.8)	-77.7 (-129.9 to -25.5)	0.004
Difference at 6 months	-29.5 (-77.6 to 18.6)	8.0 (-41.5 to 57.5)	-37.5 (-106.5 to 31.5)	0.28
Difference at 6 months[2]	-32.8 (-72.8 to 7.3)	11.4 (-29.8 to 52.6)	-44.2 (-101.7 to 13.3)	0.13
**HEI-2015 family score**				
Difference at 3 months	0.6 (-2.2 to 3.5)	2.9 (0.1 to 5.6)	-2.2 (-6.3 to 1.8)	0.27
Difference at 3 months[2]	0.8 (-2.0 to 3.5)	2.7 (0.02 to 5.4)	-2.0 (-5.9 to 2.0)	0.32
Difference at 6 months	-1.6 (-4.6 to 1.5)	2.6 (-0.3 to 5.4)	-4.1 (-8.3 to 0.1)	0.05
Difference at 6 months[2]	-1.5 (-4.6 to 1.6)	2.5 (-0.4 to 5.4)	-4.0 (-8.3 to 0.2)	0.06

Mean difference variable is low minus (-) high avocado allotment group. HEI-2015, healthy eating index 2015; MUFA, monounsaturated fatty acids; PUFA, polyunsaturated fatty acids.

[1] From unpaired t-test or ANCOVA model (adjusted for baseline total energy intake), where appropriate.

[2] Adjusted for baseline total energy intake.

Supplemental Table S6. Changes in the nutritional status of non-head of household adults, per intention-to-treat analysis, in the Effects of Avocado Intake on the Nutritional Status of Families Trial (n=69).

	Within-group differences		Mean between-group difference (95% CI)	p-value[1]
	Low Avocado Allotment Mean (95% CI) (n=37)	High Avocado Allotment Mean (95% CI) (n=32)		
**Total energy intake, kcal**				
Difference at 3 months	-696.9 (-1,165.4 to -228.4)	-95.3 (-599.1 to -408.5)	-601.7 (-1,289.6 to 86.3)	0.09
Difference at 6 months	-680.6 (-1,148.8 to -212.5)	-83.5 (-586.8 to -419.9)	-597.2 (-1,284.6 to 90.3)	0.09
**Carbohydrate, % kcal**				
Difference at 3 months	-1.6 (-3.9 to 0.7)	-2.8 (-5.4 to -0.2)	1.2 (-2.2 to 4.6)	0.49
Difference at 3 months[2]	-1.3 (-3.6 to 1.0)	-3.1 (-5.6 to -0.6)	1.8 (-1.6 to 5.3)	0.29
Difference at 6 months	-0.5 (-2.5 to -1.6)	-2.8 (-5.7 to 0.01)	2.4 (-1.0 to 5.8)	0.17
Difference at 6 months[2]	-0.3 (-2.6 to 2.1)	-3.1 (-5.6 to -0.6)	2.8 (-0.7 to 6.3)	0.11
**Protein, % kcal**				
Difference at 3 months	0.3 (-0.5 to 1.1)	-0.1 (-1.2 to 0.9)	0.5 (-0.8 to 1.7)	0.48
Difference at 3 months[2]	0.3 (-0.6 to 1.2)	-0.1 (-1.1 to 0.8)	0.4 (-0.9 to 1.7)	0.54
Difference at 6 months	0.4 (-0.4 to 1.3)	-0.3 (-1.7 to 1.2)	0.7 (-0.9 to 2.4)	0.39
Difference at 6 months[2]	0.4 (-0.7 to 1.5)	-0.3 (-1.4 to 0.9)	0.7 (-0.9 to 2.3)	0.40
**Fat, % kcal**				
Difference at 3 months	0.9 (-1.1 to 2.9)	2.9 (0.9 to 5.0)	-2.0 (-4.8 to 0.8)	0.17
Difference at 3 months[2]	0.7 (-1.2 to 2.6)	3.2 (1.2 to 5.3)	-2.6 (-5.4 to 0.3)	0.08
Difference at 6 months	0.1 (-1.9 to 2.2)	2.6 (0.5 to 4.8)	2.5 (-5.4 to 0.4)	0.09
Difference at 6 months[2]	-0.1 (-2.1 to 1.9)	2.9 (0.8 to 5.0)	-3.0 (-5.9 to -0.02)	0.05
**Carbohydrate, g**				
Difference at 3 months	-101.8 (-164.9 to -38.6)	-23.4 (-91.3 to 44.5)	-78.3 (-171.1 to 14.4)	0.10
Difference at 3 months[2]	-70.1 (-105.7 to -34.4)	-60.1 (-98.4 to -21.8)	-10.0 (-63.0 to 43.0)	0.71
Difference at 6 months	-92.3 (-150.9 to 33.7)	-23.2 (-86.2 to 39.8)	-69.0 (-155.1 to 17.0)	0.11
Difference at 6 months[2]	-64.3 (-100.9 to -27.8)	-55.5 (-94.9 to -16.1)	-8.8 (-63.2 to -45.5)	0.75
**Dietary fiber, g**				
Difference at 3 months	-5.8 (-11.0 to -0.7)	-1.4 (-6.9 to 4.2)	-4.5 (-12.1 to 3.1)	0.24
Difference at 3 months[2]	-3.5 (-6.9 to -0.8)	-4.1 (-7.8 to -0.4)	0.6 (-4.5 to 5.7)	0.82
Difference at 6 months	-7.8 (-12.9 to -2.7)	-0.9 (-6.4 to 4.6)	-6.9 (-14.4 to 0.6)	0.07
Difference at 6 months[2]	-5.6 (-9.2 to -2.0)	-3.5 (-7.4 to 0.3)	-2.1 (-7.4 to 3.2)	0.44
**Protein, g**				
Difference at 3 months	-25.9 (-46.7 to -5.1)	-6.1 (-28.4. to -16.3)	-19.9 (-50.4 to 10.7)	0.20
Difference at 3 months[2]	-15.6 (-27.5 to -3.6)	-18.0 (-30.9 to -5.2)	2.5 (-15.3 to 20.2)	0.78
Difference at 6 months	-25.7 (-47.9 to -3.5)	-4.4 (-28.2 to 19.5)	-21.3 (-53.9 to 11.2)	0.20
Difference at 6 months[2]	-16.2 (-32.2 to -0.3)	-15.3 (-32.5 to 1.9)	-0.9 (-24.6 to 22.8)	0.94
**Animal origin, g**				
Difference at 3 months	-16.8 (-31.0 to -2.7)	-3.2 (-18.4 to 12.1)	-13.7 (-34.5 to 7.2)	0.20
Difference at 3 months[2]	-10.3 (-19.7 to -1.0)	-10.7 (-20.8 to -0.6)	0.4 (-13.5 to 14.3)	0.07
Difference at 6 months	-14.3 (-29.8 to 1.2)	-2.7 (-19.3 to 13.9)	-11.6 (-34.3 to 11.1)	0.31
Difference at 6 months[2]	-8.2 (-20.2 to 3.7)	-9.7 (-22.6 to 3.2)	1.5 (-16.3 to -19.2)	0.87
**Vegetable origin, g**				
Difference at 3 months	-9.1 (-16.8 to -1.4)	-2.9 (-11.1 to 5.4)	-6.2 (-17.5 to 5.1)	0.28
Difference at 3 months[2]	-5.3 (-9.6 to -0.9)	-7.3 (-12.0 to -2.6)	-2.1 (-4.4 to 8.6)	0.53
Difference at 6 months	-11.4 (-19.1 to -3.6)	-1.7 (-10.0 to 6.6)	-9.7 (-21.1 to 1.7)	0.09
Difference at 6 months[2]	-8.0 (-13.5 to 2.6)	-5.6 (-11.5 to 0.2)	-2.4 (-10.5 to 5.7)	0.56
**Fat, g**				

Difference at 3 months	-22.3 (-39.3 to -5.4)	1.4 (-16.9 to 19.6)	-23.7 (-48.6 to 1.2)	0.06
Difference at 3 months[2]	-14.3 (-25.1 to -3.6)	-7.9 (-19.5 to 3.6)	-6.4 (-22.4 to 9.5)	0.43
Difference at 6 months	-23.2 (-41.3 to -5.1)	1.9 (-17.5 to 21.4)	-25.1 (-51.7 to 1.4)	0.06
Difference at 6 months[2]	-15.7 (-29.2 to -2.3)	-6.7 (-21.2 to 7.8)	-9.1 (-29.1 to 11.0)	0.37
**MUFA, g**				
Difference at 3 months	-8.1 (-15.4 to -0.8)	2.3 (-5.5 to 10.2)	-10.4 (-21.1 to 0.3)	0.06
Difference at 3 months[2]	-4.7 (-9.4 to 0.01)	-1.6 (-6.7 to 3.4)	-3.1 (-10.0 to 3.9)	0.38
Difference at 6 months	-9.4 (-17.3 to -1.6)	2.4 (-6.1 to 10.8)	-11.8 (-23.3 to -0.3)	0.05
Difference at 6 months[2]	-6.1 (-11.9 to -0.4)	-1.4 (-7.6 to 4.8)	-4.7 (-13.3 to 3.8)	0.27
**PUFA, g**				
Difference at 3 months	-4.6 (-8.0 to -1.2)	-0.1 (-3.8 to 3.5)	-4.4 (-9.4 to 0.6)	0.08
Difference at 3 months[2]	-3.1 (-5.5 to -0.7)	-1.9 (-4.4 to 0.7)	-1.2 (-4.8 to 2.3)	0.49
Difference at 6 months	-4.9 (-8.6 to -1.3)	0.5 (-3.5 to -4.4)	-5.4 (-10.7 to -0.02)	0.05
Difference at 6 months[2]	-3.5 (-6.3 to -0.7)	-1.2 (-4.2 to 1.8)	-2.2 (-6.4 to 1.9)	0.28
**Saturated fat, g**				
Difference at 3 months	-8.1 (-13.7 to -2.6)	-0.8 (-6.7 to 5.2)	-7.3 (-15.5 to 0.8)	0.08
Difference at 3 months[2]	-5.6 (-9.3 to -1.9)	-3.7 (-7.7 to 0.3)	-1.9 (-7.4 to 3.6)	0.49
Difference at 6 months	-7.3 (-13.1 to -1.6)	-1.1 (-7.3 to 5.1)	-6.2 (-14.7 to 2.2)	0.15
Difference at 6 months[2]	-5.2 (-9.7 to -0.6)	-3.6 (-8.5 to 1.3)	-1.5 (-8.3 to 5.2)	0.65
**Calcium, mg**				
Difference at 3 months	-382.1 (-666.8 to -97.4)	-55.1 (-361.3 to 251.0)	-327.0 (-745.0 to 91.1)	0.12
Difference at 3 months[2]	-261.0 (-466.6 to -55.4)	-195.2 (-416.6 to 26.3)	-65.8 (-371.4 to 239.7)	0.67
Difference at 6 months	-297.3 (-555.2. to -39.4)	-87.5 (-364.8 to 189.8)	-209.8 (-588.5 to 168.9)	0.27
Difference at 6 months[2]	-193.6 (-389.3 to 2.2)	-207.4 (-418.3 to -3.4)	-13.9 (-277.1 to 304.8)	0.92
**Magnesium, mg**				
Difference at 3 months	-96.9 (-164.5 to -29.3)	-15.0 (-87.7 to 57.7)	-81.9 (-181.1 to 17.3)	0.10
Difference at 3 months[2]	-65.3 (-108.7 to -21.8)	-51.6 (-98.3 to -4.8)	-13.7 (-78.3 to 50.8)	0.67
Difference at 6 months	-108.6 (-178.1 to 39.1)	-9.0 (-83.7 to 65.7)	-99.6 (-201.6 to 2.5)	0.06
Difference at 6 months[2]	-79.6 (-130.8 to -28.5)	-42.5 (-97.6 to 12.6)	-37.1 (-113.2 to 38.9)	0.33
**Sodium, mg**				
Difference at 3 months	-1025.9 (-1957.7 to -94.0)	-414.5 (-1416.5 to 587.5)	-611.4 (-1979.8 to 756.9)	0.38
Difference at 3 months[2]	-608.1 (-1242.9 to 26.8)	-897.5 (-1581.3 to -213.8)	289.5 (-654.1 to 1233.0)	0.54
Difference at 6 months	-1331.3 (-2268.6 to -394.0)	-217.7 (-1225.6 to 790.2)	-1113.6 (-2490.0 to 262.8)	0.11
Difference at 6 months[2]	-933.3 (-1611.4 to -255.3)	-677.9 (-1408.2 to 52.4)	-255.5 (-1263.3 to 752.3)	0.62
**Potassium, mg**				
Difference at 3 months	-856.1 (-1473.2 to -239.0)	-58.3 (-721.9 to 605.2)	-797.8 (-1703.9 to 108.4)	0.08
Difference at 3 months[2]	-574.1 (-984.2 to -164.0)	-384.4 (-826.1 to 57.3)	-189.6 (-799.1 to 419.9)	0.54
Difference at 6 months	-953.8 (-1605.6 to 302.1)	-51.6 (-752.5 to 649.2)	-902.2 (-1859.2 to 54.9)	0.06
Difference at 6 months[2]	-680.9 (-1158.8 to -203.1)	-367.2 (-881.8 to 147.5)	-313.8 (-1024.0 to 396.4)	0.38
**Iron, mg**				
Difference at 3 months	-6.1 (-10.2 to -2.0)	-2.6 (-7.1 to 1.8)	-3.4 (-9.4 to 2.6)	0.26
Difference at 3 months[2]	-4.0 (-6.3 to -1.7)	-5.0 (-7.5 to -2.5)	1.0 (-2.4 to 4.5)	0.55
Difference at 6 months	-5.2 (-9.2 to -1.2)	-2.5 (-6.8 to 1.7)	-2.7 (-8.5 to 3.2)	0.37
Difference at 6 months[2]	-3.6 (-6.5 to -0.6)	-4.4 (-7.6 to -1.3)	0.9 (-3.5 to 5.3)	0.69
**Vitamin C, mg**				
Difference at 3 months	-27.8 (-57.7 to 2.1)	-17.0 (-49.1 to 15.1)	-10.8 (-54.7 to 33.1)	0.63
Difference at 3 months[2]	-17.3 (-42.0 to 7.4)	-29.1 (-55.8 to -2.5)	-11.8 (-24.9 to 48.6)	0.52
Difference at 6 months	-30.2 (-64.2 to 3.8)	-10.4 (-46.9 to 26.2)	-19.9 (-69.8 to 30.1)	0.43
Difference at 6 months[2]	-22.8 (-55.1 to 9.5)	-18.9 (-53.7 to 16.9)	-3.9 (-51.9 to 44.1)	0.87
**Vitamin D, mcg**				
Difference at 3 months	-2.7 (-5.2 to -0.1)	0.7 (-2.0 to 3.5)	-3.4 (-7.2 to 0.4)	0.08
Difference at 3 months[2]	-1.9 (-4.1 to 0.4)	-0.2 (-2.6 to 2.2)	-1.6 (-4.9 to 1.7)	0.33

Difference at 6 months	-2.2 (-4.2 to -0.3)	0.1 (-2.0 to 2.2)	-2.3 (-5.2 to 0.5)	0.11
Difference at 6 months[2]	-1.5 (-3.2 to 0.1)	-0.7 (-2.4 to 1.1)	-0.9 (-3.2 to 1.6)	0.48
**Vitamin E, IU**				
Difference at 3 months	-3.3 (-7.2 to 0.6)	-1.3 (-5.5 to 2.9)	-2.0 (-7.7 to 3.7)	0.48
Difference at 3 months[2]	-2.2 (-5.6 to 1.3)	-2.6 (-6.3 to 1.1)	0.4 (-4.7 to 5.6)	0.86
Difference at 6 months	-3.2 (-7.5 to 1.2)	-0.5 (-5.2 to 4.2)	-2.6 (-9.0 to 3.7)	0.41
Difference at 6 months[2]	-2.5 (-6.8 to 1.8)	-1.3 (-5.9 to 3.3)	-1.2 (-7.6 to 5.1)	0.70
**Folate, mcg**				
Difference at 3 months	-48.4 (-93.4 to -3.4)	-6.7 (-55.1 to 41.7)	-41.7 (-107.8 to 24.4)	0.21
Difference at 3 months[2]	-30.0 (-63.6 to 3.7)	-28.0 (-64.2to 8.3)	-2.0 (-52.1 to 48.0)	0.94
Difference at 6 months	-81.3 (-134.0 to 28.5)	4.5 (-52.1 to 61.2)	-85.8 (-163.2 to 8.4)	0.03
Difference at 6 months[2]	-62.3 (-105.5 to -19.2)	-17.3 (-63.8 to 29.2)	-45.0 (-109.2 to 19.1)	0.17
**HEI-2015 family score**				
Difference at 3 months	1.9 (-0.5 to 4.2)	4.4 (2.0 to 6.8)	-2.6 (-5.9 to 0.8)	0.13
Difference at 3 months[2]	2.0 (-0.3 to 4.4)	4.3 (1.9 to 6.6)	-2.2 (-5.6 to 1.2)	0.19
Difference at 6 months	1.1 (-2.0 to 4.2)	3.3 (0.3 to 6.3)	-2.2 (-6.5 to 2.1)	0.31
Difference at 6 months[2]	0.9 (-2.2 to 3.9)	3.5 (0.5 to 6.5)	-2.7 (-7.0 to 1.7)	0.23

Mean difference variable is low minus (-) high avocado allotment group. HEI-2015, healthy eating index 2015; MUFA, monounsaturated fatty acids; PUFA, polyunsaturated fatty acids.

[1] From unpaired t-test or ANCOVA model (adjusted for baseline total energy intake), where appropriate.

[2] Adjusted for baseline total energy intake.

Supplemental Table S7. Changes in the nutritional status of adolescents, per intention-to-treat analysis, in the Effects of Avocado Intake on the Nutritional Status of Families Trial (n=32).

	Within-group differences		Mean between-group difference (95% CI)	p-value[1]
	Low Avocado Allotment Mean (95% CI) (n=14)	High Avocado Allotment Mean (95% CI) (n=18)		
**Total energy intake, kcal**				
Difference at 3 months	-59.6 (-667.8 to 548.6)	-408.4 (-944.8 to 128.0)	348.8 (-462.2 to 1,159.8)	0.39
Difference at 6 months	-308.4 (-880.4 to 263.7)	-443.5 (-948.0 to 61.0)	135.1 (-627.7 to 879.9)	0.72
**Carbohydrate, % kcal**				
Difference at 3 months	-3.0 (-8.8 to 2.7)	-3.6 (-9.6 to 2.5)	0.5 (-7.6 to 8.7)	0.90
Difference at 3 months[2]	-2.8 (-9.0 to 3.4)	-3.7 (-9.2 to 2.7)	0.9 (-7.3 to 9.2)	0.82
Difference at 6 months	-2.1 (-7.5 to 3.2)	-0.6 (-6.8 to 5.6)	-1.6 (-9.7 to 6.6)	0.70
Difference at 6 months[2]	-1.9 (-8.0 to 4.2)	-0.8 (-6.2 to 4.6)	-1.1 (-9.2 to 7.1)	0.79
**Protein, % kcal**				
Difference at 3 months	-0.2 (-2.9 to 2.6)	1.0 (-1.7 to 3.7)	-1.2 (-5.0 to 2.5)	0.51
Difference at 3 months[2]	-0.4 (-3.1 to 2.4)	1.2 (-1.2 to 3.6)	-1.6 (-5.2 to 2.1)	0.40
Difference at 6 months	-0.2 (-2.4 to 2.1)	-0.3 (-3.2 to 2.5)	0.1 (-3.5 to 3.9)	0.92
Difference at 6 months[2]	-0.4 (-3.0 to 2.2)	-0.2 (-2.5 to 2.1)	-0.2 (-3.7 to 033)	0.89
**Fat, % kcal**				
Difference at 3 months	1.9 (-2.9 to 6.8)	4.9 (0.7 to 9.1)	-3.0 (-9.1 to 3.1)	0.33
Difference at 3 months[2]	1.5 (-2.8 to 5.7)	5.2 (1.5 to 9.0)	-3.8 (-9.4 to 1.9)	0.19
Difference at 6 months	2.3 (-1.3 to 5.8)	2.8 (-2.6 to 8.3)	-0.5 (-6.8 to 5.7)	0.87
Difference at 6 months[2]	1.9 (-3.0 to 6.7)	3.1 (-1.1 to 7.4)	-1.2 (-7.7 to 5.2)	0.70
**Carbohydrate, g**				
Difference at 3 months	-29.3 (-111.3 to 52.7)	-71.6 (-143.9 to 0.8)	42.2 (-67.1 to 151.6)	0.44
Difference at 3 months[2]	-18.3 (-85.7 to -49.2)	-80.1 (-139.6 to 20.7)	61.9 (-28.3 to 152.1)	0.17
Difference at 6 months	-61.5 (-143.2 to 20.1)	-55.4 (-127.4 to -16.6)	6.2 (-115.0 to 102.7)	0.91
Difference at 6 months[2]	-51.4 (-121.3 to -18.4)	-63.3 (-124.8 to -1.7)	11.9 (-81.5 to 105.2)	0.80
**Dietary fiber, g**				
Difference at 3 months	1.2 (-4.7 to 7.0)	-2.0 (-7.2 to 3.1)	3.2 (-4.6 to 11.0)	0.41
Difference at 3 months[2]	1.6 (-4.0 to 7.2)	-2.4 (-7.3 to 2.6)	4.0 (-3.5 to 11.5)	0.29
Difference at 6 months	1.4 (-6.1 to 8.9)	-3.7 (-10.2 to 2.9)	5.1 (-4.9 to 15.0)	0.31
Difference at 6 months[2]	1.6 (-6.0 to 9.2)	-3.8 (-10.5 to 2.9)	5.4 (4.8 to 15.5)	0.29
**Protein, g**				
Difference at 3 months	1.3 (-21.8 to 24.3)	-3.4 (-23.7 to 16.9)	4.7 (-26.1 to 35.4)	0.76
Difference at 3 months[2]	1.9 (-21.4 to 25.3)	-3.9 (-24.5 to 16.7)	5.9 (-25.4 to 37.1)	0.70
Difference at 6 months	-7.0 (-24.9 to 11.0)	-16.3 (-32.1 to -0.4)	9.3 (-14.7 to 33.2)	0.43
Difference at 6 months[2]	-6.3 (-24.4 to 11.8)	-16.8 (-32.7 to -0.8)	10.4 (-13.8 to 34.7)	0.39
**Animal origin, g**				
Difference at 3 months	-0.4 (-16.2 to 15.4)	2.0 (-11.9 to 16.0)	-2.4 (-23.5 to 18.7)	0.82
Difference at 3 months[2]	-0.4 (-16.6 to 15.7)	-2.1 (-12.2 to 16.3)	-2.5 (-24.1 to 19.1)	0.81
Difference at 6 months	-5.7 (-16.7 to 5.3)	-6.5 (-16.2 to 3.3)	0.8 (-14.0 to 15.5)	0.92
Difference at 6 months[2]	-5.6 (-16.9 to 5.7)	-6.5 (-16.5 to 3.4)	1.0 (-14.1 to 16.0)	0.90
**Vegetable origin, g**				
Difference at 3 months	1.7 (-10.3 to 13.6)	-5.4 (-16.0 to 5.1)	7.1 (-8.8 to 23.0)	0.37
Difference at 3 months[2]	2.4 (-9.4 to 14.1)	-6.0 (-16.3 to 4.3)	8.4 (-7.3 to 24.1)	0.28
Difference at 6 months	-1.3 (-10.9 to 8.4)	-9.8 (-18.3 to -1.3)	8.5 (-4.4 to 21.4)	0.19
Difference at 6 months[2]	-0.7 (-10.3 to 8.8)	-10.2 (-18.7 to -1.8)	9.5 (-3.3 to 22.3)	0.14

**Fat, g**				
Difference at 3 months	1.2 (-19.7 to 22.0)	-0.2 (-18.6 to 18.2)	1.4 (-26.4 to 29.1)	0.92
Difference at 3 months[2]	1.4 (-19.8 to 22.7)	-0.4 (-19.1 to 18.3)	1.8 (-26.6 to 30.2)	0.90
Difference at 6 months	-4.8 (-22.8 to 13.2)	-6.3 (-22.2 to 9.6)	1.5 (-22.5 to 25.5)	0.90
Difference at 6 months[2]	-4.3 (-22.5 to 14.0)	-6.7 (-22.8 to 9.3)	2.5 (-21.9 to 26.9)	0.84
**MUFA, g**				
Difference at 3 months	3.6 (-4.9 to 12.0)	1.7 (-5.8 to 9.1)	1.9 (-9.4 to 13.2)	0.73
Difference at 3 months[2]	3.6 (-5.0 to 12.3)	1.6 (-6.0 to 9.2)	2.0 (-9.5 to 13.6)	0.72
Difference at 6 months	0.2 (-8.1 to 8.5)	-0.1 (-7.4 to 7.2)	0.2 (-10.8 to 11.3)	0.96
Difference at 6 months[2]	0.4 (-8.0 to 8.8)	-0.3 (-7.7 to 7.1)	0.7 (-10.7 to 11.9)	0.90
**PUFA, g**				
Difference at 3 months	1.9 (-3.9 to 7.7)	-0.1 (-5.2 to 5.0)	2.0 (-5.7 to 9.8)	0.59
Difference at 3 months[2]	1.8 (-4.1 to 7.7)	-0.03 (-5.2 to 5.2)	1.8 (-6.1 to 9.7)	0.64
Difference at 6 months	-0.3 (-4.7 to 3.7)	-1.1 (-4.7 to 2.4)	0.8 (-4.5 to 6.2)	0.75
Difference at 6 months[2]	-0.3 (-4.4 to 3.8)	-1.1 (-4.7 to 2.5)	0.8 (-4.7 to 6.2)	0.78
**Saturated fat, g**				
Difference at 3 months	-1.2 (-9.6 to 7.2)	-2.0 (-9.4 to 5.4)	0.8 (-10.3 to 12.0)	0.88
Difference at 3 months[2]	-0.7 (-9.0 to 7.6)	-2.4 (-9.7 to 4.9)	1.7 (-9.5 to 12.8)	0.76
Difference at 6 months	-4.5 (-11.1 to 2.1)	-4.6 (-10.5 to 1.2)	0.1 (-8.7 to 8.9)	0.98
Difference at 6 months[2]	-4.1 (-10.6 to 2.4)	-4.9 (-10.7 to 0.8)	0.8 (-7.9 to 9.5)	0.85
**Calcium, mg**				
Difference at 3 months	-88.3 (-520.5 to 354.0)	-235.7 (-625.7 to 154.4)	147.4 (-442.3 to 737.1)	0.61
Difference at 3 months[2]	-50.7 (-469.9 to 368.4)	-264.9 (-634.3 to 104.5)	214.2 (-346.2 to 774.6)	0.44
Difference at 6 months	-212.0 (-509.2. to 85.1)	-304.9 (-567.0 to -42.8)	92.9 (-303.3 to 489.1)	0.64
Difference at 6 months[2]	-180.8 (-450.0 to 88.4)	-329.2 (-566.4 to -92.0)	148.4 (-211.5 to 508.3)	0.41
**Magnesium, mg**				
Difference at 3 months	-32.7 (-125.4 to 60.1)	-68.6 (-150.3 to 13.2)	35.9 (-87.7 to 159.5)	0.56
Difference at 3 months[2]	-23.8 (-109.6 to 62.1)	-75.5 (-151.2 to 0.2)	51.7 (63.0to 166.5)	0.36
Difference at 6 months	-34.2 (-116.8 to 48.4)	-92.6 (-165.5 to -19.8)	58.4 (-51.7 to 168.5)	0.29
Difference at 6 months[2]	-26.8 (-104.2 to 50.7)	-98.4 (-166.7 to -30.2)	71.7 (-31.9 to 175.2)	0.17
**Sodium, mg**				
Difference at 3 months	-137.9 (-1124.9 to 849.2)	-447.4 (-1317.9 to 423.0)	309.6 (-1006.5 to 1625.6)	0.63
Difference at 3 months[2]	-74.8 (-1042.9 to 893.2)	-496.5 (-1349.5 to 356.6)	421.6 (-872.6 to 1715.9)	0.51
Difference at 6 months	-305.2 (-1353.0 to 742.6)	-371.0 (-1295.0 to 553.1)	65.8 (-1331.3 to 1462.8)	0.92
Difference at 6 months[2]	-280.8 (-1346.1 to 784.5)	-389.9 (-1328.8 to 548.9)	109.1 (-1315.2 to 1533.5)	0.88
**Potassium, mg**				
Difference at 3 months	-276.1 (-1005.5 to 453.4)	-166.7 (-810.0 to 476.6)	-109.4 (-1082.0 to 863.3)	0.82
Difference at 3 months[2]	-224.9 (-934.1 to 484.2)	-206.5 (-831.4 to 418.4)	-18.5 (-966.6 to 929.7)	0.97
Difference at 6 months	-214.9 (-993.2 to 563.3)	-475.4 (-1161.7 to 211.0)	260.5 (-777.2 to 1298.1)	0.61
Difference at 6 months[2]	-172.0 (-943.7 to 599.6)	-508.7 (-1188.7 to 171.3)	336.9 (-695.1 to 1368.4)	0.51
**Iron, mg**				
Difference at 3 months	0.2 (-6.3 to 6.7)	-5.2 (-10.9 to 0.6)	5.4 (-3.3 to 14.0)	0.22
Difference at 3 months[2]	0.8 (-5.4 to 6.9)	-5.6 (-11.0 to -0.2)	6.4 (-1.8 to 14.6)	0.12
Difference at 6 months	-2.7 (-8.1 to 2.6)	-6.4 (-11.1 to -1.7)	3.7 (-3.5 to 10.8)	0.31
Difference at 6 months[2]	-2.3 (-7.4 to 2.8)	-6.7 (-11.2 to -2.2)	4.4 (-2.4 to 11.3)	0.20
**Vitamin C, mg**				
Difference at 3 months	-21.6 (-71.6 to 28.4)	-15.6 (-59.7 to 28.5)	-6.0 (-72.7 to 60.6)	0.86
Difference at 3 months[2]	-7.1 (-40.1 to 26.0)	-20.8 (-71.7 to 30.2)	-16.2 (-61.1 to 28.7)	0.89
Difference at 6 months	17.3 (-64.5 to 99.2)	-11.7 (-83.8 to 60.6)	28.9 (-80.3 to 138.0)	0.59
Difference at 6 months[2]	15.4 (-67.8 to 98.7)	-10.1 (-83.4 to 63.3)	25.5 (-85.8 to 136.8)	0.64
**Vitamin D, mcg**				
Difference at 3 months	-1.1 (-3.6 to 1.4)	-1.8 (-4.0 to 0.4)	0.7 (-2.7 to 4.0)	0.69

Difference at 3 months[2]	-0.9 (-3.2 to 1.4)	-1.9 (-4.0 to 0.1)	1.1 (-2.1 to 4.2)	0.50
Difference at 6 months	-2.2 (-4.6 to 0.2)	-2.4 (-4.5 to -0.3)	0.2 (-3.0 to 3.4)	0.91
Difference at 6 months[2]	-1.8 (-3.8 to 0.1)	-2.6 (-4.3 to 0.9)	0.8 (-1.8 to 3.4)	0.54
**Vitamin E, IU**				
Difference at 3 months	-0.3 (-8.8 to 8.2)	-1.2 (-8.6 to 6.3)	0.9 (-10.5 to 12.2)	0.88
Difference at 3 months[2]	-0.8 (-9.2 to 7.6)	-0.8 (-8.1 to 6.6)	-0.04 (-11.2 to 11.2)	0.99
Difference at 6 months	-0.4 (-5.6 to 4.9)	-3.5 (-8.1 to 1.1)	3.1 (-3.9 to 10.1)	0.37
Difference at 6 months[2]	-0.5 (-5.8 to 4.8)	-3.4 (-8.1 to -1.3)	2.9 (-4.2 to 9.9)	0.42
**Folate, mcg**				
Difference at 3 months	15.6 (-54.6 to 85.8)	8.6 (-53.3 to 70.5)	7.0 (-86.6 to 100.6)	0.88
Difference at 3 months[2]	17.8 (-53.3 to 88.9)	6.9 (-55.7 to 69.6)	10.9 (-84.2 to 105.9)	0.82
Difference at 6 months	12.8 (-67.9 to 93.5)	-26.0 (-97.2 to 45.1)	38.9 (-68.8 to 146.5)	0.47
Difference at 6 months[2]	14.9 (-67.0 to 96.9)	-27.7 (-99.9 to 44.5)	42.6 (-66.9 to 152.2)	0.43
**HEI-2015 family score**				
Difference at 3 months	4.4 (-2.0 to 10.8)	-2.3 (-7.2 to 2.7)	6.7 (-1.7 to 15.0)	0.11
Difference at 3 months[2]	4.5 (-1.4 to 10.4)	-2.3 (-6.9 to 2.2)	6.9 (-0.8 to 14.5)	0.08
Difference at 6 months	6.4 (-1.2 to 14.0)	-2.9 (-8.6 to 2.7)	9.3 (-0.1 to 18.8)	0.05
Difference at 6 months[2]	6.2 (-1.4 to 13.9)	-2.8 (-8.5 to 2.9)	9.0 (-0.5 to 18.6)	0.06

Mean difference variable is low minus (-) high avocado allotment group. HEI-2015, healthy eating index 2015; MUFA, monounsaturated fatty acids; PUFA, polyunsaturated fatty acids.

[1] From unpaired t-test or ANCOVA model (adjusted for baseline total energy intake), where appropriate.

[2]Adjusted for baseline total energy intake.

Supplemental Table S8. Changes in the nutritional status of children, per intention-to-treat analysis, in the Effects of Avocado Intake on the Nutritional Status of Families Trial (n=58).

	Within-group differences		Mean between-group difference (95% CI)	p-value[1]
	Low Avocado Allotment Mean (95% CI) (n=30)	High Avocado Allotment Mean (95% CI) (n=28)		
**Total energy intake, kcal**				
Difference at 3 months	-44.7 (-653.7 to 564.4)	-745.7 (-1,376.1 to 115.3)	701.0 (-175.6 to 1,577.5)	0.12
Difference at 6 months	150.4 (-536.3 to 837.0)	-859.6 (-1,570.4 to 148.9)	1,010.0 (21.7 to 1,998.3)	0.05
**Carbohydrate, % kcal**				
Difference at 3 months	-3.3 (-6.8 to 0.2)	-2.9 (-6.4 to 0.6)	-0.4 (-5.2 to 4.5)	0.88
Difference at 3 months[2]	-3.2 (-6.6 to 0.2)	-3.0 (-6.6 to 0.5)	-0.2 (-5.1 to 4.7)	0.94
Difference at 6 months	-1.6 (-4.6 to -1.4)	-2.3 (-6.8 to 2.2)	0.7 (-4.5 to 5.9)	0.79
Difference at 6 months[2]	-1.3 (-4.9 to 2.2)	-2.6 (-6.3 to 1.1)	1.2 (-3.9 to 6.4)	0.63
**Protein, % kcal**				
Difference at 3 months	0.3 (-0.6 to 1.2)	1.5 (-0.2 to 3.2)	-1.2 (-3.1 to 0.7)	0.19
Difference at 3 months[2]	0.3 (-1.0 to 1.6)	1.6 (0.2 to 2.9)	-1.3 (-3.2 to 0.6)	0.17
Difference at 6 months	0.4 (-0.5 to 1.3)	-0.7 (-2.7 to 1.2)	1.1 (-1.0 to 3.3)	0.29
Difference at 6 months[2]	0.4 (-1.0 to 1.9)	-0.8 (-2.3 to 0.8)	1.2 (-0.9 to 3.3)	0.27
**Fat, % kcal**				
Difference at 3 months	2.9 (0.1 to 5.6)	1.5 (-1.4 to 4.4)	1.3 (-2.6 to 5.3)	0.50
Difference at 3 months[2]	2.8 (0.1 to 5.6)	1.6 (-1.3 to 4.4)	1.3 (-2.7 to 5.3)	0.53
Difference at 6 months	0.9 (-1.6 to 3.4)	3.6 (-0.5 to 7.6)	-2.7 (-7.4 to 2.0)	0.26
Difference at 6 months[2]	0.7 (-2.4 to 3.8)	3.9 (0.6 to 7.1)	-3.2 (-7.7 to 1.3)	0.16
**Carbohydrate, g**				
Difference at 3 months	-14.8 (-97.1 to 67.5)	-103.4 (-188.6 to -18.2)	88.6 (-29.8 to 207.1)	0.14
Difference at 3 months[2]	-29.9 (-93.1 to 33.2)	-87.2 (-152.6 to -21.9)	57.3 (-33.9 to 148.4)	0.21
Difference at 6 months	16.1 (-73.5 to 105.7)	-116.4 (-209.2 to -23.7)	132.5 (3.6 to 261.5)	0.04
Difference at 6 months[2]	0.3 (-70.4 to 71.1)	-99.5 (-172.8 to -26.3)	99.9 (-2.3 to 202.0)	0.06
**Dietary fiber, g**				
Difference at 3 months	-1.7 (-7.3 to 3.9)	-6.9 (-12.7 to -1.2)	5.2 (-2.8 to 13.2)	0.19
Difference at 3 months[2]	-2.6 (-7.2 to 2.0)	-6.0 (-10.8 to -1.2)	3.4 (-3.3 to 10.0)	0.31
Difference at 6 months	0.7 (-5.7 to 7.1)	-6.1 (-12.7 to 0.5)	6.8 (-2.4to 16.0)	0.15
Difference at 6 months[2]	-0.3 (-5.8 to 5.3)	-5.1 (-10.9 to 0.6)	4.8 (3.2 to 12.9)	0.23
**Protein, g**				
Difference at 3 months	-0.6 (-25.7 to 24.4)	-24.4 (-50.3 to 1.5)	23.7 (-12.3 to 59.8)	0.19
Difference at 3 months[2]	-5.6 (-23.6 to 12.3)	-19.0 (-37.6 to -0.5)	13.4 (-12.5 to 39.3)	0.30
Difference at 6 months	9.2 (-20.4 to 38.9)	-36.1 (-66.8 to -5.4)	45.3 (2.6 to 88.0)	0.04
Difference at 6 months[2]	4.0 (-19.5 to 27.5)	-30.5 (-54.9 to 6.2)	34.5 (0.6 to 68.5)	0.05
**Animal origin, g**				
Difference at 3 months	0.9 (-17.0 to 18.8)	-12.6 (-31.1 to 6.0)	13.5 (-12.3 to 39.3)	0.30
Difference at 3 months[2]	-2.4 (-16.1 to 11.2)	-9.0 (-23.1 to 5.1)	6.6 (-13.1 to 26.3)	0.51
Difference at 6 months	6.0 (-14.8 to 26.9)	-22.1 (-43.7to -0.6)	28.2 (-1.8 to 58.1)	0.07
Difference at 6 months[2]	2.6 (-14.5 to 19.8)	-18.5 (-36.2 to -0.7)	21.1 (-3.6 to 45.9)	0.09
**Vegetable origin, g**				
Difference at 3 months	-1.6 (-10.4 to 7.3)	-11.8 (-21.0 to -2.6)	10.3 (-2.5 to 23.0)	0.11
Difference at 3 months[2]	3.2 (-9.9 to 3.5)	-10.0 (-17.0 to 3.1)	6.8 (-2.8 to 16.5)	0.16
Difference at 6 months	3.2 (-7.1 to 13.4)	-14.0 (-24.6 to -3.4)	17.1 (-2.4 to 31.9)	0.02
Difference at 6 months[2]	1.4 (-6.8 to 9.5)	-12.1 (-20.5 to -3.6)	13.4 (1.7 to 25.2)	0.03
**Fat, g**				
Difference at 3 months	1.7 (-21.1 to 24.4)	-27.1 (-50.6 to 3.6)	28.8 (-3.9 to 61.5)	0.08

Difference at 3 months^2	-2.9 (-19.1 to 13.4)	-22.3 (-39.1 to 5.4)	19.4 (-4.1 to 42.9)	0.10
Difference at 6 months	4.9 (-21.1 to 30.9)	-28.2 (-55.1 to -1.3)	33.1 (-4.4 to 70.5)	0.08
Difference at 6 months^2	-0.02 (-19.6 to 19.6)	-23.0 (-43.3 to 2.7)	23.0 (-5.4 to 51.3)	0.11
**MUFA, g**				
Difference at 3 months	-0.2 (-8.9 to 8.5)	-8.1 (-17.1 to 0.9)	7.9 (-4.6 to 20.5)	0.21
Difference at 3 months^2	-1.9 (-8.1 to 4.2)	-6.2 (-12.6 to 0.2)	4.3 (-4.6 to 13.2)	0.34
Difference at 6 months	1.1 (-8.9 to 11.2)	-7.4 (-17.8 to 3.0)	8.5 (-5.9 to 23.0)	0.24
Difference at 6 months^2	-0.7 (-8.4 to 6.9)	-5.4 (-13.3 to 2.5)	4.7 (-6.4 to 15.7)	0.40
**PUFA, g**				
Difference at 3 months	1.5 (-3.6 to 6.5)	-7.4 (-12.6 to 2.2)	8.9 (1.7 to 16.1)	0.02
Difference at 3 months^2	0.5 (-3.3 to 4.4)	-6.5 (-10.5 to 2.4)	7.0 (1.4 to 12.6)	0.02
Difference at 6 months	1.4 (-4.4 to 7.2)	-7.2 (-13.2 to 1.2)	8.6 (0.2 to 16.9)	0.05
Difference at 6 months^2	0.4 (-4.2 to 5.0)	-6.1 (-10.8 to 1.3)	6.5 (-0.2 to 13.1)	0.06
**Saturated fat, g**				
Difference at 3 months	0.3 (-7.7 to 8.3)	-9.3 (-17.6 to -1.0)	9.6 (-1.9 to 21.1)	0.10
Difference at 3 months^2	-1.2 (-7.3 to 4.9)	-7.7 (-14.0 to -1.4)	6.5 (-2.3 to 15.2)	0.14
Difference at 6 months	2.0 (-6.9 to 10.8)	-11.3 (-20.4 to 2.1)	13.2 (0.5 to 25.9)	0.04
Difference at 6 months^2	-4.1 (-10.6 to 2.4)	0.3 (-6.5 to 7.1)	-9.5 (-16.6 to 2.5)	0.05
**Calcium, mg**				
Difference at 3 months	-9.1 (-444.0 to 425.8)	-145.8 (-595.9 to 304.4)	136.7 (-489.2 to 762.6)	0.66
Difference at 3 months^2	-60.4 (-504.7 to 323.1)	-90.8 (-504.7 to 323.1)	30.4 (-546.7 to 607.5)	0.92
Difference at 6 months	-28.3 (-395.5 to 338.9)	-468.6 (-848.7 to -88.5)	440.3 (-88.2 to 968.8)	0.10
Difference at 6 months^2	-87.6 (-392.2 to 217.0)	-405.1 (-720.5 to -89.8)	317.5 (-122.2 to 757.2)	0.15
**Magnesium, mg**				
Difference at 3 months	-11.2 (-93.5 to 71.1)	-70.5 (-155.7 to 14.7)	59.3 (-59.2 to 177.8)	0.32
Difference at 3 months^2	-25.2 (-91.7 to 41.3)	-55.5 (-124.4 to 13.3)	30.4 (65.6 to 123.4)	0.53
Difference at 6 months	5.7 (-79.2 to 90.7)	-114.0 (-201.9 to -26.0)	119.7 (-2.6 to 242.0)	0.06
Difference at 6 months^2	-9.5 (-75.8 to 56.8)	-97.6 (-166.3 to -29.0)	88.1 (-7.6 to 183.8)	0.07
**Sodium, mg**				
Difference at 3 months	-387.8 (-1593.8 to 818.2)	-1523.2 (-2771.5 to 818.2)	1135.4 (-600.3 to 2871.1)	0.20
Difference at 3 months^2	-650.1 (-1425.8 to 125.6)	-1242.2 (-2045.3 to 439.1)	592.1 (-527.7 to 1711.9)	0.29
Difference at 6 months	-396.3 (-1163.1 to 1955.8)	-1817.0 (-3431.2 to 202.8)	2213.3 (-31.1 to 4457.7)	0.05
Difference at 6 months^2	125.2 (-1116.8 to 1367.2)	-1526.5 (-2812.3 to 240.6)	1651.7 (-141.3 to 3444.6)	0.07
**Potassium, mg**				
Difference at 3 months	-60.9 (-775.2 to 653.3)	-599.4 (-1338.7 to 139.9)	538.5 (-489.5 to 1566.5)	0.30
Difference at 3 months^2	-179.0 (-764.4 to 406.3)	-472.9 (-1078.9 to 133.1)	293.9 (-551.1 to 1138.8)	0.49
Difference at 6 months	-68.6 (-665.4 to 802.7)	-854.5 (-1614.3 to 94.7)	923.1 (-133.4 to 1979.6)	0.09
Difference at 6 months^2	-56.1 (-648.7 to 536.4)	-720.8 (-1334.3 to 107.4)	664.7 (-190.7 to 1520.1)	0.13
**Iron, mg**				
Difference at 3 months	-0.8 (-6.7 to 5.0)	-5.2 (-11.2 to 0.9)	4.4 (-4.1 to 12.8)	0.30
Difference at 3 months^2	-1.9 (-6.4 to 2.7)	-4.1 (-8.8 to 0.7)	2.2 (-4.4 to 8.8)	0.51
Difference at 6 months	1.1 (-4.8 to 7.0)	-7.8 (-13.9 to -1.7)	9.0 (0.5 to 17.5)	0.04
Difference at 6 months^2	0.03 (-4.5 to 4.5)	-6.7 (-11.3 to -2.0)	6.7 (0.2 to 13.2)	0.04
**Vitamin C, mg**				
Difference at 3 months	-4.0 (-66.5 to 58.4)	-0.4 (-65.0 to 64.3)	-3.7 (-93.6 to 86.2)	0.94
Difference at 3 months^2	-6.2 (-69.0 to 56.6)	1.9 (-63.1 to 66.9)	-8.1 (-98.7 to 82.5)	0.86
Difference at 6 months	0.2 (-42.6 to 43.0)	-8.2 (-52.5 to 36.1)	8.4 (-53.2 to 70.0)	0.79
Difference at 6 months^2	3.7 (-44.8 to 37.4)	-4.1 (-46.6 to 38.4)	0.4 (-58.9 to 59.7)	0.99
**Vitamin D, mcg**				
Difference at 3 months	0.2 (-3.0 to 3.4)	-0.2 (-3.5 to 3.1)	0.4 (-4.2 to 5.0)	0.86
Difference at 3 months^2	-0.1 (-3.1 to 3.0)	0.2 (-3.0 to 3.3)	-0.2 (-4.6 to 4.2)	0.92
Difference at 6 months	-0.5 (-3.4 to 2.4)	-2.4 (-5.4 to 0.6)	1.9 (-2.3 to 6.1)	0.36

Difference at 6 months[2]	-0.8 (-3.5 to 2.0)	-2.1 (-4.9 to 0.8)	1.3 (-2.7 to 5.3)	0.52
**Vitamin E, IU**				
Difference at 3 months	-1.6 (-7.8 to 4.5)	-0.3 (-6.6 to 6.1)	-1.4 (-10.2 to 7.4)	0.76
Difference at 3 months[2]	-2.1 (-8.1 to 3.9)	0.2 (-6.0 to 6.4)	-2.3 (-11.0 to 6.3)	0.60
Difference at 6 months	-1.5 (-5.3 to 2.2)	-2.9 (-6.7 to 1.0)	1.3 (-4.0 to 6.7)	0.62
Difference at 6 months[2]	-2.1 (-5.3 to 1.2)	-2.3 (-5.6 to 1.1)	0.2 (-4.5 to 4.9)	0.93
**Folate, mcg**				
Difference at 3 months	-2.0 (-70.0 to 66.0)	-63.7 (-134.1 to 6.7)	61.7 (-36.2 to 159.5)	0.21
Difference at 3 months[2]	-13.8 (-68.2 to 40.7)	-51.1 (-107.5 to 5.3)	37.4 (-41.2 to 116.0)	0.35
Difference at 6 months	7.5 (-62.2 to 77.2)	-53.9 (-126.0 to 18.2)	61.4 (-38.8 to 161.7)	0.23
Difference at 6 months[2]	-5.1 (-59.2 to 49.1)	-40.5 (-96.5 to 15.6)	35.4 (-42.8 to 113.6)	0.37
**HEI-2015 family score**				
Difference at 3 months	1.8 (-2.0 to 5.6)	2.2 (-1.7 to 6.1)	-0.3 (-5.8 to 5.1)	0.90
Difference at 3 months[2]	2.0 (-1.8 to 5.7)	2.0 (-1.9 to 5.9)	0.0 (-5.5 to 5.4)	0.99
Difference at 6 months	-3.1 (-6.8 to 0.7)	3.5 (-0.4 to 7.4)	-6.6 (-12.1 to -1.0)	0.02
Difference at 6 months[2]	-2.7 (-6.3 to 1.0)	3.1 (-0.7 to 6.9)	-5.7 (-11.2 to 0.3)	0.04

Mean difference variable is low minus (-) high avocado allotment group. HEI-2015, healthy eating index 2015; MUFA, monounsaturated fatty acids; PUFA, polyunsaturated fatty acids.

[1] From unpaired t-test or ANCOVA model (adjusted for baseline total energy intake), where appropriate.

[2] Adjusted for baseline total energy intake.

Supplemental Table S9. Changes in the food group composition of heads of households, per intention-to-treat analysis in the Effects of Avocado Intake on the Nutritional Status of Families Trial (n=72).

	Within-group difference		Mean between-group difference (95% CI)	p-value[1]
	Low Avocado Allotment (n=37)	High Avocado Allotment (n=35)		
	Mean (95% CI)	Mean (95% CI)		
**Fruit, cup equivalents**				
Difference at 3 months	-0.19 (-0.41, 0.03)	0.87 (0.43, 1.31)	-1.06 (-1.54, -0.57)	<0.0001
Difference at 3 months[2]	-0.19 (-0.52, 0.14)	0.87 (0.53, 1.21)	-1.06 (-1.54, -0.58)	<0.0001
Difference at 6 months	-0.15 (-0.48, 0.18)	0.46 (0.03, 0.88)	-0.61 (-1.13, -0.08)	0.02
Difference at 6 months[2]	-0.16 (-0.52, 0.20)	0.47 (0.10, 0.83)	-0.63 (-1.14, -0.12)	0.02
**Vegetables, cup equivalents**				
Difference at 3 months	0.0003 (-0.40, 0.40)	0.59 (-0.02, 1.20)	-0.59 (-1.31, 0.13)	0.11
Difference at 3 months[2]	0.002 (-0.49, 0.50)	0.59 (0.08, 1.10)	-0.58 (-1.30, 0.13)	0.11
Difference at 6 months	-0.19 (-0.54, 0.16)	0.16 (-0.41, 0.73)	-0.35 (-1.00, 0.31)	0.30
Difference at 6 months[2]	-0.19 (-0.65, 0.26)	0.16 (-0.30, 0.63)	-0.36 (-1.01, 0.30)	0.28
**Greens, cup equivalents**				
Difference at 3 months	-0.02 (-0.36, 0.33)	0.48 (-0.01, 0.96)	-0.49 (-1.07, 0.09)	0.09
Difference at 3 months[2]	-0.02 (-0.42, 0.39)	0.48 (0.06, 0.89)	-0.49 (-1.07, 0.09)	0.10
Difference at 6 months	-0.22 (-0.52, 0.08)	0.08 (-0.34, 0.49)	-0.30 (-0.79, 0.20)	0.23
Difference at 6 months[2]	-0.22 (-0.57, 0.12)	0.08 (-0.28, 0.44)	-0.30 (-0.80, 0.20)	0.23
**Legumes, cup equivalents**				
Difference at 3 months	-0.07 (-0.15, -0.0002)	0.01 (-0.11, 0.13)	-0.08 (-0.22, 0.06)	0.24
Difference at 3 months[2]	-0.07 (-0.17, 0.02)	0.01 (-0.09, 0.11)	-0.08 (-0.22, 0.06)	0.23
Difference at 6 months	-0.01 (-0.10, 0.09)	-0.05 (-0.12, 0.01)	0.04 (-0.07, 0.16)	0.46
Difference at 6 months[2]	-0.01 (-0.09, 0.07)	-0.05 (-0.13, 0.03)	0.04 (-0.08, 0.16)	0.50
**Dairy, cup equivalents**				
Difference at 3 months	-0.08 (-0.39, 0.23)	-0.55 (-1.12, 0.03)	0.47 (-0.17, 1.11)	0.15
Difference at 3 months[2]	-0.11 (-0.49, 0.28)	-0.52 (-0.91, 0.13)	0.42 (-0.13, 0.96)	0.14
Difference at 6 months	-0.02 (-0.47, 0.43)	-0.78 (-1.38, -0.18)	0.76 (0.03, 1.49)	0.04
Difference at 6 months[2]	-0.05 (-0.49, 0.40)	-0.74 (-1.20, -0.29)	0.70 (0.06, 1.34)	0.03
**Nuts, ounce equivalents**				
Difference at 3 months	-0.27 (-0.58, 0.04)	-0.47 (-1.34, 0.40)	0.20 (-0.72, 1.11)	0.70
Difference at 3 months[2]	-0.31 (-0.82, 0.20)	-0.42 (-0.94, 0.10)	0.11 (-0.62, 0.84)	0.77
Difference at 6 months	-0.28 (-0.55, -0.02)	-0.65 (-1.54, 0.23)	0.37 (-0.55, 1.29)	0.42
Difference at 6 months[2]	-0.33(-0.82, 0.16)	-0.60 (-1.11, -0.10)	0.28 (-0.43, 0.98)	0.44

**Whole grains, ounce equivalents**				
Difference at 3 months	-0.11 (-0.56, 0.34)	-0.41 (-0.85, 0.03)	0.30 (-0.32, 0.92)	0.34
Difference at 3 months[2]	-0.14 (-0.49, 0.21)	-0.38 (-0.74, -0.02)	0.24 (-0.27, 0.75)	0.35
Difference at 6 months	-0.37 (-0.77, 0.03)	-0.64 (-1.24, -0.03)	0.27 (-0.45, 0.98)	0.46
Difference at 6 months[2]	-0.41 (-0.78, -0.03)	-0.60 (-0.98, -0.21)	0.19 (-0.35, 0.73)	0.49
**Refined grains, ounce equivalents**				
Difference at 3 months	-1.12 (-2.43, 0.19)	-1.10 (-2.34, 0.13)	-0.02 (-1.79, 1.75)	0.98
Difference at 3 months[2]	-1.19 (-2.31, -0.06)	-1.04 (-2.19, 0.12)	-0.15 (-1.76, 1.46)	0.85
Difference at 6 months	-1.28 (-2.58, 0.03)	-1.45 (-2.61, -0.29)	0.17 (-1.55, 1.89)	0.84
Difference at 6 months[2]	-1.34 (-2.40, -0.29)	-1.37 (-2.46, -0.29)	0.03 (-1.49, 1.55)	0.97
**Processed meats, ounce equivalents**				
Difference at 3 months	-0.06 (-0.21, 0.09)	-0.08 (-0.27, 0.10)	0.02 (-0.21, 0.25)	0.85
Difference at 3 months[2]	-0.06 (-0.23, 0.10)	-0.08 (-0.25, 0.09)	0.02 (-0.21, 0.25)	0.88
Difference at 6 months	0.03 (-0.29, 0.35)	-0.14 (-0.32, 0.04)	0.17 (-0.19, 0.53)	0.35
Difference at 6 months[2]	0.03 (-0.23, 0.28)	-0.14 (-0.40, 0.12)	0.17 (-0.20, 0.53)	0.37
**Chicken and eggs, ounce equivalents**				
Difference at 3 months	-0.16 (-0.39, 0.07)	-0.03 (-0.39, 0.33)	-0.13 (-0.55, 0.29)	0.54
Difference at 3 months[2]	-0.16 (-0.45, 0.13)	-0.02 (-0.32, 0.28)	-0.14 (-0.56, 0.28)	0.50
Difference at 6 months	-0.03 (-0.39, 0.33)	-0.02 (-0.51, 0.47)	-0.01 (-0.60, 0.59)	0.98
Difference at 6 months[2]	-0.03 (-0.45, 0.39)	-0.01 (-0.44, 0.41)	-0.02 (-0.61, 0.58)	0.96
**Fish, ounce equivalents**				
Difference at 3 months	0.13 (-0.16, 0.41)	0.30 (-0.06, 0.67)	-0.18 (-0.63, 0.28)	0.44
Difference at 3 months[2]	0.12 (-0.20, 0.43)	0.31 (-0.01, 0.64)	-0.19 (-0.64, 0.26)	0.40
Difference at 6 months	-0.09 (-0.25, 0.06)	0.15 (-0.30, 0.60)	-0.24 (-0.72, 0.23)	0.31
Difference at 6 months[2]	-0.09 (-0.42, 0.23)	0.15 (-0.18, 0.48)	-0.24 (-0.71, 0.22)	0.30
**Red meat, ounce equivalents**				
Difference at 3 months	-0.03 (-0.36, 0.30)	-0.28 (-0.65, 0.09)	0.25 (-0.23, 0.74)	0.30
Difference at 3 months[2]	-0.04 (-0.37, 0.29)	-0.27 (-0.61, 0.07)	0.23 (-0.24, 0.70)	0.33
Difference at 6 months	-0.35 (-0.65, -0.04)	-0.38 (-0.75, -0.01)	0.03 (-0.43, 0.50)	0.89
Difference at 6 months[2]	-0.36 (-0.67, -0.05)	-0.37 (-0.69, -0.05)	0.01 (-0.44, 0.45)	0.97
**Sugar, teaspoon equivalents**				
Difference at 3 months	-1.10 (-2.41, 0.21)	-3.61 (-7.23, 0.02)	2.51 (-1.32, 6.33)	0.19
Difference at 3 months[2]	-1.30 (-3.26, 0.65)	-3.39 (-5.40, -1.38)	2.09 (-0.71, 4.89)	0.14
Difference at 6 months	-1.48 (-3.32, 0.36)	-4.58 (-8.53, -0.62)	3.09 (-1.22, 7.41)	0.16
Difference at 6 months[2]	-1.72 (-3.90, 0.46)	-4.33 (-6.56, -2.09)	2.61 (-0.52, 5.73)	0.10
**Oils, g**				
Difference at 3 months	-4.66 (-10.78, 1.46)	-1.16 (-8.54, 6.23)	-3.50 (-12.88, 5.87)	0.46

Difference at 3 months[2]	-5.01 (-10.88, 0.85)	-0.78 (-6.81, 5.25)	-4.23 (-12.65, 4.18)	0.32
Difference at 6 months	-8.70 (-14.34, -3.07)	-7.62 (-15.14, -0.11)	-1.08 (-10.24, 8.08)	0.81
Difference at 6 months[2]	-9.22 (-13.98, -4.46)	-7.08 (-11.97, -2.19)	-2.14 (-8.96, 4.69)	0.53
**Soymilk, cup equivalents**				
Difference at 3 months	-0.05 (-0.20, 0.10)	0.07 (-0.07, 0.22)	-0.12 (-0.33, 0.09)	0.25
Difference at 3 months[2]	-0.05 (-0.19, 0.10)	0.07 (-0.08, 0.22)	-0.12 (-0.33, 0.09)	0.26
Difference at 6 months	-0.12 (-0.24, -0.0001)	-0.01 (-0.16, 0.13)	-0.10 (-0.28, 0.08)	0.25
Difference at 6 months[2]	-0.12 (-0.24, 0.01)	-0.02 (-0.15, 0.11)	-0.10 (-0.28, 0.08)	0.26
**Soy, ounce equivalents**				
Difference at 3 months	0.03 (-0.27, 0.34)	0.03 (-0.32, 0.38)	0.002 (-0.46, 0.46)	0.99
Difference at 3 months[2]	0.03 (-0.29, 0.36)	0.03 (-0.30, 0.36)	0.003 (-0.46, 0.47)	0.99
Difference at 6 months	-0.20 (-0.44, 0.04)	-0.07 (-0.37, 0.24)	-0.13 (-0.51, 0.25)	0.50
Difference at 6 months[2]	-0.20 (-0.46, 0.07)	-0.07 (-0.34, 0.20)	-0.13 (-0.51, 0.26)	0.51

Mean difference variable is low minus (-) high avocado allotment group.

[1] From unpaired t-test or ANCOVA model (adjusted for baseline total energy intake), where appropriate.

[2] Adjusted for baseline total energy intake.

Supplemental Table S10. Changes in food group composition of other adults, per intention-to-treat analysis in the Effects of Avocado Intake on the Nutritional Status of Families Trial (n=69).

	Within-group difference		Mean between-group difference (95% CI)	p-value[1]
	Low Avocado Allotment (n=36)	High Avocado Allotment (n=32)		
	Mean (95% CI)	Mean (95% CI)		
**Fruit, cup equivalents**				
Difference at 3 months	-0.01 (-0.24 to 0.23)	-0.11 (-0.51 to 0.30)	0.10 (-0.36 to 0.56)	0.65
Difference at 3 months[2]	-0.07 (-0.32 to 0.18)	-0.04 (-0.31 to 0.24)	-0.03 (-0.41 to 0.34)	0.86
Difference at 6 months	0.08 (-0.25 to 0.41)	-0.13 (-0.65 to 0.40)	0.21 (-0.40 to 0.82)	0.49
Difference at 6 months[2]	0.03 (-0.35 to 0.40)	-0.07 (-0.47 to 0.34)	0.09 (-0.47 to 0.65)	0.75
**Vegetables, cup equivalents**				
Difference at 3 months	-0.24 (-0.51 to 0.02)	-0.01 (-0.55 to 0.53)	-0.23 (-0.82 to 0.36)	0.44
Difference at 3 months[2]	-0.29 (-0.66 to 0.07)	0.05 (-0.34 to 0.44)	-0.34 (-0.88 to 0.20)	0.21
Difference at 6 months	-0.20 (-0.54 to 0.15)	-0.38 (-0.77 to 0.01)	0.19 (-0.32 to 0.70)	0.47
Difference at 6 months[2]	-0.25 (-0.57 to 0.06)	-0.31 (-0.65 to 0.02)	0.06 (-0.40 to 0.52)	0.79
**Greens, cup equivalents**				
Difference at 3 months	-0.12 (-0.30 to 0.06)	-0.03 (-0.37 to 0.30)	-0.09 (-0.46 to 0.29)	0.65
Difference at 3 months[2]	-0.16 (-0.38 to 0.07)	0.01 (-0.24 to 0.25)	-0.17 (-0.50 to 0.17)	0.33
Difference at 6 months	-0.08 (-0.30 to 0.15)	-0.23 (-0.47 to 0.01)	0.15 (-0.17 to 0.48)	0.36
Difference at 6 months[2]	-0.11 (-0.32 to 0.09)	-0.19 (-0.40 to 0.03)	0.07 (-0.22 to 0.37)	0.62
**Legumes, cup equivalents**				
Difference at 3 months	-0.07 (-0.16 to 0.01)	-0.11 (-0.25 to 0.03)	0.04 (-0.12 to 0.20)	0.65
Difference at 3 months[2]	-0.09 (-0.19 to 0.01)	-0.09 (-0.20 to 0.01)	0.002 (-0.14 to 0.14)	0.98
Difference at 6 months	-0.06 (-0.15 to 0.03)	-0.17 (-0.33 to -0.004)	0.11 (-0.08 to 0.29)	0.25
Difference at 6 months[2]	-0.08 (-0.19 to 0.03)	-0.14 (-0.26 to -0.03)	0.06 (-0.10 to 0.22)	0.45
**Dairy, cup equivalents**				
Difference at 3 months	-0.12 (-0.77 to 0.53)	-0.82 (-1.55 to -0.09)	0.70 (-0.26 to 1.66)	0.15
Difference at 3 months[2]	-0.26 (-0.80 to 0.28)	-0.66 (-1.24 to -0.09)	0.40 (-0.40 to 1.20)	0.32
Difference at 6 months	-0.08 (-0.61 to 0.45)	-0.75 (-1.41 to -0.10)	0.68 (-0.14 to 1.49)	0.10
Difference at 6 months[2]	-0.20 (-0.66 to 0.27)	-0.62 (-1.12 to -0.12)	0.42 (-0.27 to 1.11)	0.22
**Nuts, ounce equivalents**				
Difference at 3 months	-0.03 (-0.19 to 0.14)	0.10 (-0.12 to 0.32)	-0.13 (-0.40 to 0.14)	0.33
Difference at 3 months[2]	-0.03 (-0.21 to 0.15)	0.10 (-0.09 to 0.30)	-0.13 (-0.40 to 0.14)	0.33
Difference at 6 months	0.19 (-0.27 to 0.64)	-0.20 (-0.40 to 0.01)	0.39 (-0.11 to 0.88)	0.12
Difference at 6 months[2]	0.19 (-0.17 to 0.54)	-0.20 (-0.58 to 0.19)	0.38 (-0.14 to 0.90)	0.15

**Whole grains, ounce equivalents**				
Difference at 3 months	0.07 (-0.27 to 0.41)	-0.38 (-0.90 to 0.13)	0.45 (-0.14 to 1.04)	0.13
Difference at 3 months[2]	-0.03 (-0.34 to 0.28)	-0.27 (-0.60 to 0.07)	0.24 (-0.22 to 0.70)	0.30
Difference at 6 months	-0.13 (-0.44 to 0.18)	-0.57 (-1.07 to -0.08)	0.44 (-0.13 to 1.02)	0.13
Difference at 6 months[2]	-0.21 (-0.52 to 0.10)	-0.48 (-0.81 to -0.14)	0.26 (-0.19 to 0.72)	0.25
**Refined grains, ounce equivalents**				
Difference at 3 months	-0.92 (-2.02 to 0.17)	-3.67 (-6.86 to -0.48)	2.75 (-0.61 to 6.10)	0.11
Difference at 3 months[2]	-1.58 (-2.78 to -0.39)	-2.91 (-0.44 to 3.09)	1.32 (-0.44 to 3.09)	0.14
Difference at 6 months	-0.28 (-1.49 to 0.94)	-3.90 (-6.61 to -1.19)	3.62 (0.69 to 6.56)	0.02
Difference at 6 months[2]	-0.84 (-1.98 to 0.29)	-3.24 (-4.47 to -2.02)	2.40 (0.72 to 4.08)	0.01
**Processed meats, ounce equivalents**				
Difference at 3 months	-0.06 (-0.30 to 0.17)	-0.50 (-1.07 to 0.06)	0.44 (-0.17 to 1.04)	0.15
Difference at 3 months[2]	-0.16 (-0.46 to 0.13)	-0.39 (-0.71 to -0.07)	0.23 (-0.21 to 0.66)	0.30
Difference at 6 months	-0.02 (-0.15 to 0.12)	-0.26 (-0.84 to 0.32)	0.24 (-0.35 to 0.83)	0.41
Difference at 6 months[2]	-0.08 (-0.42 to 0.27)	-0.19 (-0.56 to 0.17)	0.12 (-0.39 to 0.62)	0.64
**Chicken and eggs, ounce equivalents**				
Difference at 3 months	0.14 (-0.23 to 0.51)	-0.62 (-1.10 to -0.15)	0.76 (0.18 to 1.35)	0.01
Difference at 3 months[2]	0.08 (-0.28 to 0.44)	-0.55 (-0.93 to -0.16)	0.62 (-0.09 to 1.16)	0.02
Difference at 6 months	0.46 (-0.18 to 1.10)	-0.79 (-1.35 to -0.23)	1.25 (0.41 to 2.09)	0.004
Difference at 6 months[2]	0.39 (-0.16 to 0.95)	-0.71 (-1.31 to -0.12)	1.11 (-0.29 to 1.92)	0.01
**Fish, ounce equivalents**				
Difference at 3 months	0.10 (-0.37 to 0.57)	-0.18 (-0.81 to 0.44)	0.28 (-0.48 to 1.04)	0.46
Difference at 3 months[2]	0.03 (-0.46 to 0.52)	-0.10 (-0.63 to 0.42)	0.14 (-0.59 to 0.86)	0.71
Difference at 6 months	0.31 (-0.22 to 0.84)	-0.62 (-1.13 to -0.10)	0.93 (0.20 to 1.66)	0.01
Difference at 6 months[2]	0.24 (-0.23 to 0.71)	-0.54 (-1.05 to -0.03)	0.79 (0.09 to 1.49)	0.03
**Red meat, ounce equivalents**				
Difference at 3 months	0.03 (-0.26 to 0.33)	-0.64 (-1.47 to 0.20)	0.67 (-0.21 to 1.55)	0.13
Difference at 3 months[2]	-0.11 (-0.53 to 0.32)	-0.48 (-0.93 to -0.02)	0.37 (-0.25 to 1.00)	0.24
Difference at 6 months	0.25 (-0.11 to 0.61)	-0.83 (-1.68 to 0.01)	1.08 (0.17 to 1.99)	0.02
Difference at 6 months[2]	0.11 (-0.36 to 0.58)	-0.68 (-1.19 to -0.18)	0.79 (0.10 to 1.49)	0.03
**Sugar, teaspoon equivalents**				
Difference at 3 months	-1.57 (-3.68 to 0.53)	-5.95 (-11.17 to -0.72)	4.37 (-1.21 to 9.96)	0.12
Difference at 3 months[2]	-2.17 (-5.40 to 1.06)	-5.25 (-8.73 to -1.78)	3.08 (-1.69 to 7.85)	0.20
Difference at 6 months	-0.71 (-2.42 to 1.00)	-4.10 (-7.53 to -0.67)	3.38 (-0.41 to 7.17)	0.08
Difference at 6 months[2]	-1.15 (-3.34 to 1.04)	-3.59 (-5.95 to -1.24)	2.44 (-0.78 to 5.67)	0.14
**Oils, g**				
Difference at 3 months	2.91 (-1.46 to 7.28)	-10.57 (-18.79 to -2.34)	13.48 (4.29 to 22.67)	0.005

Difference at 3 months[2]	1.39 (-3.05 to 5.83)	-8.81 (-13.58 to -4.03)	10.20 (3.65 to 16.75)	0.003
Difference at 6 months	3.61 (-0.81 to 8.03)	-12.94 (-22.09 to -3.80)	16.55 (6.51 to 26.59)	0.002
Difference at 6 months[2]	1.91 (-2.79 to 6.60)	-10.98 (-16.02 to -5.93)	12.89 (5.97 to 19.80)	0.0004
**Soymilk, cup equivalents**				
Difference at 3 months	0.01 (-0.01 to 0.03)	0.06 (-0.04 to 0.15)	-0.04 (-0.14 to 0.05)	0.36
Difference at 3 months[2]	0.01 (-0.05 to 0.07)	0.05 (-0.01 to 0.12)	-0.04 (-0.13 to 0.05)	0.36
Difference at 6 months	0.06 (-0.06 to 0.18)	0.06 (-0.01 to 0.13)	0.0003 (-0.14 to 0.14)	0.99
Difference at 6 months[2]	0.06 (-0.04 to 0.16)	0.06 (-0.05 to 0.17)	-0.002 (-0.15 to 0.15)	0.98
**Soy, ounce equivalents**				
Difference at 3 months	-0.02 (-0.21 to 0.16)	0.14 (-0.02 to 0.29)	-0.16 (-0.40 to 0.08)	0.20
Difference at 3 months[2]	-0.02 (0.18 to 0.15)	0.13 (-0.05 to 0.31)	-0.15 (-0.39 to 0.10)	0.23
Difference at 6 months	0.07 (-0.24 to 0.37)	0.10 (-0.10 to 0.31)	-0.04 (-0.40 to 0.33)	0.85
Difference at 6 months[2]	0.08 (-0.18 to 0.33)	0.09 (-0.18 to 0.37)	-0.02 (-0.40 to 0.36)	0.91

Mean difference variable is low minus (-) high avocado allotment group.

[1] From unpaired t-test or ANCOVA model (adjusted for baseline total energy intake), where appropriate.

[2] Adjusted for baseline total energy intake.

Supplemental Table S11. Changes in food group composition of adolescents, per intention-to-treat analysis in the Effects of Avocado Intake on the Nutritional Status of Families Trial (n=32).

	Within-group difference		Mean between-group difference (95% CI)	p-value[1]
	Low Avocado Allotment (n=14)	High Avocado Allotment (n=18)		
	Mean (95% CI)	Mean (95% CI)		
**Fruit, cup equivalents**				
Difference at 3 months	-0.04 (-0.69 to 0.62)	0.19 (-0.37 to 0.75)	-0.22 (-1.04 to 0.59)	0.58
Difference at 3 months[2]	-0.07 (-0.68 to 0.55)	0.21 (-0.33 to 0.75)	-0.28 (-1.10 to 0.54)	0.50
Difference at 6 months	-0.13 (-0.61 to 0.35)	-0.16 (-0.80 to 0.48)	0.03 (-0.78 to 0.85)	0.93
Difference at 6 months[2]	-0.12 (-0.75 to 0.50)	-0.16 (-0.71 to 0.38)	0.04 (-0.79 to 0.88)	0.92
**Vegetables, cup equivalents**				
Difference at 3 months	0.15 (-0.32 to 0.62)	0.002 (-0.29 to 0.30)	0.15 (-0.36 to 0.66)	0.55
Difference at 3 months[2]	0.14 (-0.24 to 0.53)	0.01 (-0.33 to 0.35)	0.14 (-0.38 to 0.65)	0.60
Difference at 6 months	0.53 (-0.61 to 1.67)	-0.04 (-0.47 to 0.39)	0.57 (-0.63 to 1.77)	0.33
Difference at 6 months[2]	0.48 (-0.30 to 1.26)	-0.002 (-0.69 to 0.69)	0.48 (-0.56 to 1.53)	0.35
**Greens, cup equivalents**				
Difference at 3 months	0.10 (-0.24 to 0.44)	-0.02 (-0.26 to 0.23)	0.12 (-0.27 to 0.50)	0.54
Difference at 3 months[2]	0.10 (-0.19 to 0.40)	-0.02 (-0.28 to 0.24)	0.12 (-0.28 to 0.51)	0.55
Difference at 6 months	0.53 (-0.48 to 1.54)	-0.10 (-0.36 to 0.16)	0.63 (-0.40 to 1.66)	0.21
Difference at 6 months[2]	0.49 (-0.16 to 1.15)	-0.07 (-0.65 to 0.51)	0.56 (-0.31 to 1.44)	0.20
**Legumes, cup equivalents**				
Difference at 3 months	-0.08 (-0.25 to 0.08)	-0.06 (-0.22 to 0.10)	-0.02 (-0.25 to 0.20)	0.84
Difference at 3 months[2]	-0.09 (-0.26 to 0.09)	-0.06 (-0.21 to 0.10)	-0.03 (-0.26 to 0.20)	0.81
Difference at 6 months	-0.07 (-0.23 to 0.09)	0.01 (-0.20 to 0.23)	-0.08 (-0.35 to 0.19)	0.55
Difference at 6 months[2]	-0.07 (-0.28 to 0.13)	0.02 (-0.17 to 0.20)	-0.09 (-0.37 to 0.19)	0.51
**Dairy, cup equivalents**				
Difference at 3 months	-0.43 (-1.93 to 1.06)	-0.24 (-0.81 to 0.34)	-0.20 (-1.76 to 1.37)	0.79
Difference at 3 months[2]	-0.36 (-1.37 to 0.66)	-0.29 (-1.19 to 0.60)	-0.07 (-1.42 to 1.29)	0.92
Difference at 6 months	-0.49 (-1.50 to 0.51)	-0.40 (-0.91 to 0.10)	-0.09 (-1.18 to 1.00)	0.87
Difference at 6 months[2]	-0.44 (-1.17 to 0.29)	-0.45 (-1.09 to 0.20)	0.004 (-0.97 to 0.98)	0.99
**Nuts, ounce equivalents**				
Difference at 3 months	0.03 (-0.50 to 0.57)	0.02 (-0.20 to 0.25)	0.01 (-0.56 to 0.57)	0.98
Difference at 3 months[2]	0.05(-0.34 to 0.43)	0.01 (-0.33 to 0.35)	0.04 (-0.48 to 0.55)	0.89
Difference at 6 months	-0.20 (-0.59 to 0.20)	-0.03 (-0.21 to 0.16)	-0.17 (-0.59 to 0.25)	0.42
Difference at 6 months[2]	-0.20 (-0.49 to 0.09)	-0.02 (-0.28 to 0.24)	-0.18 (-0.57 to 0.22)	0.37

**Whole grains, ounce equivalents**				
Difference at 3 months	0.26 (-0.56 to 1.08)	-0.84 (-1.54 to -0.14)	1.10 (0.08 to 2.13)	0.04
Difference at 3 months[2]	0.34 (-0.37 to 1.05)	-0.90 (-1.52 to -0.28)	1.24 (0.29 to 2.19)	0.01
Difference at 6 months	0.23 (-0.84 to 1.29)	-0.93 (-1.56 to -0.29)	1.15 (0.03 to 2.28)	0.05
Difference at 6 months[2]	0.27 (-0.57 to 1.11)	-0.96 (-1.70 to -0.22)	1.23 (0.11 to 2.36)	0.03
**Refined grains, ounce equivalents**				
Difference at 3 months	0.24 (-2.84 to 3.32)	-2.53 (-4.90 to -0.16)	2.77 (-0.88 to 6.43)	0.13
Difference at 3 months[2]	0.56 (-1.83 to 2.96)	-2.78 (-4.90 to -0.67)	3.35 (0.14 to 6.55)	0.04
Difference at 6 months	-1.23 (-2.38 to -0.07)	-1.81 (-3.97 to 0.35)	0.58 (-1.80 to 2.96)	0.62
Difference at 6 months[2]	-1.02 (-2.75 to 0.71)	-1.97 (-3.49 to -0.44)	0.95 (-1.37 to 3.26)	0.41
**Processed meats, ounce equivalents**				
Difference at 3 months	-0.20 (-0.51 to 0.10)	0.01 (-0.34 to 0.36)	-0.21 (-0.67 to 0.25)	0.36
Difference at 3 months[2]	-0.23 (-0.56 to 0.10)	0.03 (-0.27 to 0.32)	-0.26 (-0.70 to 0.19)	0.25
Difference at 6 months	0.14 (-0.49 to 0.76)	-0.17 (-0.49 to 0.15)	0.31 (-0.37 to 0.99)	0.36
Difference at 6 months[2]	0.10 (-0.34 to 0.54)	-0.14 (-0.53 to 0.25)	0.23 (-0.36 to 0.82)	0.43
**Chicken and eggs, ounce equivalents**				
Difference at 3 months	-0.09 (-0.92 to 0.73)	-0.07 (-0.65 to 0.50)	-0.02 (-0.95 to 0.91)	0.97
Difference at 3 months[2]	-0.12 (-0.82 to 0.59)	-0.06 (-0.68 to 0.57)	-0.06 (-1.01 to 0.88)	0.90
Difference at 6 months	-0.16 (-1.19 to 0.87)	-0.29 (-0.65 to 0.07)	0.13 (-0.94 to 1.20)	0.80
Difference at 6 months[2]	-0.15 (-0.87 to 0.57)	-0.30 (-0.93 to 0.34)	0.15 (-0.82 to 1.11)	0.76
**Fish, ounce equivalents**				
Difference at 3 months	0.36 (-0.98 to 1.71)	-0.28 (-0.68 to 0.13)	0.64 (-0.75 to 2.02)	0.34
Difference at 3 months[2]	0.32 (-0.58 to 1.22)	-0.24 (-1.04 to 0.55)	0.56 (-0.64 to 1.77)	0.35
Difference at 6 months	-0.14 (-0.64 to 0.36)	-0.12 (-0.54 to 0.29)	-0.01 (-0.63 to 0.60)	0.96
Difference at 6 months[2]	-0.14 (-0.61 to 0.33)	-0.12 (-0.54 to 0.29)	-0.02 (-0.65 to 0.62)	0.96
**Red meat, ounce equivalents**				
Difference at 3 months	0.05 (-0.41 to 0.51)	0.59 (-0.99 to 2.16)	-0.54 (-2.15 to 1.08)	0.50
Difference at 3 months[2]	0.06 (-1.30 to 1.42)	0.57 (-0.62 to 1.77)	-0.51 (-2.33 to 1.31)	0.57
Difference at 6 months	-0.13 (-0.40 to 0.15)	0.02 (-0.40 to 0.44)	-0.15 (-0.63 to 0.33)	0.54
Difference at 6 months[2]	-0.14 (-0.53 to 0.25)	0.03 (-0.31 to 0.37)	-0.17 (-0.69 to 0.35)	0.52
**Sugar, teaspoon equivalents**				
Difference at 3 months	-4.86 (-15.44 to 5.72)	-2.56 (-5.16 to 0.05)	-2.31 (-13.09 to 8.48)	0.65
Difference at 3 months[2]	-4.28 (-10.86 to 2.30)	-3.01 (-8.80 to 2.79)	-1.27 (-10.07 to 7.52)	0.77
Difference at 6 months	-7.77 (-17.41 to 1.86)	1.07 (-5.92 to 8.06)	-8.84 (-19.95 to 2.26)	0.11
Difference at 6 months[2]	-6.95 (-14.61 to 0.71)	0.43 (-6.32 to 7.18)	-7.38 (-17.62 to 2.86)	0.15
**Oils, g**				
Difference at 3 months	-1.08 (-13.45 to 11.29)	0.53 (-9.39 to 10.45)	-1.61 (-16.61 to 13.38)	0.83

Difference at 3 months[2]	-1.95 (-12.76 to 8.87)	1.20 (-8.33 to 10.73)	-3.15 (-17.61 to 11.31)	0.66
Difference at 6 months	-1.25 (-7.65 to 5.14)	1.04 (-6.90 to 8.98)	-2.29 (-12.52 to 7.93)	0.65
Difference at 6 months[2]	-1.85 (-9.21 to 5.51)	1.51 (-4.98 to 7.99)	-3.36 (13.20 to 6.49)	0.49
**Soymilk, cup equivalents**				
Difference at 3 months	0	0.08 (-0.02 to 0.17)	-0.08 (-0.17 to 0.02)	0.10
Difference at 3 months[2]	-0.001 (-0.08 to 0.08)	0.08 (0.008 to 0.14)	-0.08 (-0.18 to 0.03)	0.14
Difference at 6 months	0.02 (-0.02 to 0.05)	0.03 (-0.02 to 0.07)	-0.01 (-0.06 to 0.04)	0.62
Difference at 6 months[2]	0.01 (-0.02 to 0.05)	0.03 (-0.01 to 0.06)	-0.01 (-0.07 to 0.04)	0.58
**Soy, ounce equivalents**				
Difference at 3 months	-0.07 (-0.16 to 0.03)	0.41 (0.01 to 0.81)	-0.48 (-0.88 to -0.07)	0.02
Difference at 3 months[2]	-0.07 (-0.41 to 0.27)	0.42 (0.11 to 0.72)	-0.49 (-0.94 to -0.03)	0.04
Difference at 6 months	0.01 (-0.07 to 0.09)	-0.02 (-0.09 to 0.05)	0.03 (-0.07 to 0.13)	0.55
Difference at 6 months[2]	0.01 (-0.07 to 0.09)	-0.02 (-0.09 to 0.05)	0.03 (-0.07 to 0.14)	0.51

Mean difference variable is low minus (-) high avocado allotment group.

[1] From unpaired t-test or ANCOVA model (adjusted for baseline total energy intake), where appropriate.

[2] Adjusted for baseline total energy intake.

Supplemental Table S12. Changes in food group composition of children, per intention-to-treat analysis in the Effects of Avocado Intake on the Nutritional Status of Families Trial (n=58).

	Within-group difference		Mean between-group difference (95% CI)	p-value[1]
	Low Avocado Allotment (n=30)	High Avocado Allotment (n=28)		
	Mean (95% CI)	Mean (95% CI)		
**Fruit, cup equivalents**				
Difference at 3 months	-0.06 (-0.55 to 0.44)	-0.18 (-0.88 to 0.52)	0.12 (-0.70 to 0.95)	0.76
Difference at 3 months[2]	-0.06 (-0.64 to 0.52)	-0.17 (-0.77 to 0.43)	0.11 (-0.73 to 0.95)	0.80
Difference at 6 months	-0.20 (-0.72 to 0.32)	0.03 (-0.62 to 0.67)	-0.23 (-1.04 to 0.57)	0.57
Difference at 6 months[2]	-0.23 (-0.79 to 0.33)	0.06 (-0.52 to 0.63)	-0.29 (-1.09 to 0.52)	0.48
**Vegetables, cup equivalents**				
Difference at 3 months	0.03 (-0.23 to 0.30)	-0.48 (-1.03 to 0.07)	0.51 (-0.09 to 1.12)	0.10
Difference at 3 months[2]	-0.02 (-0.39 to 0.36)	-0.43 (-0.82 to -0.04)	0.41 (-0.13 to 0.95)	0.13
Difference at 6 months	0.10 (-0.18 to 0.38)	-0.27 (-0.84 to 0.31)	0.37 (-0.26 to 1.00)	0.24
Difference at 6 months[2]	0.06 (-0.34 to 0.46)	-0.22 (-0.64 to 0.19)	0.28 (-0.30 to 0.86)	0.33
**Greens, cup equivalents**				
Difference at 3 months	0.05 (-0.14 to 0.24)	-0.32 (-0.74 to 0.10)	0.37 (-0.09 to 0.82)	0.11
Difference at 3 months[2]	0.03 (-0.27 to 0.33)	-0.29 (-0.60 to 0.02)	0.32 (-0.11 to 0.75)	0.14
Difference at 6 months	0.15 (-0.07 to 0.37)	-0.09 (-0.52 to 0.34)	0.24 (-0.24 to 0.71)	0.32
Difference at 6 months[2]	0.13 (-0.19 to 0.44)	-0.06 (-0.39 to 0.26)	0.19 (-0.26 to 0.65)	0.40
**Legumes, cup equivalents**				
Difference at 3 months	-0.02 (-0.08 to 0.03)	-0.08 (-0.16 to -0.001)	0.05 (-0.04 to 0.15)	0.25
Difference at 3 months[2]	-0.03 (-0.09 to 0.04)	-0.07 (-0.14 to -0.01)	0.05 (-0.05 to 0.14)	0.33
Difference at 6 months	-0.01 (-0.08 to 0.06)	-0.10 (-0.17 to -0.03)	0.09 (-0.004 to 0.19)	0.06
Difference at 6 months[2]	-0.01 (-0.08 to 0.05)	-0.10 (-0.17 to -0.03)	0.09 (-0.01 to 0.18)	0.09
**Dairy, cup equivalents**				
Difference at 3 months	0.10 (-0.39 to 0.59)	0.02 (-1.21 to 1.25)	0.08 (-1.23 to 1.39)	0.90
Difference at 3 months[2]	0.02 (-0.82 to 0.86)	0.10 (-0.76 to 0.97)	-0.08 (-1.29 to 1.13)	0.89
Difference at 6 months	-0.04 (-0.56 to 0.48)	-0.80 (-1.93 to 0.34)	0.76 (-0.47 to 1.98)	0.22
Difference at 6 months[2]	-0.12 (-0.90 to 0.65)	-0.70 (-1.51 to 0.10)	0.58 (-0.54 to 1.70)	0.31
**Nuts, ounce equivalents**				
Difference at 3 months	-0.05 (-0.24 to 0.14)	-0.12 (-0.40 to 0.16)	0.07 (-0.26 to 0.39)	0.68
Difference at 3 months[2]	-0.05 (-0.28 to 0.18)	-0.12 (-0.35 to 0.12)	0.06 (-0.27 to 0.39)	0.71
Difference at 6 months	-0.17 (-0.33 to -0.004)	-0.19 (-0.43 to 0.05)	0.02 (-0.26 to 0.30)	0.88
Difference at 6 months[2]	-0.17 (-0.37 to 0.02)	-0.19 (-0.39 to 0.02)	0.01 (-0.27 to 0.30)	0.92

**Whole grains, ounce equivalents**				
Difference at 3 months	-0.32 (-1.03 to 0.39)	-0.35 (-0.96 to 0.27)	0.03 (-0.90 to 0.95)	0.96
Difference at 3 months[2]	-0.39 (-0.99 to 0.20)	-0.27 (-0.88 to 0.35)	-0.12 (-0.98 to 0.74)	0.77
Difference at 6 months	-0.18 (-0.87 to 0.51)	-0.30 (-0.99 to 0.40)	0.12 (-0.84 to 1.07)	0.81
Difference at 6 months[2]	-0.24 (-0.88 to 0.39)	-0.23 (-0.89 to 0.43)	-0.01 (-0.93 to 0.90)	0.97
**Refined grains, ounce equivalents**				
Difference at 3 months	-0.54 (-1.83 to 0.76)	-3.42 (-7.03 to 0.19)	2.88 (-0.92 to 6.69)	0.13
Difference at 3 months[2]	-1.04 (-2.86 to 0.78)	-2.88 (-4.77 to -1.00)	1.84 (-0.78 to 4.47)	0.17
Difference at 6 months	1.69 (-2.09 to 5.48)	-4.04 (-7.65 to -0.43)	5.74 (0.60 to 10.87)	0.03
Difference at 6 months[2]	1.14 (-1.87 to 4.16)	-3.45 (-6.57 to -0.33)	4.59 (0.24 to 8.94)	0.04
**Processed meats, ounce equivalents**				
Difference at 3 months	0.20 (-0.14 to 0.54)	-0.18 (-0.76 to 0.40)	0.38 (-0.28 to 1.04)	0.25
Difference at 3 months[2]	0.14 (-0.25 to 0.53)	-0.11 (-0.51 to 0.29)	0.25 (-0.31 to 0.81)	0.38
Difference at 6 months	0.07 (-0.23 to 0.37)	-0.35 (-0.93 to 0.23)	0.42 (-0.23 to 1.06)	0.20
Difference at 6 months[2]	-0.004 (-0.36 to 0.35)	-0.27 (-0.64 to 0.10)	0.27 (-0.25 to 0.78)	0.30
**Chicken and eggs, ounce equivalents**				
Difference at 3 months	-0.20 (-0.59 to 0.19)	-0.97 (-2.04 to 0.10)	0.77 (-0.36 to 1.90)	0.18
Difference at 3 months[2]	-0.35 (-0.88 to 0.17)	-0.80 (-1.35 to -0.26)	0.45 (-0.31 to 1.21)	0.24
Difference at 6 months	0.49 (-0.74 to 1.72)	-0.88 (-1.99 to 0.23)	1.37 (-0.26 to 3.00)	0.10
Difference at 6 months[2]	0.33 (-0.67 to 1.34)	-0.72 (-1.75 to 0.32)	1.05 (-0.39 to 2.50)	0.15
**Fish, ounce equivalents**				
Difference at 3 months	0.10 (-0.29 to 0.49)	-0.24 (-0.63 to 0.15)	0.34 (-0.21 to 0.88)	0.22
Difference at 3 months[2]	0.07 (-0.30 to 0.43)	-0.20 (-0.58 to 0.17)	0.27 (-0.26 to 0.79)	0.31
Difference at 6 months	0.17 (-0.31 to 0.65)	-0.08 (-0.79 to 0.64)	0.24 (-0.58 to 1.07)	0.56
Difference at 6 months[2]	0.15 (-0.43 to 0.72)	-0.05 (-0.65 to 0.54)	0.20 (-0.63 to 1.03)	0.63
**Red meat, ounce equivalents**				
Difference at 3 months	-0.04 (-0.24 to 0.17)	-0.06 (-0.66 to 0.54)	0.03 (-0.60 to 0.65)	0.93
Difference at 3 months[2]	-0.10 (-0.46 to 0.26)	0.004 (-0.36 to 0.37)	-0.10 (-0.62 to 0.41)	0.69
Difference at 6 months	0.08 (-0.27 to 0.43)	-0.48 (-0.98 to 0.02)	0.56 (-0.03 to 1.16)	0.06
Difference at 6 months[2]	0.01 (-0.33 to 0.35)	-0.41 (-0.76 to -0.06)	0.42 (-0.06 to 0.91)	0.09
**Sugar, teaspoon equivalents**				
Difference at 3 months	-0.22 (-2.15 to 1.72)	-5.23 (-11.40 to 0.96)	5.01 (-1.42 to 11.44)	0.12
Difference at 3 months[2]	-0.91 (-4.45 to 2.63)	-4.49 (-8.15 to -0.82)	3.58 (-1.53 to 8.69)	0.17
Difference at 6 months	0.17 (-2.49 to 2.83)	-5.27 (-10.60 to 0.05)	5.45 (-0.42 to 11.31)	0.07

Difference at 6 months[2]	-0.44 (-3.78 to 2.89)	-4.61 (-8.06 to -1.16)	4.17 (-0.64 to 8.98)	0.09
**Oils, g**				
Difference at 3 months	1.74 (-3.62 to 7.10)	-7.34 (-14.27 to -0.42)	9.08 (0.59 to 17.58)	0.04
Difference at 3 months[2]	1.03 (-4.38 to 6.44)	-6.59 (-12.18 to -0.99)	7.62 (-0.19 to 15.42)	0.06
Difference at 6 months	-2.72 (-6.65 to 1.21)	-4.80 (-13.05 to 3.46)	2.08 (-6.94 to 11.09)	0.64
Difference at 6 months[2]	-3.57 (-8.91 to 1.78)	-3.89 (-9.42 to 1.64)	0.32 (-7.39 to 8.03)	0.93
**Soymilk, cup equivalents**				
Difference at 3 months	0.15 (-0.05 to 0.35)	0.07 (-0.35 to 0.49)	0.08 (-0.38 to 0.54)	0.72
Difference at 3 months[2]	0.16 (-0.15 to 0.47)	0.06 (-0.26 to 0.38)	0.10 (-0.35 to 0.55)	0.66
Difference at 6 months	-0.03 (-0.08 to 0.03)	-0.10 (-0.37 to 0.18)	0.07 (-0.21 to 0.35)	0.62
Difference at 6 months[2]	-0.03 (-0.22 to 0.16)	-0.09 (-0.29 to 0.10)	0.07 (-0.20 to 0.34)	0.62
**Soy, ounce equivalents**				
Difference at 3 months	0.33 (-0.10 to 0.75)	0.02 (-0.89 to 0.93)	0.31 (-0.68 to 1.30)	0.53
Difference at 3 months[2]	0.31 (-0.36 to 0.98)	0.03 (-0.66 to 0.73)	0.28 (-0.69 to 1.25)	0.57
Difference at 6 months	0.04 (-0.18 to 0.26)	-0.36 (-1.00 to 0.28)	0.40 (-0.27 to 1.08)	0.23
Difference at 6 months[2]	0.01 (-0.43 to 0.45)	-0.33 (-0.78 to 0.12)	0.34 (-0.30 to 0.97)	0.29

Mean difference variable is low minus (-) high avocado allotment group.

[1] From unpaired t-test or ANCOVA model (adjusted for baseline total energy intake), where appropriate.

[2] Adjusted for baseline total energy intake.

Supplemental Table S13. Changes in family energy-adjusted[1] food group composition per intention-to-treat analysis in the Effects of Avocado Intake on the Nutritional Status of Families Trial (n=72).

	**Within-group differences**				**Between-group difference**		***p*-value[2]**
	**Low Avocado Allotment (n=37)**		**High Avocado Allotment (n=35)**				
	**Mean**	**95% CI**	**Mean**	**95% CI**	**Mean**	**95% CI**	
**Fruit, cup equivalents**							
Difference at 3 months	-0.02	-0.1, 0.1	0.3	0.2, 0.4	-0.3	-0.5, -0.2	0.0003
Difference at 6 months	-0.02	-0.1, 0.1	0.3	0.2, 0.4	-0.3	-0.5, -0.2	<0.0001
**Vegetables, cup equivalents**							
Difference at 3 months	0.1	-0.1, 0.2	0.3	0.1, 0.4	-0.2	-0.4, 0.04	0.01
Difference at 6 months	0.02	-0.1, 0.4	0.2	0.1, 0.4	-0.2	-0.4, 0.1	0.008
**Greens, cup equivalents**							
Difference at 3 months	-0.1	-0.4, 0.2	0.6	-0.9, -0.3	0.5	0.02, 0.9	0.04
Difference at 6 months	-0.1	-0.4, 0.2	-0.5	-0.8, -0.2	0.4	0.01, 0.8	0.05
**Legumes, cup equivalents**							
Difference at 3 months	-0.02	-0.04, 0.01	-0.01	-0.04, 0.01	-0.004	-0.04, 0.03	0.81
Difference at 6 months	-0.01	-0.03, 0.01	-0.02	-0.04, 0.01	0.01	-0.02, 0.04	0.61
**Dairy, cup equivalents**							
Difference at 3 months	0	-0.1, 0.1	-0.03	-0.2, 0.1	0.03	-0.1, 0.2	0.75
Difference at 6 months	-0.01	-0.2, 0.1	-0.1	-0.2, 0.1	0.1	-0.2, 0.3	0.61
**Nuts, ounce equivalents**							
Difference at 3 months	-0.1	-0.1, 0.03	-0.02	-0.1, 0.1	-0.03	-0.2, 0.1	0.58
Difference at 6 months	-0.03	-0.1, 0.1	-1.0	-0.2, 0.01	0.04	-0.1, 0.2	0.46
**Whole grains, ounce equivalents**							
Difference at 3 months	0.04	-0.1, 0.2	-0.1	-0.2, -0.002	0.2	-0.004, 0.3	0.06
Difference at 6 months	-0.5	-0.2, 0.1	-0.1	-0.2, 0.02	0.1	-0.1, 0.2	0.46
**Refined grains, ounce equivalents**							
Difference at 3 months	-0.2	-0.5, 0.1	-0.4	-0.8, -0.1	0.3	-0.1, 0.7	0.18
Difference at 6 months	-0.1	-0.3, 0.2	-0.4	-0.7, -0.1	0.4	-0.02, 0.7	0.07
**Processed meats, ounce equivalents**							
Difference at 3 months	0.01	-0.1, 0.1	-0.01	-0.1, 0.1	0.02	-0.1, 0.1	0.63
Difference at 6 months	0.03	-0.04, 0.1	-0.01	-0.1, 0.1	0.04	-0.1, 0.1	0.47
**Chicken and eggs, ounce equivalents**							
Difference at 3 months	0.02	-0.1, 0.2	-0.1	-0.2, 0.1	0.1	-0.1, 0.3	0.33
Difference at 6 months	0.1	-0.05, 0.3	-0.04	-0.2, 0.1	0.2	-0.1, 0.4	0.17
**Fish, ounce equivalents**							
Difference at 3 months	0.1	-0.03, 0.2	0.1	-0.02, 0.2	-0.01	-0.2, 0.2	0.93
Difference at 6 months	0.1	-0.1, 0.2	0.1	-0.1, 0.2	0.003	-0.2, 0.2	0.98
**Red meat, ounce equivalents**							

Difference at 3 months	0.1	-0.1, 0.2	0.1	-0.1, 0.2	-0.003	-0.2, 0.2	0.97
Difference at 6 months	0.02	-0.1, 0.1	-0.1	-0.2, 0.03	0.1	-0.1, 0.2	0.20
**Sugar, teaspoon equivalents**							
Difference at 3 months	-0.4	-1.1, 0.2	-0.9	-1.6, -0.2	0.5	-0.5, 1.4	0.34
Difference at 6 months	-0.5	-1.1, 0.1	-0.2	-0.8, 0.5	-0.3	-1.2, 0.5	0.45
**Oils, g**							
Difference at 3 months	0.9	-0.4, 2.3	-0.5	-1.8, 0.9	1.4	-0.5, 3.3	0.15
Difference at 6 months	-0.7	-2.2, 0.8	-0.2	-1.7, 1.3	-0.5	-2.7, 1.6	0.63
**Soymilk, cup equivalents**							
Difference at 3 months	0.003	-0.05, 0.1	0.1	0.01, 0.1	-0.1	-0.1, 0.02	0.13
Difference at 6 months	-0.02	-0.1, 0.02	0.02	-0.03, 0.1	-0.04	-0.1, 0.02	0.21
**Soy, ounce equivalents**							
Difference at 3 months	0.04	-0.1, 0.1	0.1	0.03, 0.2	-0.1	-0.2, 0.05	0.18
Difference at 6 months	-0.1	-0.2, 0.1	0.02	-0.1, 0.1	-0.1	-0.2, 0.1	0.36

Mean difference variable is low minus (-) high avocado allotment group.

[1] Energy adjusted method: nutrient density as proportion (%) of macronutrient per total energy intake and/or nutrient density of macro- and micronutrient as intake of nutrient (in appropriate units)/1000 kcals.

[2] From ANOVA model.
